# Nitrate Reduction for Deaminative Suzuki–Miyaura Coupling of Anilines

**DOI:** 10.1002/anie.202504012

**Published:** 2025-05-28

**Authors:** Chen‐Chen Li, Áron Adorján, Manolis Sofiadis, Tim Schulte, Javier Mateos, Mike Rippegarten, Tobias Ritter

**Affiliations:** ^1^ Max‐Planck‐Institut für Kohlenforschung Kaiser‐Wilhelm‐Platz 1 D‐45470 Mülheim an der Ruhr Germany; ^2^ Institute of Organic Chemistry WTH Aachen University Landoltweg 1 52074 Aachen Germany

**Keywords:** Aniline, Cross coupling, Nitrate, Suzuki–Miyaura coupling

## Abstract

We present a deaminative Suzuki–Miyaura‐type coupling (SMC) of anilines with nitrate as a diazotization reagent, which integrates transition‐metal catalysis with nitrate‐based diazonium chemistry for the first time. The synergistic reduction of nitrate by bisulfite and boronic acids allows for both oxidative diazotization with low‐valent transition metal redox transformations simultaneously. The reaction utilizes low‐hazard, readily available starting materials and reagents. In comparison to previous diazonium‐based Suzuki–Miyaura‐couplings, the in situ oxidation of anilines by reduction of nitrate allows larger functional group tolerance.

## Graphical abstract

We present a one‐step deaminative Suzuki–Miyaura‐type coupling of anilines via fleeting diazonium intermediate with nitrate and bisulfite as the reagents. This represents the first application of the nitrate reduction strategy in transition metal catalysis of diazonium chemistry. The reaction utilizes low‐hazard, readily available starting materials and reagents. Our method demonstrates a simpler and safer procedure, together with the advantage of functional group tolerance and potential application in the arylation of various aniline‐containing complex molecules.

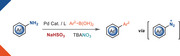

Aryldiazonium salts are synthetically versatile and have been used for conventional bond‐forming reactions such as iodination for over a century.^[^ ^1^, ^2^, ^3^, ^4^, ^5^, ^6^, ^7^ ^]^ They also serve as electrophiles for transition metal‐catalyzed reactions, which has expanded their use over the past decades.^[^ ^8^, ^9^ ^]^ In general, diazonium chemistry is dangerous, but we have published a diazotization reaction via nitrate reduction in 2024, which resulted in safer methodology for diazonium chemistry because the accumulation of aryldiazonium salts is avoided.^[^ ^10^ ^]^ Diazotization with nitrate (NO_3_^−^), in contrast to the conventional nitrite‐based (NO_2_^−^) protocol, requires in situ redox chemistry to obtain the correct oxidation state of nitrogen (N^III^) for diazotization and to avoid arene nitration through electrophilic aromatic substitution.^[^ ^10^, ^11^, ^12^ ^]^ The in situ redox chemistry for nitrate reduction must be compatible with the reaction conditions of further transformations of the diazonium salt. Thermodynamically, nitrate has a higher oxidation potential than nitrite.^[^ ^13^, ^14^ ^]^ Also, in situ formation of highly oxidizing species such as NO_2_ takes place. We had shown that halides are sufficiently inert to tolerate the oxidative conditions required for the initial redox chemistry and remain available for the subsequent redox‐neutral product formation step (Scheme 1a). Based on the reactivity of aryldiazonium salts as electrophiles in cross‐coupling chemistry,^[^ ^8^, ^9^ ^]^ we questioned whether low valent transition metals such as palladium (0) would be compatible with the highly oxidizing intermediates required for in situ diazotization within our nitrate reduction strategy. While, thermodynamically, such an approach seems questionable, we hypothesized that the individual rates of nitrate reduction and oxidative diazotization could be appropriate to be combined with the palladium‐catalyzed bond formation and thus result in a kinetically feasible reaction, which avoids the potential interaction of palladium catalysts with NO*_x_* species.^[^ ^15^, ^16^ ^]^ Here, we report the successful combination of safer diazonium chemistry through nitrate reduction with conventional palladium‐catalyzed Suzuki–Miyaura chemistry^[^ ^17^, ^18^, ^19^, ^20^, ^21^, ^22^ ^]^ to produce biaryls directly from anilines (Scheme 1c). Conceptually, the work shows that low‐valent transition metal catalysis is compatible with oxidative in situ diazonium formation from nitrate, which, overall, increases safety for diazonium‐based Suzuki–Miyaura coupling reactions.^[^ ^23^, ^24^, ^25^, ^26^, ^27^, ^28^, ^29^ ^]^ Due to the formation of the reactive diazonium salt only as a fleeting intermediate,^[^ ^10^ ^]^ a wider variety of functional groups can be accommodated, which is often problematic for stoichiometric diazotization reactions owing to diazonium instability.

**Scheme 1:**
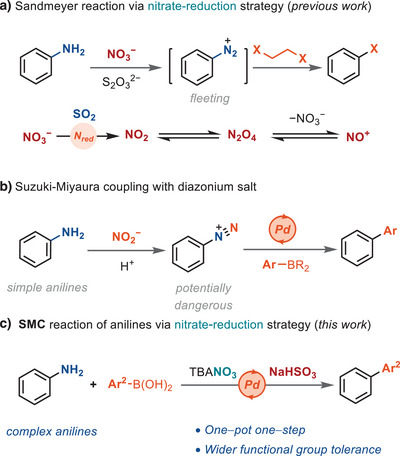
Design of SMC reaction of anilines with nitrate reduction strategy.

In 1977, Matsuda et al. demonstrated the oxidative addition of a diazonium salt to Pd(0) in cross‐coupling reactions.^[^ ^30^, ^31^ ^]^ The Suzuki–Miyaura coupling of isolated diazonium salts was reported by Demoute et al. in 1996^[^ ^23^ ^]^ and Bhattacharyya et al. in 1997 (Scheme 1b).^[^ ^24^ ^]^ Because diazonium salts are hazardous and unstable, chemists tried to avoid the isolation of diazonium salts.^[^ ^4^, ^7^ ^]^ In situ generation of aryldiazoniums is one of the approaches to avoid diazonium salt isolation,^[^ ^32^, ^33^ ^]^ although accumulation can still pose both reaction‐ and safety challenges.^[^ ^34^, ^35^, ^36^ ^]^ Song et al,^[^ ^25^ ^]^ Wang et al.,^[^ ^26^ ^]^ Felpin et al.,^[^ ^28^ ^]^ and Seayad et al.^[^ ^29^ ^]^ demonstrated one‐pot diazotization/SMC reactions without the isolation of the diazonium salts. However, these works required conditions such as acid as solvent, stoichiometric amount of transition metal,^[^ ^29^ ^]^ a privileged substrate,^[^ ^28^ ^]^ or a one‐pot two‐step procedure.^[^ ^25^, ^26^ ^]^ As all pre‐2024 diazotization reactions, this chemistry relies on nitrite as a diazotization reagent, which can result in the accumulation of diazonium salts and the associated safety issues.^[^ ^23^, ^24^, ^25^, ^26^, ^27^, ^28^, ^29^ ^]^ Our group discovered that nitrate can serve as a safer diazotization reagent via in situ nitrate reduction, which in turn enables the in situ generation of diazonium salts as fleeting intermediates for subsequent chemistry.^[^ ^10^ ^]^ The nitrate reduction involves a single electron reduction of NO_3_^−^ to form NO_2_, which dimerizes to N_2_O_4_, and disproportionates to NO_3_^−^ and NO^+^ as the diazotization reagent (Scheme 1a). With the nitrate‐reduction strategy, Sandmeyer halogenation was realized by using SO_2_, released from thiosulfate pentahydrate (Na_2_S_2_O_3_·5H_2_O) in situ, or cuprates as reductant.

To extend the nitrate reduction strategy to the use of transition metal catalysis, the active catalyst must sustain the oxidizing intermediates that result from nitrate reduction,^[^ ^37^, ^38^, ^39^, ^40^ ^]^ for example, the formation of highly oxidative NO_2_.^[^ ^15^, ^16^ ^]^ In general, low‐valent palladium is quickly oxidized by NO_x_ species resulting from the nitrate reduction process.^[^ ^41^, ^42^, ^43^, ^44^ ^]^ Although thermodynamically nitrate is a strong oxidant,^[^ ^13^ ^]^ NO_3_^−^ is a kinetically stabilized anion. A formal one‐electron reduction of nitrate results in the formation of NO_2_ and subsequent dimerization and disproportionation in the highly oxidizing nitrosyl cation N≡O^+^ and nitrate.^[^ ^15^, ^16^ ^]^ In addition to potential oxidation of low‐valent palladium species, reactive nitrogen species could also react with nucleophiles that are employed in conventional transition‐metal‐catalyzed cross‐coupling reactions, such as aryl boronic acids, unproductively.^[^ ^45^, ^46^, ^47^ ^]^ The thiosulfate we had previously used for nitrate reduction may function as a nucleophile to result in undesired C─S bond formation.^[^ ^48^, ^49^, ^50^, ^51^ ^]^ Sulfur‐based reductants may also poison the palladium catalyst.^[^ ^52^, ^53^, ^54^ ^]^ Other challenges included the potential incompatibility of reaction conditions, for example, while conventional diazotization proceeds under acidic conditions, SMC reactions usually proceed with base.^[^ ^17^, ^18^, ^19^, ^20^, ^21^, ^22^ ^]^ Based on our previous research,^[^ ^10^, ^22^ ^]^ we focused on identifying a suitable nitrate reductant that could function in a base‐free SMC reaction, without poisoning the catalyst. The mechanistic analysis for nitrate reduction revealed that slow release of the active reductant SO_2_ was important. While thiosulfate can be converted to SO_2_, the S_2_O_3_^2−^ anion is nucleophilic, which can lead to side reactivity. The terminal sulphur atom in thiosulfate carries a formal oxidation state of −II, while sulfite only contains sulphur in the +IV oxidation state, still a reductant but less nucleophilic: HSO_3_^−^ has a weaker nucleophilicity than S_2_O_3_^2−^ or S_4_O_6_^2−^,^[^ ^55^ ^]^ and releases SO_2_ under acidic conditions. Because nitrate reduction is generally acidic, we chose to leverage our previously obtained knowledge in the context of arylthianthrenium chemistry that transmetallation between boronic acid and Pd‐catalysts can be accomplished even in acidic media when a cationic Pd catalyst, BF_4_^−^ and MeOH are present.^[^ ^22^, ^56^, ^57^, ^58^, ^59^, ^60^, ^61^ ^]^ Although aryl diazonium and thianthrenium chemistry use different starting materials, they share the cationic charge of the aryl electrophile, which results in a cationic, electrophilic Pd(II) intermediates with similar properties. Combination of both concepts enabled us to develop a direct SMC reaction from anilines with nitrate.

Reaction between aniline **1a**, *p*‐fluorophenyl boronic acid (**2a**), tetrabutylammonium nitrate, NaHSO_3_ as reductant, HBF_4_ and MeOH as additives for transmetallation, and Pd(OAc)_2_ with tris‐(4‐trifluoromethylphenyl)phosphine (**L1**) as catalyst in butyronitrile upon heating at 115 °C resulted in a 69% yield of the cross‐coupling product 4‐fluoro‐1,1′‐biphenyl (**3a**) (Scheme 2, entry 1). We observed that the replacement of **L1** with a more electron rich phosphine led to a sharp decline of yield, indicating that the ligand is an essential component in the catalysis (Scheme 2, entry 2). In the SMC of aryl halides, electron‐rich phosphines are commonly used as ligands as they accelerate the oxidative addition step.^[^ ^62^, ^63^, ^64^ ^]^ Because oxidative addition to aryl diazoniums is facile,^[^ ^65^, ^66^ ^]^ electron‐rich phosphines are not required for oxidative addition, and, in fact, the reaction proceeds better with electro‐poor phosphines such as **L1**. Because electron‐rich phosphines are more prone to oxidation,^[^ ^67^, ^68^ ^]^ the superiority of **L1** may be a consequence of its resistance to oxidation under the reaction conditions or to render the in situ formed resting state of the putative palladium‐phosphine complex sufficiently stable in the oxidizing reaction conditions. Although we were not able to discern the exact identity of the phosphine species, we observed fast conversion of electron‐rich triphenylphosphine in the reaction conditions to an unknown, unreactive species, while **L1** remained competent for catalysis (see Figure S19).

**Scheme 2:**
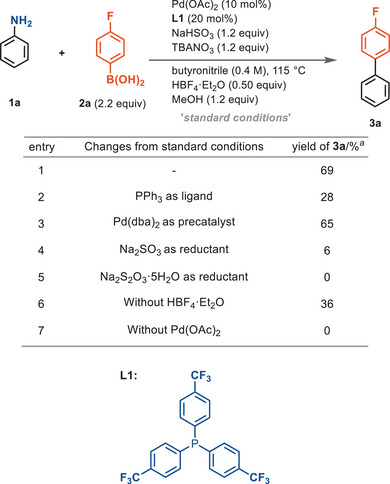
Reaction condition investigation. *^a^* Yields were determined by **^19^F NMR** using α,α,α‐trifluorotoluene as internal the standard.

Switching the palladium precursor from Pd(OAc)_2_ (Pd^II^) to Pd(dba)_2_ (Pd^0^) had minimal effect on the reaction result (Scheme 2, entry 3), consistent with a conventional Pd(0)–Pd(II) cycle of a Suzuki reaction. A competition experiment between a Pd(0) complex and an aniline in their reaction with NO^+^ resulted in competitive conversion of the aniline to a diazonium species, which indicates appropriate relative rates, and may explain the compatibility of low‐valent palladium catalysis with the oxidizing diazotization reaction conditions (see Figure S12). The choice of reductant plays a significant role. When NaHSO_3_ was replaced with Na_2_SO_3_, a more basic S^IV^ species, only 6% of the desired product was obtained (Scheme 2, entry 4). The use of Na_2_S_2_O_3_ as reductant did not lead to the formation of coupling product (Scheme 2, entry 5). The reaction still proceeded in the absence of HBF_4_, but the yield of coupling product dropped by nearly half (Scheme 2, entry 6).

Compared to the well‐developed Suzuki reactions from aryl halides, the current substrate scope for the deaminative process is smaller (Scheme 3), for example, electron‐rich arylboronic acids afford significantly lower yields than obtained with electron‐neutral or poor substrates (**3m**). Yet, when compared to previous SMC that are accomplished from anilines with nitrite oxidants, the nitrate reduction strategy reported here enables a broader substrate scope by avoiding the accumulation of diazonium species. In contrast to previous diazonium‐based Suzuki reactions, aminoheterocycles (**3n**) and anilines with heterocyclic substituents (**3j**, **3k**) are suitable substrates as well. An increased functional group tolerance compared to previous reports was obtained;^[^ ^23^, ^24^, ^25^, ^26^, ^27^, ^28^, ^29^ ^]^ of note, aryl halides both on the aniline and on the arylboronic partner are tolerated, with chemoselective reaction at the diazonium. Several oxidant‐sensitive functional groups can also be tolerated, such as Michael acceptor (**3k**), secondary alcohols (**3p**), and formyl groups (**3c**). When bearing substituents at the ortho‐position of anilines, the reaction still proceeds (**3** **h**, **3i,** and **3l**). Complex anilines, including sulfadoxin (**3j**), coumarin derivative **3k**, Aminoglutethimide (**3o**), and Darunavir (**3p**), can be arylated efficiently with our method. In the gram‐scale (5 mmol scale) reaction between sulfadoxin and 3‐chlorophenyl boronic acid, 2.5 mol % catalyst loading led to similar yield as on 0.5 mmol scale. Substrates containing thiol groups or nitro groups did not afford high yields, likely due to incompatibility with the nitrate reduction system (Figure S22). Anilines bearing certain heterocycles, such as thiophene or tetrazole, are also not effective substrates (Figure S22).

**Scheme 3:**
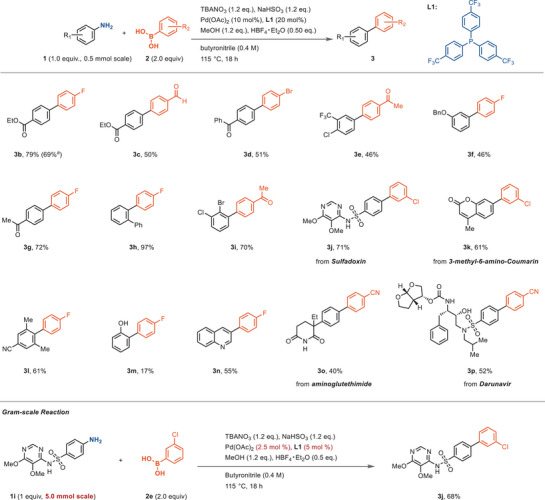
Substrate scope. *^a^* Pd(OAc)_2_ 2.5 mol %, **L1** 5 mol % was used.

The combination of NaHSO_3_ and TBANO_3_, without any other components except solvent, did not result in NO_2_ formation at or below 115 °C (Scheme 4a, entry 1), nor did the addition of anilines or methanol as an additive (Scheme 4a, entry 4, 5). However, acidic components, such as boronic acid and HBF_4_, can mediate nitrate reduction (Scheme 4a, entry 2, 3), possibly by facilitating SO_2_ formation from bisulfite, which can be used for nitrate reduction. NO_2_ was detected by UV–vis spectroscopy (Figure S10). Gas phase IR measurements showed the formation of N_2_O (Figure S11), which could be formed by degradation of NO_2_ and further indicates the reduction of nitrate.^[^ ^69^ ^]^ A proposed catalytic cycle that is consistent with all obtained experimental data is shown in Scheme 4b. Reduction of Pd(OAc)_2_ is achieved through either the homocoupling of the boronic acid^[^ ^70^ ^]^ or the oxidation of **L1**.^[^ ^71^ ^]^ Side products of both reductive mechanisms—the homocoupled boronic acid and the phosphine oxide resulting from **L1**—were detected in the reaction mixture (Figures S15 and S21). The decomposition of the arylboronic acid and **L1** throughout the reaction necessitates the use of excess reagents. With the oxidative addition of aryl diazonium to Pd(0), a cationic intermediate (**A**) is expected to form, similarly to the SMC reaction of aryl thianthrenium salts.^[^ ^22^ ^]^ In a conventional Suzuki reaction, the interaction between boronic acid and a base facilitates transmetallation.^[^ ^55^ ^]^ In this context, boronic acid likely aids in nitrate reduction by acidifying HSO_3_^−^ to generate SO_2_ (see Figure S9). Therefore, we propose that the transmetallation from intermediate **A** to form complex **B** is facilitated by the acid–base interaction between boronic acid and HSO_3_^−^. Besides the interaction of boronic acid with HSO_3_^−^, HBF_4_ could also assist transmetallation by experiencing a similar ionic interaction as the SMC reaction of thianthrenium salt.^[^ ^22^ ^]^ Complex **B** finally conducts reductive elimination to obtain the biaryl product.

**Scheme 4:**
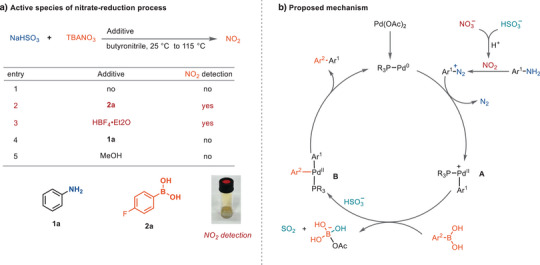
Control experiments and proposed mechanism. NO_2_ was observed by obtaining brown gas formation and further detected by UV–vis spectroscopy (Figure S6).

In summary, by overcoming the incompatibility of the redox chemistry required for nitrate reduction with low‐valent palladium catalysis, the combination of acidic oxidative addition catalysis and safer diazonium chemistry from nitrate reduction resulted in a direct Suzuki–Miyaura reaction from anilines.

## Supporting Information

The authors have cited additional references within the Supporting Information.^[^ ^10^, ^69^, ^72^, ^73^, ^74^ ^]^

## Conflict of Interests

The authors declare no conflict of interest.

## Supporting information



Supporting information

## Data Availability

The data that support the findings of this study are available in the Supporting Information of this article.
