# Supplementary material for: Nitrate Reduction for Deaminative Suzuki–Miyaura Coupling of Anilines
Source: Angew Chem Int Ed Engl. 2025 May 28;64(27):e202504012. doi: 10.1002/anie.202504012 (PMC12207368; doi:10.1002/anie.202504012)
Supplement: Supplementary file 1 — Supporting information [file ANIE-64-e202504012-s001.pdf]

SUPPORTING INFORMATION

Nitrate Reduction for Deaminative Suzuki-Miyaura Coupling of Anilines

Chen-Chen Li,[a] Áron Adorján,[a], [b] Manolis Sofiadis, [a] Tim Schulte,[a] Javier Mateos,[a] Mike Rippegarten,[a] and Tobias Ritter*[a]

[a] Max-Planck-Institut für Kohlenforschung, Kaiser-Wilhelm-Platz 1, D-45470 Mülheim an der Ruhr, Germany

[b] Institute of Organic Chemistry, RWTH Aachen University, Landoltweg 1, 52074 Aachen, Germany.

*E-mail: ritter@kofo.mpg.de

TABLE OF CONTENTS

TABLE OF CONTENTS ... S1

MATERIALS AND METHODS ... S4

EXPERIMENTAL DATA ... S5

General procedure for deaminative Suzuki-Miyaura cross-coupling ... S5

Reaction Condition Screening ... S5

Screening of the palladium precatalyst ... S5

Screening of the acid additive ... S6

Screening of the ligand ... S7

Influence of reductant ... S8

Influence of reaction temperature ... S9

Control experiments ... S10

Influence of nucleophile species on the reaction ... S11

Reactions with other cross-coupling reagents ... S12

Mechanistic studies ... S14

NO_2 and SO_2 detection experiments ... S14

Preparation of diazonium salt (**10a**) ... S17

NO^+ competition experiments ... S18

Analysis of ligand decomposition ... S19

Analysis of reaction with triphenylphosphine ... S22

Preparation of phosphine oxide **9a** ... S24

Control experiment with phosphine oxide **9a** ... S25

Analysis of arylboronic acid homocoupling ... S26

Substrate Scope of Suzuki-Miyaura cross-coupling of anilines. ... S26

Benzocaine-derived biphenyl **3b** ... S26

Benzocaine-derived biphenyl **3c** ... S27

4-Aminobenzophenone-derived biphenyl **3d** ... S28

4-Chloro-3-(trifluoromethyl)aniline-derived biphenyl **3e** ... S28

3-(Benzyloxy)aniline-derived biphenyl **3f** ... S29

4-Aminoacetophenone-derived biphenyl **3g** ... S30

4-Fluoro-1,1':2',1''-terphenyl **3h** ... S30

2-Bromo-3-chloroaniline-derived biphenyl **3i** ... S31

Sulfadoxin-derived biphenyl **3j** ... S32

4-Methylcoumarin-derived biphenyl **3k** ... S33

4-Amino-3,5-dimethylbenzonitrile-derived biphenyl **3l** ... S34

2-Aminophenol-derived biphenyl **3m** S34
3-(4-Fluorophenyl)quinoline **3n** S35
Aminoglutethimide-derived biphenyl **3o** S36
Darunavir-derived biphenyl **3p** S37
Unsuccessful examples S37

SPECTROSCOPIC DATA S40

^{1}H NMR spectrum of **3b** S40
^{13}C NMR spectrum of **3b** S41
^{19}F NMR spectrum of **3b** S42
^{1}H NMR spectrum of **3c** S43
^{13}C NMR spectrum of **3c** S44
^{1}H NMR spectrum of **3d** S45
^{13}C NMR spectrum **3d** S46
^{1}H NMR spectrum of **3e** S47
^{13}C NMR spectrum of **3e** S48
^{19}F NMR spectrum of **3e** S49
^{1}H NMR spectrum of **3f** S50
^{13}C NMR spectrum of **3f** S51
^{19}F NMR spectrum of **3f** S52
^{1}H NMR spectrum of **3g** S53
^{13}C NMR spectrum of **3g** S54
^{19}F NMR spectrum of **3g** S55
^{1}H NMR spectrum of **3h** S56
^{13}C NMR spectrum of **3h** S57
^{1}H NMR spectrum of **3i** S58
^{13}C NMR spectrum of **3i** S59
^{1}H NMR spectrum of **3j** S60
^{13}C NMR spectrum of **3j** S61
^{1}H NMR spectrum of **3k** S62
^{13}C NMR spectrum of **3k** S63
^{1}H NMR spectrum of **3l** S64
^{13}C NMR spectrum of **3l** S65
^{1}H NMR spectrum of **3m** S66
^{13}C NMR spectrum of **3m** S67
^{19}F NMR spectrum of **3m** S68
^{1}H NMR spectrum of **3n** S69
^{13}C NMR spectrum of **3n** S70

^{19}F NMR spectrum of **3n** S71
^{1}H NMR spectrum of **3o** S72
^{13}C NMR spectrum of **3o** S73
^{1}H NMR spectrum of **3p** S74
^{13}C NMR spectrum of **3p** S75
^{1}H NMR spectrum of **9a** S76
^{13}C NMR spectrum of **9a** S77
^{19}F NMR spectrum of **9a** S78
^{31}P NMR spectrum of **9a** S79
^{1}H NMR spectrum of **10a** S80
^{13}C NMR spectrum of **10a** S81
^{19}F NMR spectrum of **10a** S82
^{1}H NMR spectrum of **11a** S83
^{13}C NMR spectrum of **11a** S84
^{19}F NMR spectrum of **11a** S85

REFERENCES S86

MATERIALS AND METHODS

All reactions were carried out under ambient atmosphere. Concentration under reduced pressure was performed by rotary evaporation at 40°C at an appropriate pressure. Purified compounds were further dried under high vacuum (0.010–0.005 mbar). Yields refer to spectroscopically pure compounds, unless otherwise stated.

Solvents

Anhydrous solvents were obtained from Phoenix Solvent Drying Systems. Anhydrous *n*-PrCN was purchased from BLDPharm. All deuterated solvents were purchased from *Euriso-Top*.

Chromatography

Thin layer chromatography (TLC) was performed using EMD TLC plates pre-coated with 250 µm thickness silica gel 60 F254 plates and visualized by fluorescence quenching under 254 nm UV light or wetting with potassium permanganate stain. Flash chromatography was performed using silica gel (40–63 µm particle size) purchased from Geduran® or using a Biotage Isolera Spektra Four system with 10 g or 25 g Sfär chromatography columns. High-resolution mass spectra were obtained using Q Exactive Plus from Thermo.

Spectroscopy and Instruments

NMR spectra were recorded on a Bruker AVANCE III HD 500 spectrometer operating at 500 MHz, 471 MHz, and 126 MHz, for ^{1}H, ^{19}F, and ^{13}C acquisitions, respectively; a Bruker AVANCE NEO 600 spectrometer equipped with a cryogenically cooled cryoBBO probe operating at 600 MHz and 151 MHz for ^{1}H and ^{13}C acquisitions, respectively. ^{1}H and ^{13}C chemical shifts are reported in ppm with the solvent residual peak as the internal standard. For ^{1}H NMR: $CDCl_3$, δ = 7.26 ppm, and for ^{13}C NMR: $CDCl_3$, δ = 77.2 ppm. Data is reported as follows: s = singlet, d = doublet, t = triplet, q = quartet, p = pentet, m = multiplet, br = broad singlet, coupling constants in Hz; integration. Gas IR measurements were performed on a Thermo Scientific Nicolet Avatar 370 FT-IR spectrometer. A background spectrum was recorded before every measurement which was subtracted from the actual spectrum.

Starting materials

All substrates were used as received from commercial suppliers, unless otherwise stated. Chemicals were purchased from Sigma-Aldrich, TCI, Alfa Aesar, BLDPharm, Thermo Fisher Scientific or ChemImpex. Deuterated solvents were purchased from Euriso-Top.

EXPERIMENTAL DATA

Caution: When performing reactions in pressurized systems (such as closed vials, pressure tubes, and autoclaves), a blast shield must be used to minimize personal damage in case of an accident. Although diazonium salt accumulation was not detected in any of the following reactions, as safety precaution all following experiments were performed behind a blast shield.

General procedure for deaminative Suzuki-Miyaura cross-coupling

To a 4-mL vial, $TBANO_3$ (182 mg, 0.600 mmol, 1.20 equiv.), $NaHSO_3$ (62 mg, 0.60 mmol, 1.2 equiv.), $Pd(OAc)_2$ (12 mg, 0.050 mmol, 0.10 equiv.), **L1** (47 mg, 0.10 mmol, 0.20 equiv.), arylboronic acid (1.10 mmol, 2.20 equiv.) and aniline if solid (0.500 mmol, 1.00 equiv.) were added followed by anhydrous *n*-PrCN (1.25 mL, c = 0.400 M) and MeOH (24 µL, 19 mg, 0.60 mmol, 1.2 equiv.) and aniline (0.500 mmol, 1.00 equiv.) if liquid. The suspension was stirred for 5 minutes at 23 °C and then $HBF_4{\cdot}OEt_2$ (34 µL, 40 mg, 0.25 mmol, 0.50 equiv.) was added. The vial was sealed with a septum cap and the reaction mixture was heated at 115 °C and stirred (400 rpm) for 18 h. The reaction mixture was cooled to 23 °C and a sat. aq. $NaHCO_3$ (1 mL) solution was added, and the resulting mixture was extracted by EtOAc (3 × 2 mL). The combined organic phases were passed through a pad of silica gel (2 g), and the solvent was evaporated *in vacuo*. The crude material was purified by flash column chromatography (SiO_2).

Reaction Condition Screening

Screening of the palladium precatalyst

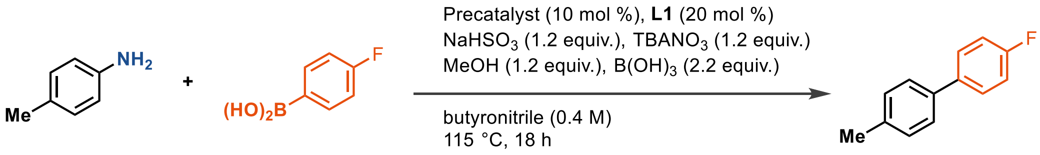

1p (0.2 mmol scale) **2a** (2.2 equiv) **3q**

entry	Precatalyst	yield of **3q**/%[a]
1	$Pd(OAc)_2$	36
2	$Pd(NO_3)_2$	37
3	$Pd(acac)_2$	35
4	$Pd(dba)_2$	38
5	$Pd_2(dba)_3$	21
6	Pd/C (10 %)	19

Fig S1. Screening of the palladium precatalyst. [a]Yields were determined by ^{19}F NMR spectroscopy using α,α,α-trifluorotoluene as internal standard.

Procedure

To a 4-mL vial, $TBANO_3$ (73 mg, 0.24 mmol, 1.2 equiv.), $NaHSO_3$ (25 mg, 0.24 mmol, 1.2 equiv.), palladium

precatalyst (0.020 mmol, 0.10 equiv.), **L1** (18.6 mg, 0.0400 mmol, 0.200 equiv.), *p*-toluidine (18.6 mg, 0.200 mmol, 1.00 equiv.) and **2a** (62 mg, 0.44 mmol, 2.2 equiv.) were added, followed by anhydrous butyronitrile (0.50 mL, c = 0.40 M) and MeOH (9.7 µL, 7.7 mg, 0.24 mmol, 1.2 equiv.). The suspension was stirred for 5 minutes at 23 °C and then $HBF_4 \cdot OEt_2$ (14 µL, 16 mg, 0.10 mmol, 0.50 equiv.) was added. The vial was sealed with a septum cap and the reaction mixture was heated at 115 °C and stirred (400 rpm) for 18 h. The reaction mixture was cooled to 23 °C and a sat. aq. $NaHCO_3$ (1 mL) solution was added, and the resulting mixture was extracted by EtOAc (3 × 2 mL). The combined organic phases were passed through a pad of silica gel (2 g), and the solvent was evaporated *in vacuo*. The yield was determined by ^{19}F NMR spectroscopy at 298 K and 471 MHz, by dissolving the crude material in $CDCl_3$ (0.5 mL) and adding α,α,α-trifluorotoluene (8.2 µL, 9.8 mg, 67 µmol, 0.33 equiv.) as internal standard. The integration of the peak corresponding to the internal standard at −62.9 ppm (3F, s) was compared to the peak corresponding to **3q** at −116.7 ppm (1F, (tt, *J* = 8.7, 5.3 Hz)). The correct peak for **3q** was assigned by comparison to literature.[1]

Screening of the acid additive

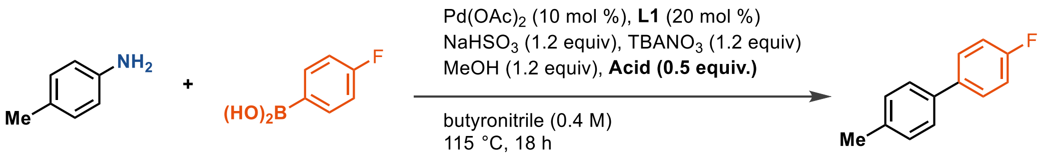

1p (0.2 mmol scale) **2a** (2.2 equiv) **3q**

entry	Acid additive	yield of **3q**/%^{a}
1	$HBF_4 \cdot OEt_2$	43
2	TfOH	38
3	$TsOH \cdot H_2O$	35
4	HNO_3 (aq, 65 %)	40
5	$BF_3 \cdot OEt_2$	30
6	TFA	31
7	MsOH	12
8	$B(OH)_3$ (2.2 equiv.)	36

Fig S2. Screening of the acid additive. ^{a}Yields were determined by ^{19}F NMR spectroscopy using α,α,α-trifluorotoluene as internal standard.

Procedure

To a 4-mL vial, $TBANO_3$ (73 mg, 0.24 mmol, 1.2 equiv.), $NaHSO_3$ (25 mg, 0.24 mmol, 1.2 equiv.), palladium precatalyst (0.20 mmol, 0.10 equiv.), **L1** (18.6 mg, 0.0400 mmol, 0.200 equiv.), *p*-toluidine (**1p**) (18.6 mg, 0.200 mmol, 1.00 equiv.) and **2a** (62 mg, 0.44 mmol, 2.2 equiv.) were added, followed by anhydrous butyronitrile (0.50 mL, c = 0.40 M) and MeOH (9.7 µL, 7.7 mg, 0.24 mmol, 1.2 equiv.). The suspension was stirred for 5 minutes at 23 °C and then acid additive (0.10 mmol, 0.50 equiv.) was added. The vial was sealed with a septum cap and the reaction mixture was heated at 115 °C and stirred (400 rpm) for 18 h. The reaction

mixture was cooled to 23 °C and a sat. aq. $NaHCO_3$ (1 mL) solution was added, and the resulting mixture was extracted by EtOAc (3 × 2 mL). The combined organic phases were passed through a pad of silica gel (2 g), and the solvent was evaporated *in vacuo*. The yield was determined by ^{19}F NMR spectroscopy at 298 K and 471 MHz, by dissolving the crude material in $CDCl_3$ (0.5 mL) and adding α,α,α-trifluorotoluene (8.2 µL, 9.8 mg, 67 µmol) as internal standard. The integration of the peak corresponding to the internal standard at −62.9 ppm (3F, s) was compared to the peak corresponding to **3q** at −116.7 ppm (1F, (tt, J = 8.7, 5.3 Hz)). The correct peak for **3q** was assigned by comparison to literature.[1]

Screening of the ligand

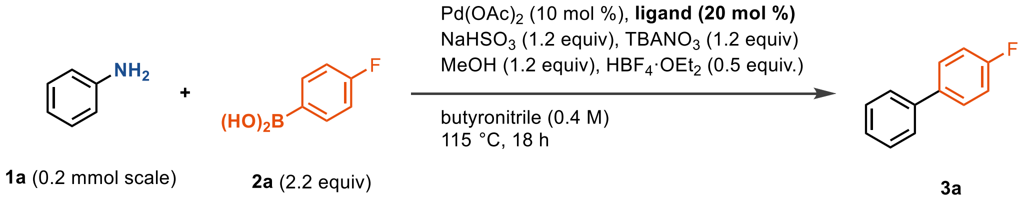

entry	ligand	yield of **3a**/%^{a}
1	**none**	13
2	**L1**	69
3	**L2**	31
4	**L3**	6
5	**L4**	25
6	**L5**	40
7	**L6**	27

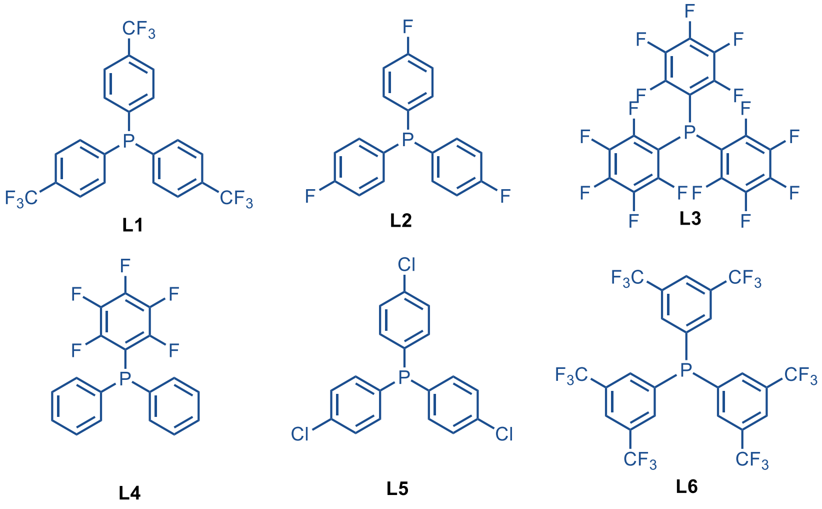

Fig S3. Screening of the ligand. ^{a}Yields were determined by ^{19}F NMR spectroscopy using α,α,α-trifluorotoluene as internal standard.

Procedure

To a 4-mL vial, $TBANO_3$ (73 mg, 0.24 mmol, 1.2 equiv.), $NaHSO_3$ (25 mg, 0.24 mmol, 1.2 equiv.), $Pd(OAc)_2$

(4.6 mg, 0.020 mmol, 0.10 equiv.), ligand (0.040 mmol, 0.20 equiv.) and **2a** (62 mg, 0.44 mmol, 2.2 equiv.) were added, followed by anhydrous butyronitrile (0.50 mL, c = 0.40 M), aniline (18.0 µL, 18.6 mg, 0.200 mmol, 1.00 equiv.) and MeOH (9.7 µL, 7.7 mg, 0.24 mmol, 1.2 equiv.). The suspension was stirred for 5 minutes at 23 °C and then $HBF_4 \cdot OEt_2$ (14 µL, 16 mg, 0.10 mmol, 0.50 equiv.) was added. The vial was sealed with a septum cap and the reaction mixture was heated at 115 °C and stirred (400 rpm) for 18 h. The reaction mixture was cooled to 23 °C and a sat. aq. $NaHCO_3$ (1 mL) solution was added, and the resulting mixture was extracted by EtOAc (3 × 2 mL). The combined organic phases were passed through a pad of silica gel (2 g), and the solvent was evaporated *in vacuo*. The yield was determined by ^{19}F NMR spectroscopy at 298 K and 471 MHz, by dissolving the crude material in $CDCl_3$ (0.5 mL) and adding α,α,α-trifluorotoluene (8.2 µL, 9.8 mg, 67 µmol) as internal standard. The integration of the peak corresponding to the internal standard at −62.9 ppm (3F, s) was compared to the peak corresponding to 3a at −116.2 ppm (1F, (tt, J = 8.7, 5.3 Hz)). The correct peak for **3a** was assigned by comparison to a genuine sample.

Influence of reductant

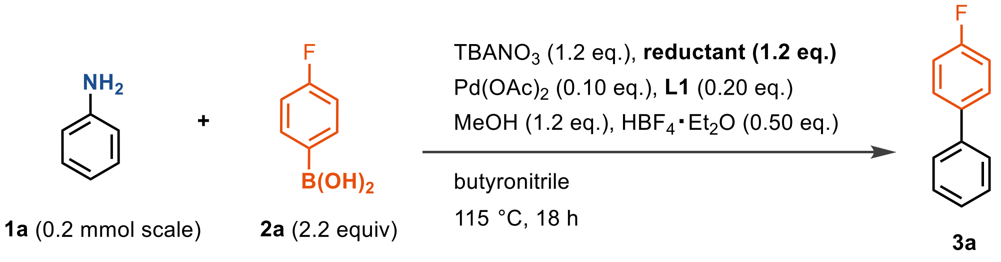

entry	Reductant	yield of **3a**/%
1	$NaHSO_3$	69
2	$Na_2S_2O_3 \cdot 5H_2O$	0
3	$K_2S_4O_6$	0
4	Na_2SO_3	6

Fig S4. Screening of reductants. Yields were determined by ^{19}F NMR spectroscopy using α,α,α-trifluorotoluene as internal standard.

Procedure

To a 4-mL vial, $TBANO_3$ (73 mg, 0.24 mmol, 1.2 equiv.), $NaHSO_3$ (25 mg, 0.24 mmol, 1.2 equiv.), $Pd(OAc)_2$ (4.6 mg, 0.020 mmol, 0.10 equiv.), **L1** (19 mg, 0.040 mmol, 0.20 equiv.) and (4-fluorophenyl)boronic acid (**2a**) (62 mg, 0.44 mmol, 2.2 equiv.) were added followed by anhydrous butyronitrile (0.50 mL, c = 0.40 M), MeOH (24 µL, 19 mg, 0.24 mmol, 1.2 equiv.) and aniline (**1a**) (18.0 µL, 18.6 mg, 0.200 mmol, 1.00 equiv.). The suspension was stirred for 5 minutes at 23 °C and then $HBF_4 \cdot OEt_2$ (14 µL, 16 mg, 0.10 mmol, 0.50 equiv.) was added. The vial was sealed with a septum cap and the reaction mixture was heated at 115 °C and stirred (400 rpm) for 18 h. The reaction mixture was cooled to 23 °C and a sat. aq. $NaHCO_3$ (1 mL) solution was added, and the resulting mixture was extracted by EtOAc (3 × 2 mL). The combined organic phases were

passed through a pad of silica gel (2 g) and the solvent was evaporated *in vacuo*. The yield was determined by ^{19}F NMR spectroscopy at 298 K and 471 MHz, by dissolving the crude material in $CDCl_3$ (0.5 mL) and adding α,α,α-trifluorotoluene (8.2 µL, 9.8 mg, 67 µmol) as internal standard. The integration of the peak corresponding to the internal standard at −62.9 ppm (3F, s) was compared to the peak corresponding to **3a** at −116.2 ppm (1F, (tt, *J* = 8.7, 5.3 Hz)). The correct peak for **3a** was assigned by comparison to a genuine sample.

Influence of reaction temperature

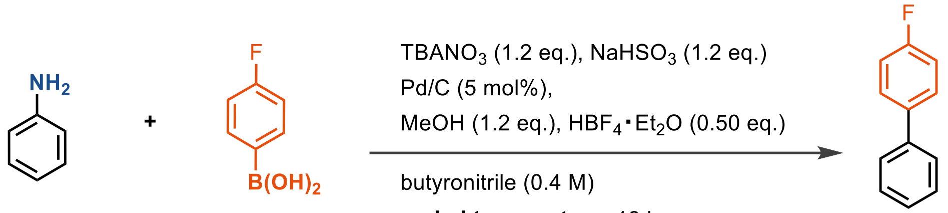

1a (0.2 mmol scale) **2a** (2.2 equiv) **3a**

entry	Temperature	yield of **3a**/%
1	115 °C	28
2	105 °C	17
3	95 °C	16
4	85 °C	3
5	75 °C	1

Fig S5. Screening of reaction temperature. Yields were determined by ^{19}F NMR spectroscopy using α,α,α-trifluorotoluene as internal standard.

Procedure

To a 4-mL vial, $TBANO_3$ (73 mg, 0.24 mmol, 1.2 equiv.), $NaHSO_3$ (25 mg, 0.24 mmol, 1.2 equiv.), Pd/C (10 w/w%, 11 mg, 0.010 mmol, 5.0 mol%), and (4-fluorophenyl)boronic acid (**2a**) (62 mg, 0.44 mmol, 2.2 equiv.) were added followed by anhydrous butyronitrile (0.50 mL), MeOH (24 µL, 19 mg, 0.24 mmol, 1.2 equiv.) and aniline (**1a**) (18.0 µL, 18.6 mg, 0.200 mmol, 1.00 equiv.). The suspension was stirred for 5 minutes at 23 °C and then $HBF_4{\cdot}OEt_2$ (14 µL, 16 mg, 0.10 mmol, 0.50 equiv.) was added. The vial was sealed with a septum cap and the reaction mixture was heated at the appropriate temperature and stirred (400 rpm) for 18 h. The reaction mixture was cooled to 23 °C and a sat. aq. $NaHCO_3$ (1 mL) solution was added, and the resulting mixture was extracted by EtOAc (3 × 2 mL). The combined organic phases were passed through a pad of silica gel (2 g) and the solvent was evaporated *in vacuo*. The yield was determined by ^{19}F NMR spectroscopy at 298 K and 471 MHz, by dissolving the crude material in $CDCl_3$ (0.5 mL) and adding α,α,α-trifluorotoluene (8.2 µL, 9.8 mg, 0.067 mmol) as internal standard. The integration of the peak corresponding to the internal standard at −62.9 ppm (3F, s) was compared to the peak corresponding to **3a** at −116.2 ppm (1F, (tt, *J* = 8.7, 5.3 Hz)). The correct peak for **3a** was assigned by comparison to a genuine sample.

Control experiments

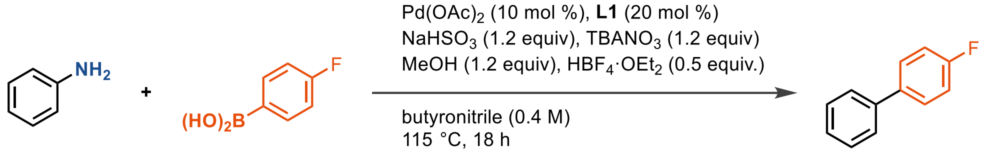

1a (0.2 mmol scale) **2a** (2.2 equiv) **3a**

entry	Changes from conditions	yield of **3a**/%[a]
1	Without $HBF_4 \cdot Et_2O$	36
2	Without **L1**	13
3	$Pd(dba)_2$ instead of $Pd(OAc)_2$	65
4	PPh_3 instead of **L1**	28
5	Without $Pd(OAc)_2$	0

Fig S6. Control experiments. [a]Yields were determined by ^{19}F NMR spectroscopy using α,α,α-trifluorotoluene as internal standard.

Procedure

To a 4-mL vial, $TBANO_3$ (73 mg, 0.24 mmol, 1.2 equiv.), $NaHSO_3$ (25 mg, 0.24 mmol, 1.2 equiv.), palladium precatalyst (0.020 mmol, 0.10 equiv.), ligand (0.040 mmol, 0.20 equiv.) and **2a** (62 mg, 0.44 mmol, 2.2 equiv.) were added, followed by anhydrous butyronitrile (0.50 mL, c = 0.40 M), aniline (**1a**) (18.0 µL, 18.6 mg, 0.200 mmol, 1.00 equiv.) and MeOH (9.7 µL, 7.7 mg, 0.24 mmol, 1.2 equiv.). The suspension was stirred for 5 minutes at 23 °C and then where applicable $HBF_4 \cdot OEt_2$ (14 µL, 16 mg, 0.24 mmol, 0.5 equiv.) was added. The vial was sealed with a septum cap and the reaction mixture was heated at 115 °C and stirred (400 rpm) for 18 h. The reaction mixture was cooled to 23 °C and a sat. aq. $NaHCO_3$ (1 mL) solution was added, and the resulting mixture was extracted by EtOAc (3 × 2 mL). The combined organic phases were passed through a pad of silica gel (2 g), and the solvent was evaporated *in vacuo*. The yield was determined by ^{19}F NMR spectroscopy at 298 K and 471 MHz, by dissolving the crude material in $CDCl_3$ (0.5 mL) and adding α,α,α-trifluorotoluene (8.2 µL, 9.8 mg, 67 µmol) as internal standard. The integration of the peak corresponding to the internal standard at −62.9 ppm (3F, s) was compared to the peak corresponding to **3a** at −116.2 ppm (1F, (tt, J = 8.7, 5.3 Hz)). The correct peak for **3a** was assigned by comparison to a genuine sample.

Influence of nucleophile species on the reaction

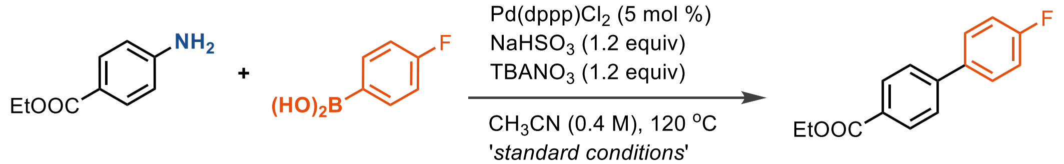

1b (0.2 mmol scale) **2a** (3.5 equiv.) **3b**

entry	change from the standard conditions	conv. of **1a**/%	yield of **3b**/%
1	no	83	55
2	**2a** (2.5 equiv)	75	42
3	**9** instead of **2a**	0	0
4	**10** instead of **2a**	0	0
5	**2r** instead of **2a**	51	25 (**3r**)
6	1.0 equiv $BF_3 \cdot OEt_2$	>99	46

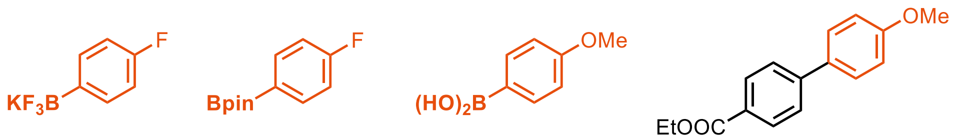

9 **10** **2r** **3r**

Fig S7. Screening of the phenylboronic acids. Yields were determined by ^{1}H NMR spectroscopy using 1,3,5-trimethoxybenzene as internal standard.

Procedure

To a 4-mL vial, $TBANO_3$ (73 mg, 0.24 mmol, 1.2 equiv.), $NaHSO_3$ (25 mg, 0.24 mmol, 1.2 equiv.), Pd catalyst (0.020 mmol, 0.10 equiv.), nucleophile species (0.70 mmol, 3.5 equiv.) and benzocaine (**1b**) (33.0 mg, 0.200 mmol, 1.00 equiv.) were added followed by anhydrous solvent (0.50 mL, c = 0.40 M) and additive. The vial was sealed with a septum cap and the reaction mixture was heated at 115 °C and stirred (400 rpm) for 18 h. The reaction mixture was cooled to 23 °C and 1,3,5-trimethoxybenzene (11.2 mg, 0.067 mmol, 0.33 equiv.) was added. A glass pipette was used to pick up one drop of the mixture and it was filtered by a thin layer of Celite and washed into an NMR tube with $CDCl_3$ (0.5 mL). Yields and conversions were determined by ^{1}H NMR using 1,3,5-trimethoxybenzene as internal standard. The integration of the peak corresponding to the internal standard at 6.13 ppm (3H, s) was compared to the peak corresponding to **3b** at 8.15 –8.09 ppm (2H, m) and to the peak corresponding to **1b** at 7.06 ppm (d, *J* = 8.4 Hz, 2H). The correct peak for **3b** and **1b** was assigned by comparison to a genuine sample.

Reactions with other cross-coupling reagents

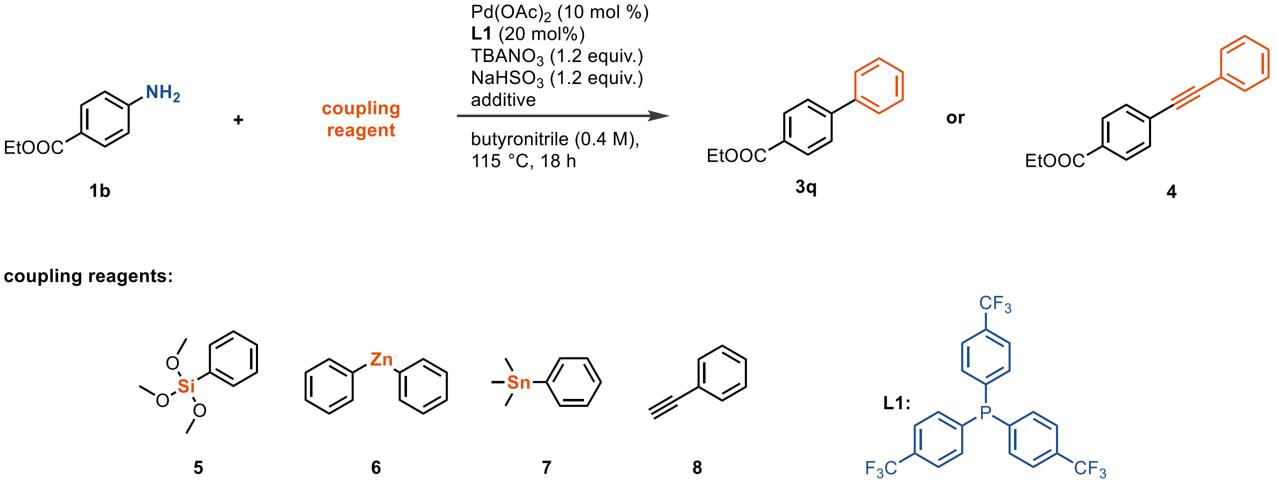

Yield of **3q** or **4**: <1 % (**3q**) <1 % (**3q**) <1 % (**3q**) 14 % (**4**)

Fig S8. Reactions with other cross-coupling reagents

Hiyama-coupling

To a 4-mL vial, $TBANO_3$ (73 mg, 0.24 mmol, 1.2 equiv.), $NaHSO_3$ (25 mg, 0.24 mmol, 1.2 equiv.), $Pd(OAc)_2$ (4.6 mg, 0.020 mmol, 0.10 equiv.) **L1** (19 mg, 0.040 mmol, 0.20 equiv.), benzocaine (**1b**) (33 mg, 0.20 mmol, 1.0 equiv.) and tetrabutylammonium fluoride trihydrate (139 mg, 0.440 mmol, 2.20 equiv.) were added. The vial was sealed with a septum cap and the atmosphere was replaced with argon. Anhydrous butyronitrile (0.50 mL, c = 0.40 M), MeOH (10 µL, 7.9 mg, 0.24 mmol, 1.2 equiv.) and phenyl trimethylsiloxane (**5**) (82 µL, 87 mg, 0.44 mmol, 2.2 equiv.) were added and the reaction mixture was heated at 115 °C and stirred (400 rpm) for 18 h. The reaction mixture was cooled to 23 °C and diluted with DCM (2 mL). A sat. aq. NH_4Cl solution (3 mL) was added, and the layers were separated. The aqueous layer was washed with DCM (2 × 2 mL). The combined organic phases were dried over $MgSO_4$, filtered and the filtrate was evaporated *in vacuo*. The crude material was diluted with cyclohexane:EtOAc (3:1, 5 mL) and passed through a short pad of silica. The resulting solution was evaporated *in vacuo*. The remaining crude material was dissolved in $CDCl_3$ (0.5 mL) and a ^1H NMR measurement (298 K, 500 MHz) was performed. The peaks of the literature known compound,[2] **3q** were absent in the resulting spectrum, concluding that the coupling reaction failed.

Negishi-coupling

To a 4-mL vial, $TBANO_3$ (73 mg, 0.24 mmol, 1.2 equiv.), $NaHSO_3$ (25 mg, 0.24 mmol, 1.2 equiv.), $Pd(OAc)_2$ (4.6 mg, 0.020 mmol, 0.10 equiv.) **L1** (19 mg, 0.040 mmol, 0.20 equiv.), benzocaine (**1b**) (33 mg, 0.20 mmol, 1.0 equiv.) and diphenylzinc (**6**) (48 mg, 0.22 mmol, 1.1 equiv.) were added. The vial was sealed with a septum cap and the atmosphere was replaced with argon. Anhydrous butyronitrile (0.50 mL, c = 0.40 M) and MeOH (10 µL, 7.9 mg, 0.24 mmol, 1.2 equiv.) were added and the reaction mixture was heated at 115 °C and stirred (400 rpm) for 18 h. The reaction mixture was cooled to 23 °C and diluted with DCM (2 mL). A sat. aq. NH_4Cl solution (3 mL) was added and the layers were separated. The aqueous layer was washed with DCM

(2 × 2 mL). The combined organic phases were dried over $MgSO_4$, filtered and the filtrate was evaporated *in vacuo*. The remaining crude material was dissolved in $CDCl_3$ (0.5 mL) and a ^1H NMR measurement (298 K, 500 MHz) was performed. The peaks of the literature known compound,[2] **3q** were absent in the resulting spectrum, concluding that the coupling reaction failed.

Stille-coupling

To a 4-mL vial, $TBANO_3$ (73 mg, 0.24 mmol, 1.2 equiv.), $NaHSO_3$ (25 mg, 0.24 mmol, 1.2 equiv.), $Pd(OAc)_2$ (4.6 mg, 0.020 mmol, 0.10 equiv.) **L1** (19 mg, 0.040 mmol, 0.20 equiv.) and benzocaine (**1b**) (33 mg, 0.20 mmol, 1.0 equiv.) were added. The vial was sealed with a septum cap and the atmosphere was replaced with argon. Anhydrous butyronitrile (0.50 mL, c = 0.40 M), MeOH (10 µL, 7.9 mg, 0.24 mmol, 1.2 equiv.) and trimethyl(phenyl)tin (**7**) (80 µL, 0.10 g, 0.44 mmol, 2.2 equiv.) were added and the reaction mixture was heated at 115 °C and stirred (400 rpm) for 18 h. The reaction mixture was cooled to 23 °C and diluted with DCM (2 mL). Water (3 mL) was added, and the layers were separated. The organic layer was washed with aq. KF solution (1 M, 2 × 3 mL) and brine (3 mL). The organic phase was dried over $MgSO_4$, filtered and the filtrate was evaporated *in vacuo*. The crude material was diluted with cyclohexane:EtOAc (3:1, 5 mL) and passed through a short pad of silica. The resulting solution was evaporated *in vacuo*. The remaining crude material was dissolved in $CDCl_3$ (0.5 mL) and a ^1H NMR measurement (298 K, 500 MHz) was performed. The peaks of the literature known compound,[2] **3q** were absent in the resulting spectrum, concluding that the coupling reaction failed.

Sonogashira-coupling

To a 4-mL vial, $TBANO_3$ (73 mg, 0.24 mmol, 1.2 equiv.), $NaHSO_3$ (25 mg, 0.24 mmol, 1.2 equiv.), $Pd(OAc)_2$ (4.6 mg, 0.020 mmol, 0.10 equiv.) **L1** (19 mg, 0.040 mmol, 0.20 equiv.), benzocaine (**1b**) (33 mg, 0.20 mmol, 1.0 equiv.) and copper(I) iodide (3.8 mg, 0.020 mmol, 0.10 equiv.) were added. The vial was sealed with a septum cap and the atmosphere was replaced with argon. Anhydrous butyronitrile (0.50 mL, c = 0.40 M), MeOH (10 µL, 7.9 mg, 0.24 mmol, 1.2 equiv.), phenylacetylene (**8**) (48 µL, 44 mg, 0.44 mmol, 2.2 equiv.) and $HBF_4{\cdot}OEt_2$ (14 µL, 16 mg, 0.10 mmol, 0.5 equiv.) were added, and the reaction mixture was heated at 115 °C and stirred (400 rpm) for 18 h. The reaction mixture was cooled to 23 °C and $NaHCO_3$ (25 mg, 0.30 mmol, 1.5 equiv.) was added. The mixture was diluted with cyclohexane:EtOAc (3:1, 5 mL) and passed through a short pad of silica. The resulting solution was evaporated *in vacuo*. The yield was determined by ^1H NMR spectroscopy at 298 K and 500 MHz, by dissolving the crude material in $CDCl_3$ (0.5 mL) and adding CH_2Br_2 (14.0 µL, 35.0 mg, 0.200 mmol) as internal standard. The integration of the peak corresponding to the internal standard at 4.87 ppm (2H, s) was compared to the peak corresponding to **4** at 7.98 ppm (2H, m) and at 7.54 ppm (2H, m). The correct peak for **4** was assigned by comparison to literature.[3]

Mechanistic studies

NO_2 and SO_2 detection experiments

A. NO_2 detection experiment

$$NaHSO_3 + TBANO_3 \xrightarrow[\text{butyronitrile, rt to 115 °C}]{\text{Additive}} NO_2$$

entry	Additive	NO_2 detection T (°C)
1	no	not detected (A)
2	**2a**	105 (A)
3	HBF_4·Et2O	50 (B)
4	**1a**	not detected (A)
5	MeOH	not detected (A)

B. SO_2 detection experiment

$$NaHSO_3 \xrightarrow[\text{butyronitrile, rt to 115 °C}]{\text{Additive}} SO_2$$

entry	Additive	SO_2 detection T (°C)
6	no	100
7	**2a**	30
8	HBF_4·Et2O	80
9	**1a**	100
10	HNO_3	30

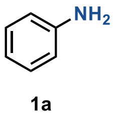

1a

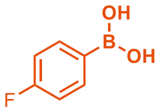

2a

Fig S9. NO_2 and SO_2 detection experiments.

NO_2 detection experiment procedure.

Method A: To a 4-mL vial, $TBANO_3$ (73 mg, 0.24 mmol, 1.0 equiv.), $NaHSO_3$ (25 mg, 0.24 mmol, 1.0 equiv.) were added followed by anhydrous butyronitrile (0.50 mL, c = 0.48 M) and additive (1.0 equiv., 0.24 mmol). The vial was sealed with a septum cap and the reaction mixture was heated at 30 °C and stirred (400 rpm) for 5 minutes. The temperature was further increased by 10 °C increments up to 115 °C with 5 minutes of stirring in between each increment. NO_2 was detected as a brown gas in the headspace of the reaction vessel. The temperature at which this gas was first detected was noted down.

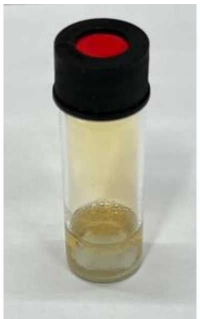

Method B: To a 4-mL vial, $TBANO_3$ (73 mg, 0.24 mmol, 1.0 equiv.), $NaHSO_3$ (25 mg, 0.24 mmol, 1.0 equiv.) were added followed by anhydrous butyronitrile (0.50 mL, c = 0.48 M) and additive (1.0 equiv., 0.24 mmol). The vial was sealed with a septum cap and the reaction mixture was heated at 115 °C while stirring (400 rpm). NO_2 was detected as a brown gas in the headspace of the reaction vessel. The temperature at which this gas was first detected was noted down. The brown headspace of the 4-mL vial was taken up using a 5 mL plastic syringe with a metal needle. A closable quartz cuvette was filled with 2 mL of MeCN and subsequently the contents of the syringe were bubbled through the MeCN. The cuvette was closed and a UV-Vis spectrum recorded. A reference spectrum of NO_2 was recorded by bubbling NO_2 through a cuvette containing MeCN.

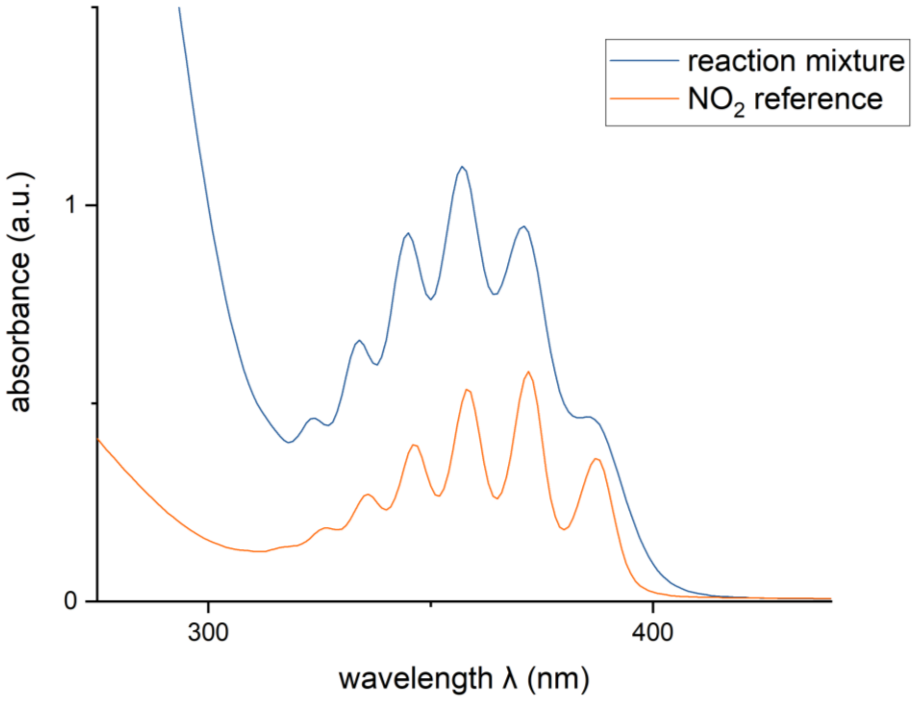

Fig S10. Stacked UV-Vis spectra at 23 °C in MeCN of reaction mixture headspace with reference spectrum of NO_2.

Note

Our previous study showed that NO_3^- reduction to NO^+ will proceed through NO_2 as an intermediate.[4] Thus, we performed the above experiments to confirm the presence of NO_2. NO_2 can be visually detected as a brown gas in the headspace. We confirmed the presence of NO_2 in the brown headspace via UV-Vis spectroscopy (Fig. S10). In the gas phase IR measurement of the headspace of the reaction NO_2 is not detected, (Fig. S11) most likely due to its instability and low concentration. The more stable N_2O species is detected which is known to form in the nitrate reduction pathway.[5] The detection of N_2O is an additional indication for a nitrate reduction process.

IR measurement of the reaction headspace

To understand the composition of the headspace of the above experiment a gas IR measurement was performed. To a 4-mL vial, $TBANO_3$ (73 mg, 0.24 mmol, 1.0 equiv.), $NaHSO_3$ (25 mg, 0.24 mmol, 1.0 equiv.) were added followed by anhydrous butyronitrile (0.50 mL, c = 0.40 M) and $HBF_4 \cdot OEt_2$ (14 µL, 16 mg, 0.10 mmol, 0.50 equiv.). The vial was sealed with a septum cap and the reaction mixture was heated at 100 °C and stirred (400 rpm) for 5 minutes. After the formation of the brown gas in the headspace a 5 mL syringe was used to take out the headspace gases. This syringe was injected into the IR spectrometer. Reference spectra were measured by direct injection of pure gaseous samples.

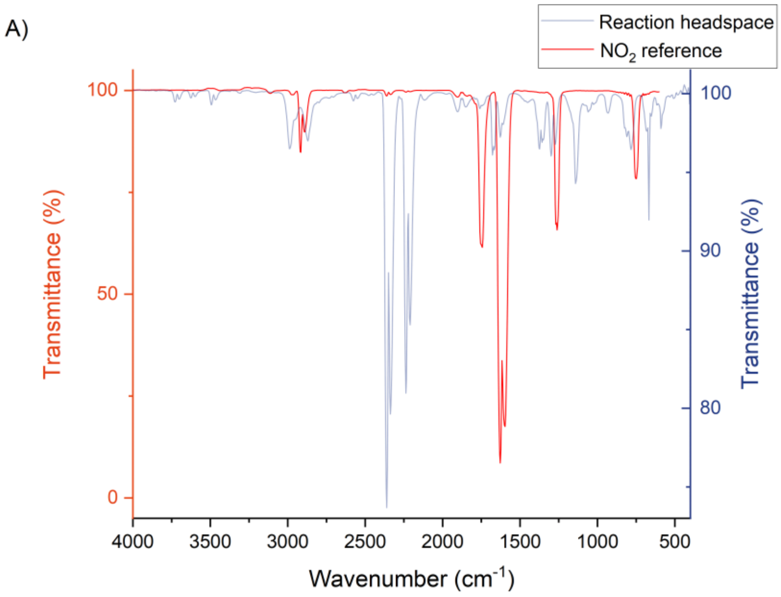

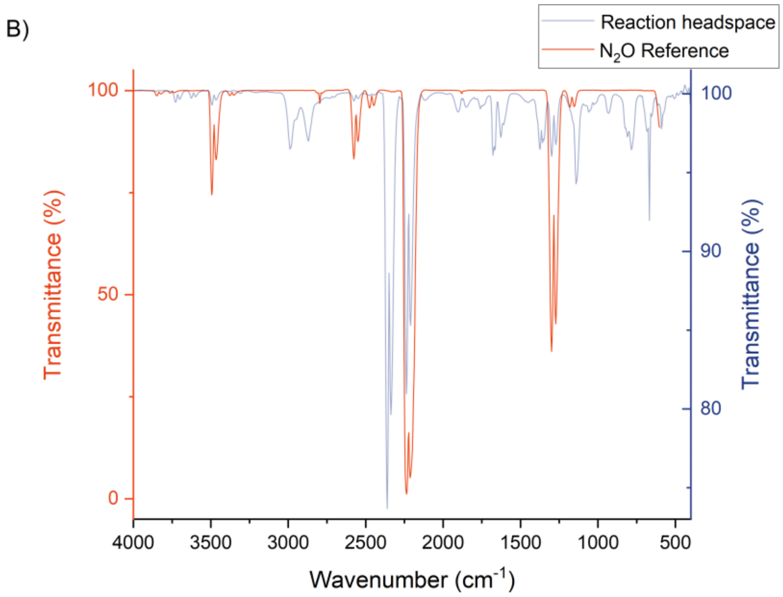

Fig S11. IR spectrum of the reaction headspace (blue) compared with a reference spectrum of NO_2 (orange, top) and with a reference spectrum of N_2O (orange, bottom)

SO_2 detection experiment procedure.

To a 4-mL vial $NaHSO_3$ (25 mg, 0.24 mmol, 1.2 equiv.) was added followed by anhydrous butyronitrile (0.50 mL, c = 0.40 M) and additive (0.24 mmol, 1.0 equiv.). The vial was sealed with a septum cap and the reaction mixture was heated at 30 °C and stirred (400 rpm) for 5 minutes. The vial was opened and a strip of filter paper soaked in aqueous $KMnO_4$ solution was held in the headspace of the reaction. The vial was closed, and the following steps were repeated: the temperature was increased by 10 °C, the reaction was stirred (400 rpm) for 5 minutes, the mixture was cooled to 23 °C and a strip of filter paper soaked in aqueous $KMnO_4$ solution was held in the headspace of the reaction. This was repeated until 115 °C was reached. SO_2 detection was noted down at the lowest temperature at which the color of the filter paper turned from purple to brown or colorless.

Preparation of diazonium salt (10a)

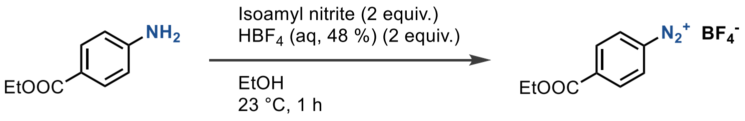

1b (0.2 mmol) **10a** (88 %)

In a 4-mL vial, benzocaine (**1b**) (165 mg, 1.00 mmol, 1.00 equiv.) was dissolved in ethanol (1.25 mL, c = 0.8 M) followed by the addition of an aqueous HBF_4 solution (48 %, 0.26 mL, 0.36 g, 2.0 mmol, 2.0 equiv.). The solution was cooled to -20 °C and isoamyl nitrite (0.27 mL, 0.23 g, 2.0 mmol, 2.0 equiv.) was added dropwise. The reaction mixture was warmed up to 23 °C and stirred for 1 hour. The resulting suspension was diluted with Et_2O (2 mL), filtered and washed with additional Et_2O (30 mL). The obtained salt was dried under high vacuum to afford 231 mg **10a** as a white solid (88 %).

NMR Spectroscopy:

^{1}H NMR (500 MHz, MeCN-d_3, 23 °C): δ = 8.62 (dm, J = 9.0 Hz, 1H), 8.41 (dm, J = 9.0 Hz, 0H), 4.43 (q, J = 7.1 Hz, 1H), 1.39 (t, J = 7.2 Hz, 1H) ppm.

^{13}C NMR (125 MHz, MeCN-d_3, 23 °C): δ = 164.1, 142.3, 133.7, 132.6, 119.3, 63.7, 14.0 ppm.

^{19}F NMR (471 MHz, MeCN-d_3, 23 °C): δ = −150.9 ppm.

HRMS (ESI): calc'd for $C_9H_9O_2N_2$ $[M]^+$: 177.0659, found: 177.0659; deviation: +0.2 ppm.

NO$^+$ competition experiments

Competition between *diazotization* vs *oxidation of Pd*

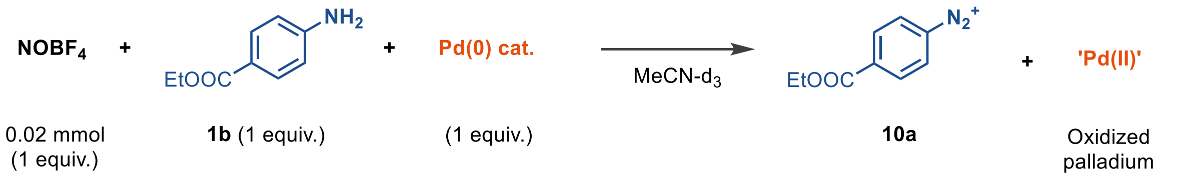

entry	Pd(0) cat.	yield of **10a**/%
1 (control)	-	83
2	$Pd(dba)_2$ (1 equiv.)	42
3	$Pd(dba)_2$ (1 equiv.) **L1** (1 equiv.)	35

Competition between *oxidative addition to **10a*** vs *oxidation of Pd*

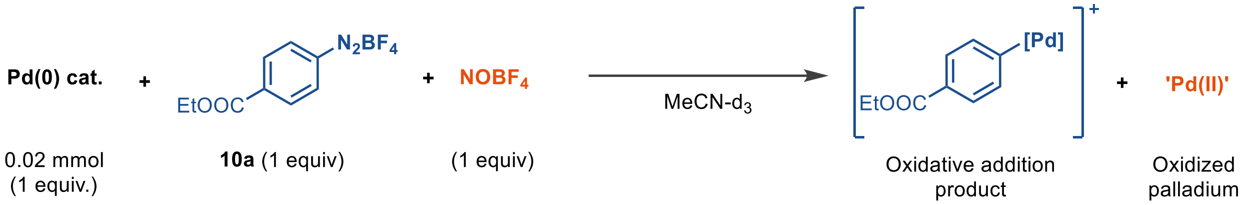

entry	Pd(0) cat.	conversion of **10a**/%
1 (control)	-	<1
2	$Pd(dba)_2$ (1 equiv.)	7
3	$Pd(dba)_2$ (1 equiv.) **L1** (1 equiv.)	11

Fig S12. NO$^+$ competition experiments. Yields and conversions were determined by ^{1}H NMR spectroscopy using diphenylmethane as internal standard.

Procedure

To a flame dried 4-mL vial, benzocaine (**1b**) (3.3 mg, 20 µmol, 1.0 equiv.) or benzocaine diazonium salt (**10a**) (5.3 mg, 20 µmol, 1.0 equiv.) was dissolved in MeCN-d_3 (0.5 mL) under Ar atmosphere. Where applicable additives (20 µmol, 1.0 equiv.) were dissolved in MeCN-d_3 in another flame dried 4-mL vial under Ar atmosphere. In the cases where $Pd(dba)_2$ and **L1** were both used as additives, the resulting suspension was stirred at 23 °C for 15 minutes. Another flame dried 4-mL vial was charged with $NOBF_4$ (2.3 mg, 20 µmol, 1.0 equiv.) inside a glovebox. This vial was taken outside the glovebox and all of the previously made solutions were simultaneously added to the vial through a septum. To the resulting solution diphenylmethane (3.3 µL, 3.3 mg, 20 µmol, 1.0 equiv.) was added as an internal standard. The yields and conversions of **10a** were determined by ^{1}H NMR spectroscopy at 298 K and 500 MHz, using diphenylmethane as internal

standard. The integration of the peak corresponding to the internal standard at 3.96 ppm (2H, s) was compared to the peaks corresponding to **10a** at 8.58 (dm, *J* = 8.9 Hz, 1H) ppm and 8.42 (dm, *J* = 8.8 Hz, 1H) ppm. The correct peak for **10b** was assigned by comparison to a genuine sample.

Analysis of ligand decomposition

Detection of decomposition products

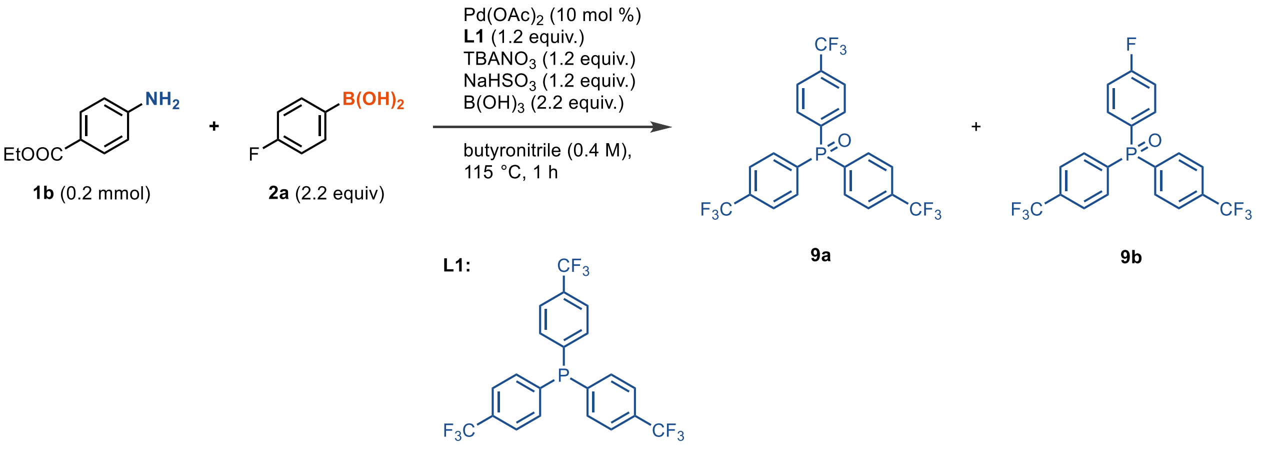

Fig S13. Decomposition of ligand **L1**.

Procedure

To a 4-mL vial, $TBANO_3$ (73 mg, 0.24 mmol, 1.2 equiv.), $NaHSO_3$ (25 mg, 0.24 mmol, 1.2 equiv.), $Pd(OAc)_2$ (4.5 mg, 0.020 mmol, 0.10 equiv.), **2a** (61.6 mg, 0.44 mmol, 2.2 equiv.) and benzocaine (**1b**) (33.0 mg, 0.200 mmol, 1.00 equiv.) were added followed by anhydrous butyronitrile (0.50 mL, c = 0.40 M). The suspension was stirred for 5 minutes at 23 °C and then $HBF_4·OEt_2$ (14 µL, 16 mg, 0.10 mmol, 0.50 equiv.) was added. The vial was sealed with a septum cap and the reaction mixture was heated at 115 °C and stirred (400 rpm) for 18 h. The reaction mixture was cooled to 23 °C and a sat. aq. $NaHCO_3$ (1 mL) solution was added, and the resulting mixture was extracted by EtOAc (3 × 2 mL). The combined organic phases were passed through a pad of silica gel (2 g) and the solvent was evaporated *in vacuo*. The crude material was dissolved in $CDCl_3$ (0.5 mL) and ^{31}P NMR and HMRS (ESI) measurements were taken.

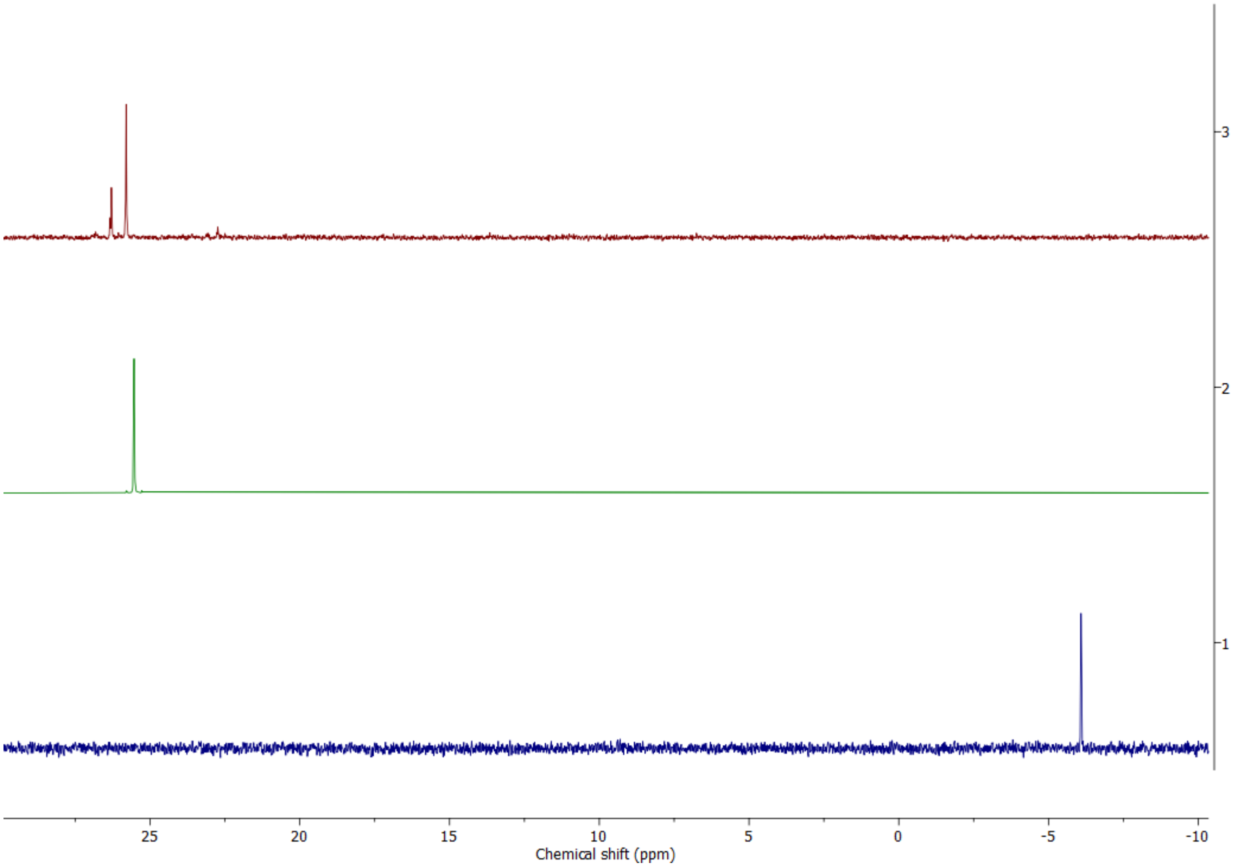

Fig S14. ^{31}P NMR spectra of the crude reaction mixture (top), a sample of pure **9a** (middle) and a sample of pure **L1** (bottom). A small chemical shift region is shown for clarity, with no other peaks present in other regions.

HRMS results

Detection of **9a**: **HRMS (ESI):** calc'd for $C_{21}H_{13}O_1F_9P_1$ $[M]^+$: 483.0555, found: 483.0558; deviation: +0.7 ppm.

Detection of **9b: HRMS (ESI):** calc'd for $C_{20}H_{13}O_1F_7P_1$ $[M]^+$: 433.0587, found: 433.0588; deviation: +0.3 ppm

Kinetics of the formation of 9a

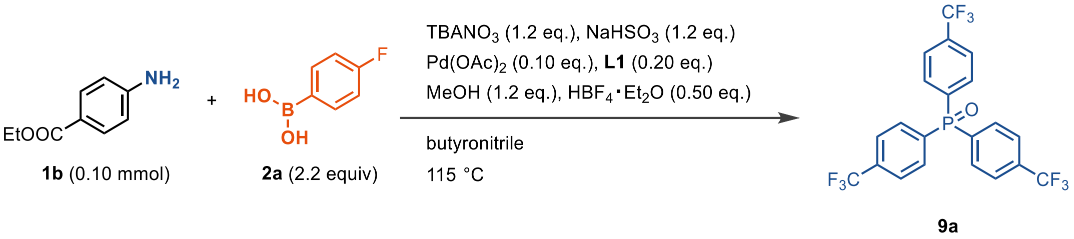

L1:

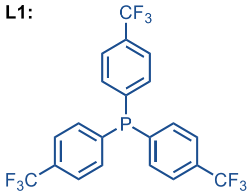

Fig S15. Formation of **9a.**

Procedure

To a 4-mL vial, $TBANO_3$ (36.5 mg, 0.12 mmol, 1.20 equiv.), $NaHSO_3$ (12.5 mg, 0.12 mmol, 1.20 equiv.), $Pd(OAc)_2$ (2.2 mg, 0.010 mmol, 0.10 equiv.), **L1** (9.3 mg, 0.020 mmol, 0.20 equiv.), **2a** (30.8 mg, 0.22 mmol, 2.2 equiv.) and benzocaine (**1b**) (16.5 mg, 0.100 mmol, 1.00 equiv.) were added followed by anhydrous butyronitrile (0.25 mL, c = 0.40 M) and MeOH (4.8 µL, 3.8 mg, 0.12 mmol, 1.2 equiv.). The suspension was stirred for 5 minutes at 23 °C and then $HBF_4{\cdot}OEt_2$ (6.8 µL, 8.1 mg, 0.050 mmol, 0.50 equiv.) was added. The vial was sealed with a septum cap and the reaction mixture was heated at 115 °C and stirred (400 rpm) for a specified time. The reaction mixture was cooled to 23 °C, and MeCN-d_3 (0.3 mL) and triphenyl phosphate (6.5 mg, 0.020 mmol, 0.20 equiv.) was added. The yield of **9a** were determined by ^{31}P NMR spectroscopy at 298 K and 203 MHz, using triphenyl phosphate as internal standard. The integration of the peak corresponding to the internal standard at –17.2 ppm was compared to the peak corresponding to **9a** at 23.9 ppm. The correct peak for **9b** was assigned by comparison to a genuine sample. The yields of **9a** are compared to **L1.**

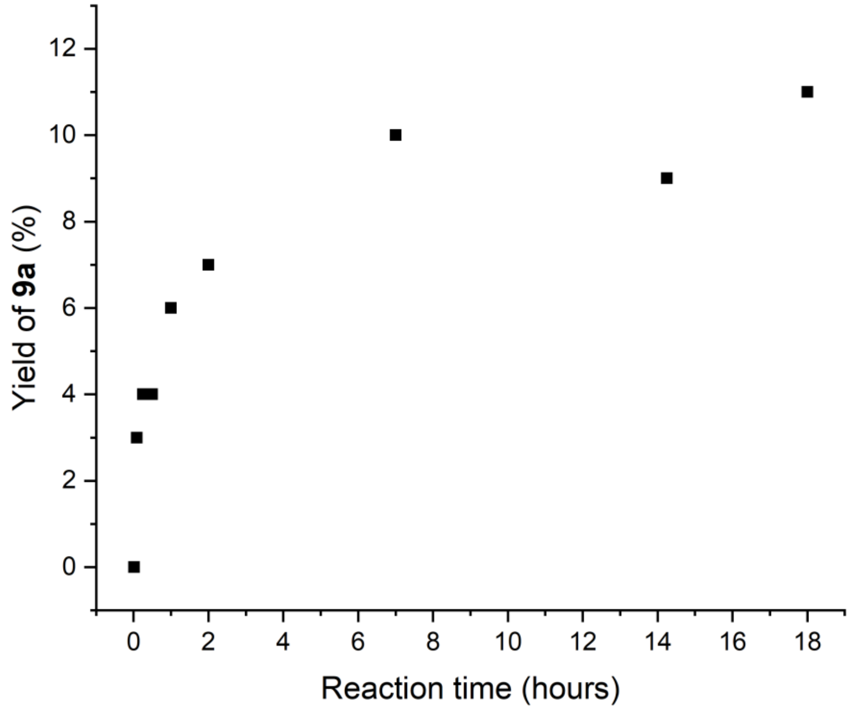

Fig S16. The yield of **9a** (referenced to **L1**) over time. Yields were determined by ^{31}P NMR spectroscopy using triphenyl phosphate as an internal standard.

Analysis of reaction with triphenylphosphine

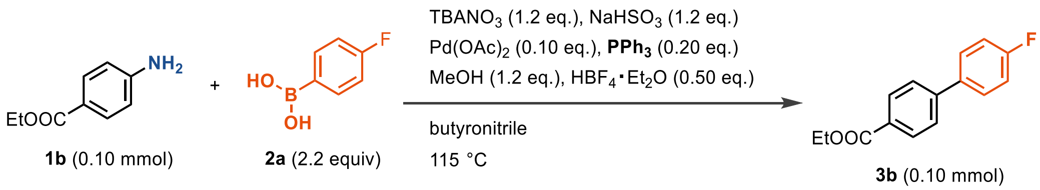

Fig S17. Reaction with triphenyl phosphine as ligand.

Procedure

To a 4-mL vial, $TBANO_3$ (36.5 mg, 0.12 mmol, 1.20 equiv.), $NaHSO_3$ (12.5 mg, 0.12 mmol, 1.20 equiv.), $Pd(OAc)_2$ (2.2 mg, 0.010 mmol, 0.10 equiv.), PPh_3 (5.2 mg, 0.020 mmol, 0.20 equiv.), **2a** (30.8 mg, 0.22 mmol, 2.2 equiv.) and benzocaine (**1b**) (16.5 mg, 0.200 mmol, 1.00 equiv.) were added followed by anhydrous butyronitrile (0.25 mL, c = 0.40 M) and MeOH (4.8 µL, 3.8 mg, 0.12 mmol, 1.2 equiv.). The suspension was stirred for 5 minutes at 23 °C and then $HBF_4{\cdot}OEt_2$ (6.8 µL, 8.1 mg, 0.050 mmol, 0.50 equiv.) was added. The vial was sealed with a septum cap and the reaction mixture was heated at 115 °C and stirred (400 rpm) for a specified time. The reaction mixture was cooled to 23 °C, and MeCN-d_3 (0.3 mL) and triphenyl phosphate (6.5 mg, 0.020 mmol, 0.20 equiv.) was added and the mixture was analyzed with ^{31}P

NMR spectroscopy.

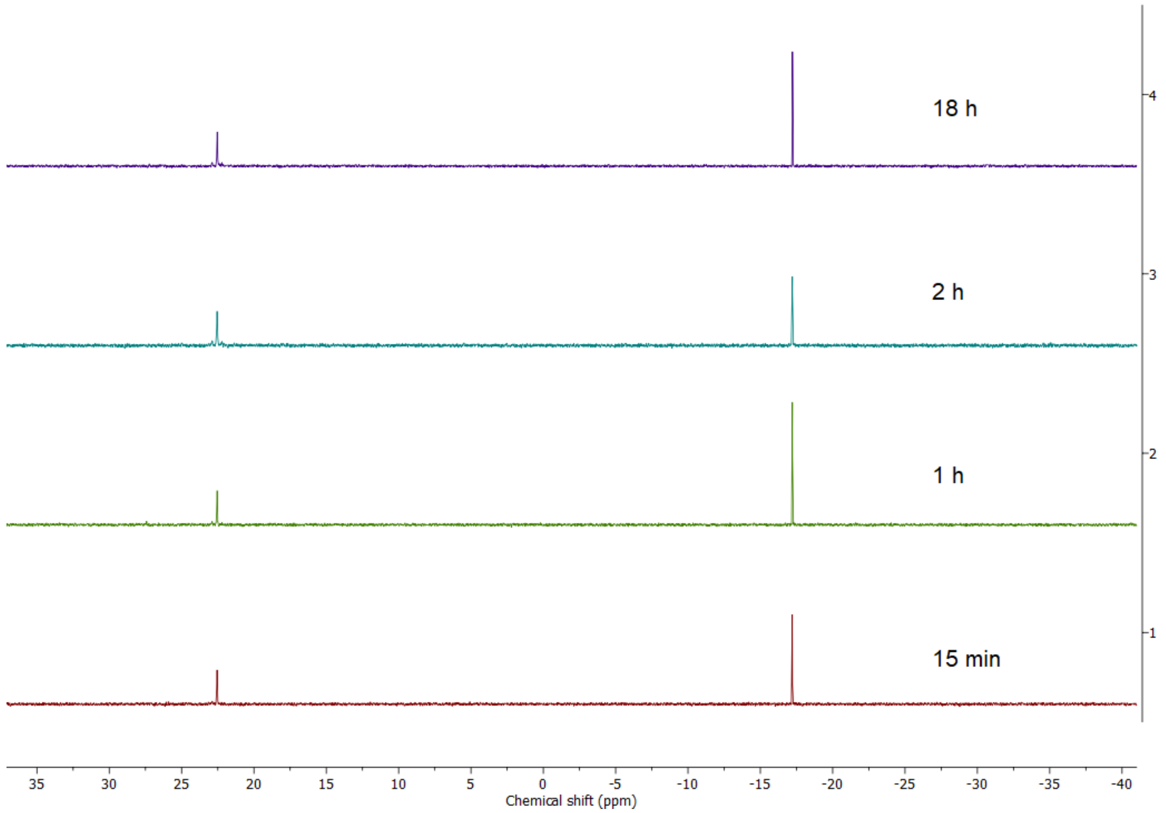

Fig S18. ^{31}P NMR (203 MHz, 300 K) spectra of the reaction with PPh_3 stopped at different reaction times. Not the full chemical shift window is shown for clarity, as no other peaks are present in the full spectra. The peaks at –17.2 ppm correspond to triphenyl phosphate as the internal standard. In all four spectra, the integral ratios of the peaks at –17.2 ppm and 22.5 ppm are 1:1.

Note

The result show that a phosphorus species forms early in the reaction and stays there for the whole duration of the reaction. The integral ratio of this newly appeared peak and internal standard is 1:1 at all times showing full conversion of PPh_3 into the new phosphorus species. Further analysis was performed to identify the unknown phosphorus species.

Identification attempts of the formed phosphorus species

To determine the identity of the formed phosphorus species the following comparison measurements were taken:

1) To the NMR sample prepared from the reaction mixture with a reaction time of 18 hours was added a genuine sample of PPh_3 and $O{=}PPh_3$.
2) $Pd(OAc)_2$ (2.2 mg, 0.010 mmol, 1.0 equiv.) and PPh_3 (5.2 mg, 0.020 mmol, 2.0 equiv.), was stirred in $MeCN\text{-}d_3$ (0.5 mL), for 15 minutes at 60 °C.

3) Diazonium salt **10a** (5.3 mg, 0.010 mmol, 1.0 equiv.) and PPh_3 (5.2 mg, 0.010 mmol, 1.0 equiv.) was dissolved in MeCN-d_3 (0.5 mL). A color change from colorless to red was observed.

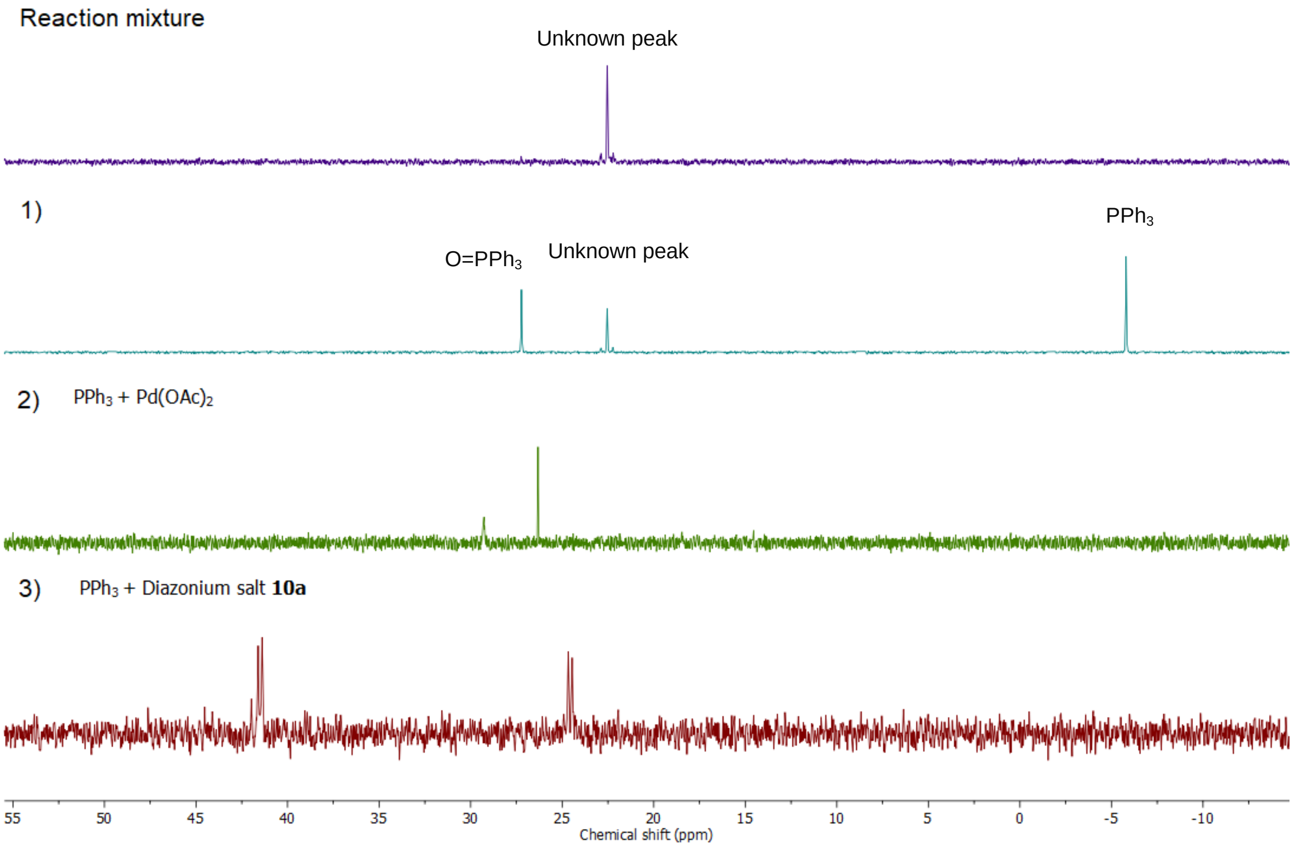

Fig S19. ^{31}P NMR spectra (203 MHz, 300 K) of the reaction mixture with reaction time 18 h (top), and comparison mixtures 1)–3).

Preparation of phosphine oxide 9a

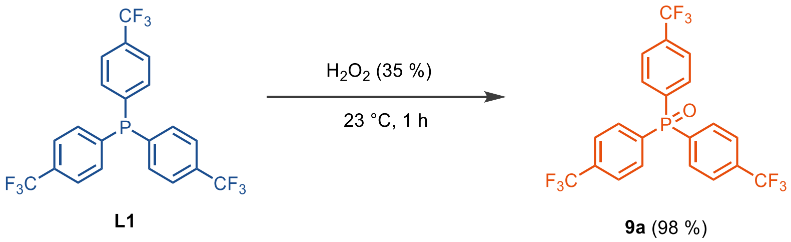

A 4-mL vial was charged with **L1** (100 mg, 0.214 mmol, 1.00 equiv.) dissolved in DCM (1.5 mL). Aqueous H_2O_2 solution (35 w/w %, 25 µL, 25 mg, 0.26 mmol, 1.2 equiv.) was added and the mixture was stirred for 1 hour. Water (2 mL) was added and the phases were separated. The aqueous phase was extracted with DCM (3 × 2 mL) and the combined organic phases dried over $MgSO_4$. The mixture was filtered and the solvent evaporated *in vacuo,* affording 101 mg **9a** as a white solid (98 %).

R*f* = 0.27 (cyclohexane:EtOAc, 3:1)

NMR Spectroscopy:

^{1}H NMR (500 MHz, $CDCl_3$, 23 °C): δ = 7.84 – 7.74 (m, 12H) ppm.

^{13}C NMR (125 MHz, $CDCl_3$, 23 °C): δ = 135.5 (dq, J = 103.1, 1.3 Hz), 134.7 (qd, J = 33.0, 2.9 Hz), 132.6 (d, J = 10.2 Hz), 125.9 (dq, J = 12.5, 3.7 Hz), 123.5 (qd, J = 273.1, 1.1 Hz).

^{19}F NMR (471 MHz, $CDCl_3$, 23 °C) δ = −63.4 ppm.

HRMS (ESI): calc'd for $C_{21}H_{13}O_1F_9P_1$ $[M]^+$: 483.0555, found: 483.0558; deviation: +0.7 ppm.

Control experiment with phosphine oxide 9a

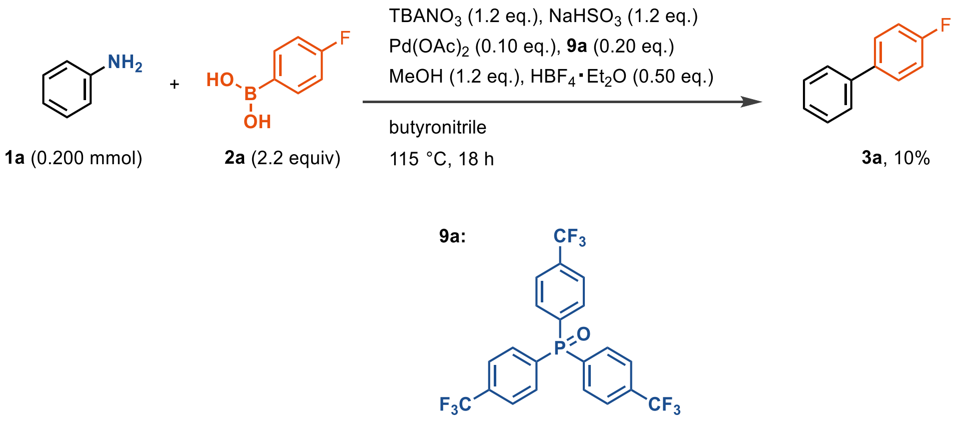

Fig S20. Control experiment with phosphine oxide **9a.**

Procedure

To a 4-mL vial, $TBANO_3$ (73 mg, 0.24 mmol, 1.2 equiv.), $NaHSO_3$ (25 mg, 0.24 mmol, 1.2 equiv.), $Pd(OAc)_2$ (4.5 mg, 0.020 mmol, 0.10 equiv.), **9a** (19.3 mg, 0.0400 mmol, 0.200 equiv.) and **2a** (62 mg, 0.44 mmol, 2.2 equiv.) were added, followed by anhydrous butyronitrile (0.50 mL, c = 0.40 M), aniline (18.0 µL, 18.6 mg, 0.200 mmol, 1.00 equiv.) and MeOH (9.7 µL, 7.7 mg, 0.24 mmol, 1.2 equiv.). The suspension was stirred for 5 minutes at 23 °C and then where applicable $HBF_4{\cdot}OEt_2$ (14 µL, 16 mg, 0.10 mmol, 0.50 equiv.) was added. The vial was sealed with a septum cap and the reaction mixture was heated at 115 °C and stirred (400 rpm) for 18 h. The reaction mixture was cooled to 23 °C and a sat. aq. $NaHCO_3$ (1 mL) solution was added, and the resulting mixture was extracted by EtOAc (3 × 2 mL). The combined organic phases were passed through a pad of silica gel (2 g), and the solvent was evaporated *in vacuo*. The yield was determined by ^{19}F NMR spectroscopy at 298 K and 471 MHz, by dissolving the crude material in $CDCl_3$ (0.5 mL) and adding α,α,α-trifluorotoluene (8.2 µL, 9.8 mg, 67 µmol) as internal standard. The integration of the peak corresponding to the internal standard at −62.9 ppm (3F, s) was compared to the peak corresponding to 3a at −116.2 ppm (1F, (tt, J = 8.7, 5.3 Hz)). The correct peak for **3a** was assigned by comparison to a genuine sample.

Analysis of arylboronic acid homocoupling

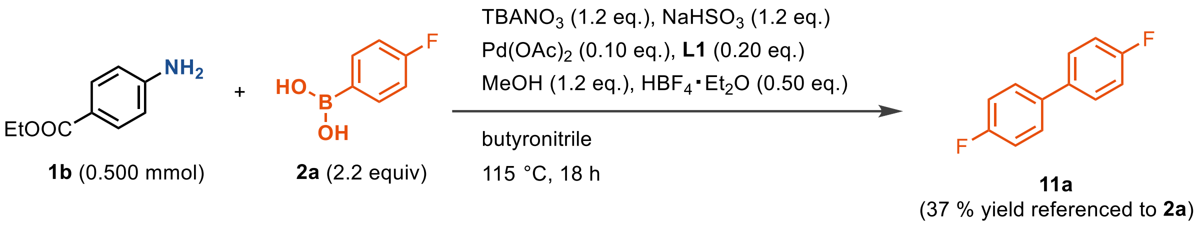

Fig S21. Homocoupling of boronic acid **2a**.

Procedure

To a 4-mL vial, TBANO$_3$ (36.5 mg, 0.12 mmol, 1.20 equiv.), NaHSO$_3$ (12.5 mg, 0.12 mmol, 1.20 equiv.), Pd(OAc)$_2$ (2.2 mg, 0.010 mmol, 0.10 equiv.), **2a** (30.8 mg, 0.22 mmol, 2.2 equiv.) and benzocaine (**1b**) (16.5 mg, 0.100 mmol, 1.00 equiv.) were added followed by anhydrous butyronitrile (0.25 mL, c = 0.40 M) and MeOH (4.8 µL, 3.8 mg, 0.12 mmol, 1.2 equiv.). The suspension was stirred for 5 minutes at 23 °C and then HBF$_4$·OEt$_2$ (6.8 µL, 8.1 mg, 0.050 mmol, 0.50 equiv.) was added. The vial was sealed with a septum cap and the reaction mixture was heated at 115 °C and stirred (400 rpm) for 18 h. The reaction mixture was cooled to 23 °C and NaHCO$_3$ (25 mg, 0.30 mmol, 3 equiv.) was added. The mixture was diluted with cyclohexane (3.5 mL) and passed through a short pad of silica. The resulting solution was evaporated *in vacuo* to afford 15.6 mg (37 % yield compared to **2a**) **11a** as a white solid.

R*f* = 0.51 (cyclohexane)

NMR Spectroscopy:

^{1}H NMR (500 MHz, CDCl$_3$, 23 °C): δ = 7.52 – 7.45 (m, 2H), 7.14 – 7.08 (m, 2H).

^{13}C NMR (125 MHz, CDCl$_3$, 23 °C): δ = 162.6 (d, J = 246.6 Hz), 136.6 (d, J = 3.0 Hz), 128.7 (d, J = 8.0 Hz), 115.8 (d, J = 21.4 Hz).

^{19}F NMR (471 MHz, CDCl$_3$, 23 °C) δ = −115.8 (tt, J = 8.7, 5.3 Hz)) ppm.

HRMS (EI): calc'd for $C_{12}H_8F_2$ $[M]^+$: 190.0589, found: 190.0590; deviation: +0.8 ppm.

Substrate Scope of Suzuki-Miyaura cross-coupling of anilines.

Benzocaine-derived biphenyl 3b

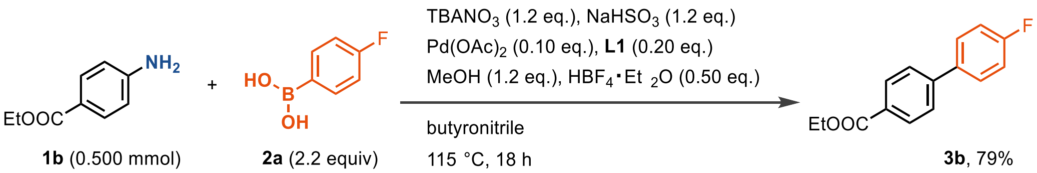

To a 4-mL vial, TBANO$_3$ (182 mg, 0.600 mmol, 1.20 equiv.), NaHSO$_3$ (62 mg, 0.600 mmol, 1.2 equiv.), Pd(OAc)$_2$ (12 mg, 0.050 mmol, 0.10 equiv.), **L1** (47 mg, 0.10 mmol, 0.20 equiv.), **2a** (154 mg, 1.10 mmol, 2.20 equiv.) and benzocaine (**1b**) (82.6 mg, 0.500 mmol, 1.00 equiv.) were added followed by anhydrous *n*-

PrCN (1.25 mL, c = 0.400 M) and MeOH (24.0 µL, 19.0 mg, 0.600 mmol, 1.20 equiv.). The suspension was stirred for 5 minutes at 23 °C and then $HBF_4 \cdot OEt_2$ (34 µL, 40 mg, 0.25 mmol, 0.50 equiv.) was added. The vial was sealed with a septum cap and the reaction mixture was heated at 115 °C and stirred (400 rpm) for 18 h. The reaction mixture was cooled to 23 °C and a sat. aq. $NaHCO_3$ (1 mL) solution was added, and the resulting mixture was extracted by EtOAc (3 × 2 mL). The combined organic phases were passed through a pad of silica gel (2 g), and the solvent was evaporated *in vacuo*. The crude material was purified by flash column chromatography (SiO_2, hexane:EtOAc = 19:1) affording 96 mg (79 % yield) of **3b** as an off-white solid.

$\mathbf{R}_f$ = 0.44 (hexane:EtOAc = 19:1)

NMR Spectroscopy:

^{1}H NMR (500 MHz, $CDCl_3$, 23 °C): δ = 8.15 – 8.09 (m, 2H), 7.65 – 7.55 (m, 4H), 7.21 – 7.13 (m, 2H), 4.43 (q, J = 7.1 Hz, 2H), 1.44 (t, J = 7.1 Hz, 3H) ppm.

^{13}C NMR (125 MHz, $CDCl_3$, 23 °C): δ = 166.5, 163.0 (d, J = 247.6 Hz), 144.5, 136.2 (d, J = 3.4 Hz), 130.2, 129.4, 129.0, 129.0 (d, J = 8.2 Hz), 115.9 (d, J = 21.8 Hz), 61.1, 14.44 ppm.

^{19}F NMR (471 MHz, $CDCl_3$, 23 °C) δ = −114.3 (tt, J = 8.5, 5.3 Hz) ppm.

HRMS (EI): calc'd for $C_{15}H_{13}O_2F_1$ $[M]^+$: 244.0894, found: 244.0896; deviation: +0.8 ppm.

Benzocaine-derived biphenyl 3c

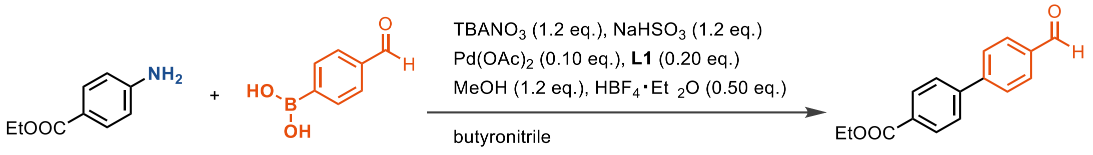

To a 4-mL vial, $TBANO_3$ (182 mg, 0.600 mmol, 1.20 equiv.), $NaHSO_3$ (62 mg, 0.600 mmol, 1.2 equiv.), $Pd(OAc)_2$ (12 mg, 0.050 mmol, 0.10 equiv.), **L1** (47 mg, 0.10 mmol, 0.20 equiv.), **2a** (154 mg, 1.10 mmol, 2.20 equiv.) and benzocaine (**1b**) (82.6 mg, 0.500 mmol, 1.00 equiv.) were added followed by anhydrous *n*-PrCN (1.25 mL, c = 0.400 M) and MeOH (24.0 µL, 19.0 mg, 0.600 mmol, 1.20 equiv.). The suspension was stirred for 5 minutes at 23 °C and then $HBF_4 \cdot OEt_2$ (34 µL, 40 mg, 0.25 mmol, 0.50 equiv.) was added. The vial was sealed with a septum cap and the reaction mixture was heated at 115 °C and stirred (400 rpm) for 18 h. The reaction mixture was cooled to 23 °C and a sat. aq. $NaHCO_3$ (1 mL) solution was added, and the resulting mixture was extracted by EtOAc (3 × 2 mL). The combined organic phases were passed through a pad of silica gel (2 g), and the solvent was evaporated *in vacuo*. The crude material was purified by flash column chromatography (SiO_2, hexane:EtOAc = 88:12) affording 64 mg (50 % yield) **3c** as a colorless solid.

$\mathbf{R}_f$ = 0.26 (hexane:EtOAc = 9:1)

NMR Spectroscopy:

^{1}H NMR (500 MHz, $CDCl_3$, 23 °C): δ = 10.08 (s, 1H), 8.16 – 8.13 (m, 2H), 8.00 – 7.97 (m, 2H), 7.80 –

7.77 (m, 2H), 7.71 – 7.69 (m, 2H), 4.42 (q, J = 7.1 Hz, 2H), 1.42 (t, J = 7.1 Hz, 3H) ppm.

^{13}C NMR (125 MHz, $CDCl_3$, 23 °C): δ = 191.85, 166.30, 145.98, 143.98, 135.84, 130.43, 130.39, 130.31, 127.98, 127.38, 61.24, 14.43 ppm.

HRMS (EI): calc'd for $C_{16}H_{14}O_3$ $[M]^+$: 254.0937, found: 254.0938; deviation: +0.4 ppm.

4-Aminobenzophenone-derived biphenyl 3d

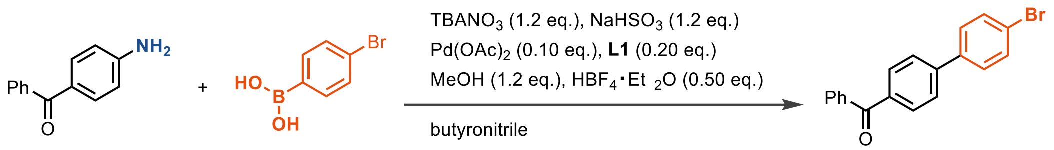

1c (0.500 mmol) **2c** (2.2 equiv) **3d**, 51%

To a 4-mL vial, $TBANO_3$ (182 mg, 0.600 mmol, 1.20 equiv.), $NaHSO_3$ (62 mg, 0.600 mmol, 1.2 equiv.), $Pd(OAc)_2$ (12 mg, 0.050 mmol, 0.10 equiv.), **L1** (47 mg, 0.10 mmol, 0.20 equiv.), **2c** (221 mg, 1.10 mmol, 2.20 equiv.) and **1c** (99.0 mg, 0.500 mmol, 1.0 equiv.) were added followed by anhydrous *n*-PrCN (1.25 mL, c = 0.400 M) and MeOH (24.0 µL, 19.0 mg, 0.600 mmol, 1.20 equiv.). The suspension was stirred for 5 minutes at 23 °C and then $HBF_4{\cdot}OEt_2$ (34 µL, 40 mg, 0.25 mmol, 0.50 equiv.) was added. The vial was sealed with a septum cap and the reaction mixture was heated at 115 °C and stirred (400 rpm) for 18 h. The reaction mixture was cooled to 23 °C and a sat. aq. $NaHCO_3$ (1 mL) solution was added, and the resulting mixture was extracted by EtOAc (3 × 2 mL). The combined organic phases were passed through a pad of silica gel (2 g), and the solvent was evaporated *in vacuo*. The crude material was purified by flash column chromatography (SiO_2, Hexane:EtOAc = 19:1) affording 86 mg (51 % yield) of **3d** as a colorless solid.

R*f* = 0.42 (Hexane:EtOAc = 19:1)

NMR Spectroscopy:

^{1}H NMR (500 MHz, $CDCl_3$, 23 °C): δ = 7.92 – 7.86 (m, 2H), 7.86 – 7.81 (m, 2H), 7.70 – 7.64 (m, 2H), 7.64 – 7.59 (m, 3H), 7.55 – 7.48 (m, 4H) ppm.

^{13}C NMR (125 MHz, $CDCl_3$, 23 °C): δ = 196.3, 144.1, 139.0, 137.8, 136.7, 132.6, 132.3, 130.9, 130.1, 129.0, 128.5, 126.9, 122.8 ppm.

HRMS (EI): calc'd for $C_{19}H_{13}O_1Br_1$ $[M]^+$: 336.0144, found: 336.0147; deviation: +0.9 ppm.

4-Chloro-3-(trifluoromethyl)aniline-derived biphenyl 3e

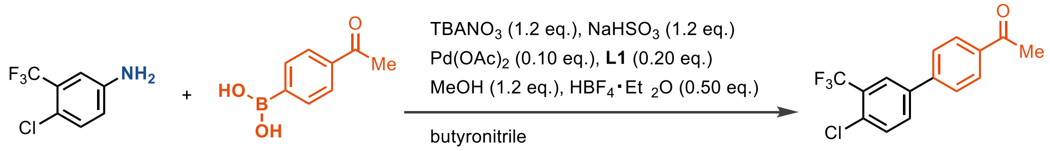

1d (0.500 mmol) **2d** (2.2 equiv) **3e**, 46%

To a 4-mL vial, $TBANO_3$ (182 mg, 0.600 mmol, 1.20 equiv.), $NaHSO_3$ (62 mg, 0.600 mmol, 1.2 equiv.), $Pd(OAc)_2$ (12 mg, 0.050 mmol, 0.10 equiv.), **L1** (47 mg, 0.10 mmol, 0.20 equiv.), **2d** (180 mg, 1.10 mmol,

2.20 equiv.) and **1d** (98.0 mg, 0.500 mmol, 1.00 equiv.) were added followed by anhydrous *n*-PrCN (1.25 mL, c = 0.400 M) and MeOH (24.0 µL, 19.0 mg, 0.600 mmol, 1.20 equiv.). The suspension was stirred for 5 minutes at 23 °C and then $HBF_4·OEt_2$ (34 µL, 40 mg, 0.25 mmol, 0.50 equiv.) was added. The vial was sealed with a septum cap and the reaction mixture was heated at 115 °C and stirred (400 rpm) for 18 h. The reaction mixture was cooled to 23 °C and a sat. aq. $NaHCO_3$ (1 mL) solution was added, and the resulting mixture was extracted by EtOAc (3 × 2 mL). The combined organic phases were passed through a pad of silica gel (2 g), and the solvent was evaporated *in vacuo*. The crude material was purified by flash column chromatography (SiO_2, pentane:EtOAc = 20:1) affording 69 mg of **3e** as an off-white solid.

R*f* = 0.40 (pentane:EtOAc = 20:1)

NMR Spectroscopy:

^{1}H NMR (500 MHz, $CDCl_3$, 23 °C): *δ* = 8.05 (m, 2H), 7.91 (dm, *J* = 2.3, 1H), 7.72 (ddq, *J* = 8.3, 2.3, 0.5 Hz, 1H), 7.67 – 7.62 (m, 2H), 7.61 (dm, *J* = 8.3 Hz, 1H), 2.64 (s, 3H) ppm.

^{13}C NMR (125 MHz, $CDCl_3$, 23 °C): *δ* = 197.6, 143.2, 139.0, 136.8, 132.4 (q, *J* = 1.9 Hz), 131.5, 129.3, 129.2 (q, *J* = 31.6 Hz), 127.4, 126.5 (q, *J* = 5.3 Hz), 122.9 (q, *J* = 273.4 Hz), 26.9 ppm.

^{19}F NMR (471 MHz, $CDCl_3$, 23 °C): *δ* = −62.7 (s) ppm.

HRMS (EI): calc'd for $C_{15}H_{10}ClF_3O$ $[M]^+$: 298.0367, found: 298.0367; deviation: 0.0 ppm.

3-(Benzyloxy)aniline-derived biphenyl 3f

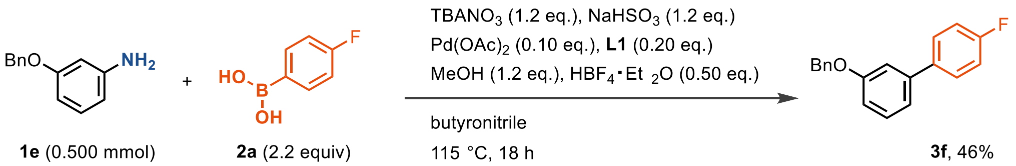

To a 4-mL vial, $TBANO_3$ (182 mg, 0.600 mmol, 1.20 equiv.), $NaHSO_3$ (62 mg, 0.600 mmol, 1.2 equiv.), $Pd(OAc)_2$ (12 mg, 0.050 mmol, 0.10 equiv.), **L1** (47 mg, 0.10 mmol, 0.20 equiv.), **2a** (154 mg, 1.10 mmol, 2.20 equiv.) and **1e** (100 mg, 0.500 mmol, 1.00 equiv.) were added followed by anhydrous *n*-PrCN (1.25 mL, c = 0.400 M) and MeOH (24.0 µL, 19.0 mg, 0.600 mmol, 1.20 equiv.). The suspension was stirred for 5 minutes at 23 °C and then $HBF_4·OEt_2$ (34 µL, 40 mg, 0.25 mmol, 0.50 equiv.) was added. The vial was sealed with a septum cap and the reaction mixture was heated at 115 °C and stirred (400 rpm) for 18 h. The reaction mixture was cooled to 23 °C and a sat. aq. $NaHCO_3$ (1 mL) solution was added, and the resulting mixture was extracted by EtOAc (3 × 2 mL). The combined organic phases were passed through a pad of silica gel (2 g), and the solvent was evaporated *in vacuo*. The crude material was purified by flash column chromatography (SiO_2, hexane:EtOAc = 19:1) affording 64 mg (46 % yield) of **3f** as colorless crystals.

R*f* = 0.59 (hexane:EtOAc = 19:1)

NMR Spectroscopy:

^{1}H NMR (500 MHz, $CDCl_3$, 23 °C): *δ* = 7.58 – 7.52 (m, 2H), 7.50 – 7.47 (m, 2H), 7.46 – 7.40 (m, 2H),

7.40 – 7.34 (m, 2H), 7.21 – 7.10 (m, 4H), 6.99 (ddd, *J* = 8.3, 2.7, 0.8 Hz, 1H), 5.15 (s, 2H) ppm.

^{13}C NMR (125 MHz, $CDCl_3$, 23 °C): *δ* = 162.7 (d, *J* = 246.7 Hz), 159.3, 141.9, 137.2 (d, *J* = 3.1 Hz), 130.0, 128.8 (d, *J* = 7.9 Hz), 128.8, 128.2, 127.7, 119.9, 115.7 (d, *J* = 21.4 Hz), 114.0, 113.6, 70.2 ppm.

^{19}F NMR (471 MHz, $CDCl_3$, 23 °C): *δ* = −115.6 (tt, *J* = 8.6, 5.3 Hz) ppm.

HRMS (EI): calc'd for $C_{19}H_{15}O_1F_1$ $[M]^+$: 278.1101, found: 278.1106; deviation: +1.8 ppm.

4-Aminoacetophenone-derived biphenyl 3g

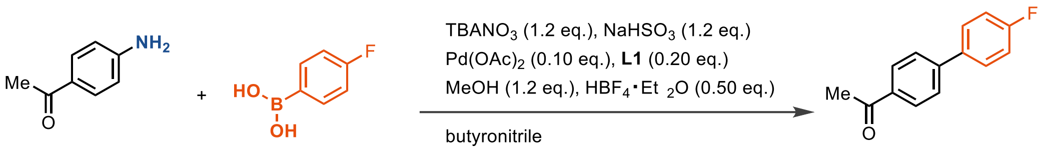

1f (0.500 mmol) **2a** (2.2 equiv) **3g**, 72%

To a 4-mL vial, $TBANO_3$ (182 mg, 0.600 mmol, 1.20 equiv.), $NaHSO_3$ (62 mg, 0.600 mmol, 1.2 equiv.), $Pd(OAc)_2$ (12 mg, 0.050 mmol, 0.10 equiv.), **L1** (47 mg, 0.10 mmol, 0.20 equiv.), **2a** (154 mg, 1.10 mmol, 2.20 equiv.) and **1f** (68.0 mg, 0.500 mmol, 1.00 equiv.) were added followed by anhydrous *n*-PrCN (1.25 mL, c = 0.400 M) and MeOH (24.0 µL, 19.0 mg, 0.600 mmol, 1.20 equiv.). The suspension was stirred for 5 minutes at 23 °C and then $HBF_4{\cdot}OEt_2$ (34 µL, 40 mg, 0.25 mmol, 0.50 equiv.) was added. The vial was sealed with a septum cap and the reaction mixture was heated at 115 °C and stirred (400 rpm) for 18 h. The reaction mixture was cooled to 23 °C and a sat. aq. $NaHCO_3$ (1 mL) solution was added, and the resulting mixture was extracted by EtOAc (3 × 2 mL). The combined organic phases were passed through a pad of silica gel (2 g), and the solvent was evaporated *in vacuo*. The crude material was purified by flash column chromatography (SiO_2, Hexane:EtOAc = 19:1) affording 78 mg (72 % yield) of **3g** as a colorless solid.

R*f* = 0.25 (Hexane:EtOAc = 19:1)

NMR Spectroscopy:

^{1}H NMR (500 MHz, $CDCl_3$, 23 °C): *δ* = 8.06 – 7.97 (m, 2H), 7.69 – 7.61 (m, 2H), 7.60 – 7.56 (m, 2H), 7.20 – 7.12 (m, 2H), 2.63 (s, 3H) ppm.

^{13}C NMR (125 MHz, $CDCl_3$, 23 °C): *δ* = 197.8, 163.1 (d, *J* = 248.0 Hz), 144.8, 136.1 (d, *J* = 3.5 Hz), 136.0, 129.1, 129.0 (d, *J* = 7.9 Hz), 127.2, 116.0 (d, *J* = 21.4 Hz), 26.8 ppm.

^{19}F NMR (471 MHz, $CDCl_3$, 23 °C): *δ* = −114.0 (tt, *J* = 8.6, 5.3) ppm.

HRMS (EI): calc'd for $C_{14}H_{11}O_1F_1$ $[M]^+$: 214.0790, found: 214.0788; deviation: −0.9 ppm.

4-Fluoro-1,1':2',1''-terphenyl 3h

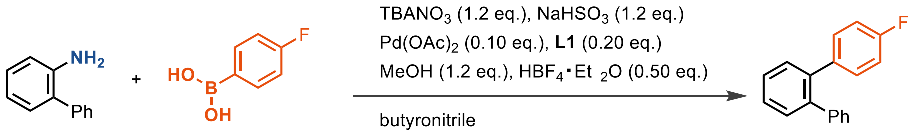

1g (0.500 mmol) **2a** (2.2 equiv) **3h**, 97%

To a 4-mL vial, $TBANO_3$ (182 mg, 0.600 mmol, 1.20 equiv.), $NaHSO_3$ (62 mg, 0.600 mmol, 1.2 equiv.), $Pd(OAc)_2$ (12 mg, 0.050 mmol, 0.10 equiv.), **L1** (47 mg, 0.10 mmol, 0.20 equiv.), **2a** (154 mg, 1.10 mmol, 2.20 equiv.) and **1g** (85.0 mg, 0.500 mmol, 1.00 equiv.) were added followed by anhydrous *n*-PrCN (1.25 mL, c = 0.400 M) and MeOH (24.0 µL, 19.0 mg, 0.600 mmol, 1.20 equiv.). The suspension was stirred for 5 minutes at 23 °C and then $HBF_4{\cdot}OEt_2$ (34 µL, 40 mg, 0.25 mmol, 0.50 equiv.) was added. The vial was sealed with a septum cap and the reaction mixture was heated at 115 °C and stirred (400 rpm) for 18 h. The reaction mixture was cooled to 23 °C and a sat. aq. $NaHCO_3$ (1 mL) solution was added, and the resulting mixture was extracted by EtOAc (3 × 2 mL). The combined organic phases were passed through a pad of silica gel (2 g), and the solvent was evaporated *in vacuo*. The crude material was purified by flash column chromatography (SiO_2, hexane). affording 121 mg (97% yield) of **3h** as an off-white solid.

R_f = 0.5 (hexane).

NMR Spectroscopy:

^{1}H NMR (500 MHz, $CDCl_3$, 23 °C): δ = 7.52 – 7.45 (m, 4H), 7.33 – 7.25 (m, 3H), 7.24 – 7.13 (m, 4H), 7.03 – 6.91 (m, 2H) ppm.

^{13}C NMR (125 MHz, $CDCl_3$, 23 °C): δ = 161.9 (d, *J* = 245.6 Hz), 141.5, 140.8, 139.7, 137.6 (d, *J* = 3.4 Hz), 131.5 (d, *J* = 8.0 Hz), 130.8, 130.6, 130.0, 128.1, 127.7, 127.7, 126.7, 115.0, 114.9 ppm.

HRMS (EI): calc'd for $C_{18}H_{13}F$ $[M]^+$: 248.0996, found: 248.0999; deviation: +1.2 ppm.

2-Bromo-3-chloroaniline-derived biphenyl 3i

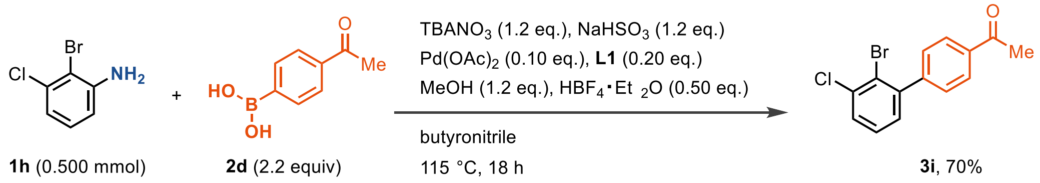

To a 4-mL vial, $TBANO_3$ (182 mg, 0.600 mmol, 1.20 equiv.), $NaHSO_3$ (62 mg, 0.600 mmol, 1.2 equiv.), $Pd(OAc)_2$ (12 mg, 0.050 mmol, 0.10 equiv.), **L1** (47 mg, 0.10 mmol, 0.20 equiv.), **2d** (180 mg, 1.10 mmol, 2.20 equiv.) and 2-bromo-3-chloroaniline (**1h**) (103 mg, 0.500 mmol, 1.00 equiv.) were added followed by anhydrous *n*-PrCN (1.25 mL, c = 0.400 M) and MeOH (24.0 µL, 19.0 mg, 0.600 mmol, 1.20 equiv.). The suspension was stirred for 5 minutes at 23 °C and then $HBF_4{\cdot}OEt_2$ (34 µL, 40 mg, 0.25 mmol, 0.50 equiv.) was added. The vial was sealed with a septum cap and the reaction mixture was heated at 115 °C and stirred (400 rpm) for 18 h. The reaction mixture was cooled to 23 °C and a sat. aq. $NaHCO_3$ (1 mL) solution was added, and the resulting mixture was extracted by EtOAc (3 × 2 mL). The combined organic phases were passed through a pad of silica gel (2 g), and the solvent was evaporated *in vacuo*. The crude material was purified by flash column chromatography (SiO_2, pentane:EtOAc = 20:1) affording 109 mg (70% yield) of **3i** as an off-white solid.

R_f = 0.4 (hexane:EtOAc = 10:1).

NMR Spectroscopy:

^{1}H NMR (500 MHz, $CDCl_3$, 23 °C): δ = 8.10 – 7.97 (m, 2H), 7.50 (dd, J = 8.0, 1.6 Hz, 1H), 7.48 – 7.45 (m, 2H), 7.31 (t, J = 7.8 Hz, 1H), 7.18 (dd, J = 7.6, 1.6 Hz, 1H), 2.65 (s, 3H) ppm.

^{13}C NMR (125 MHz, $CDCl_3$, 23 °C): δ = 197.8, 146.0, 144.3, 136.5, 135.9, 130.0, 129.7, 129.0, 128.3, 128.1, 122.9, 26.8 ppm.

HRMS (EI): calc'd for $C_{14}H_{10}BrClO$ $[M]^+$: 307.9598, found: 307.9599; deviation: +0.3 ppm.

Sulfadoxin-derived biphenyl 3j

0.5 mmol scale reaction

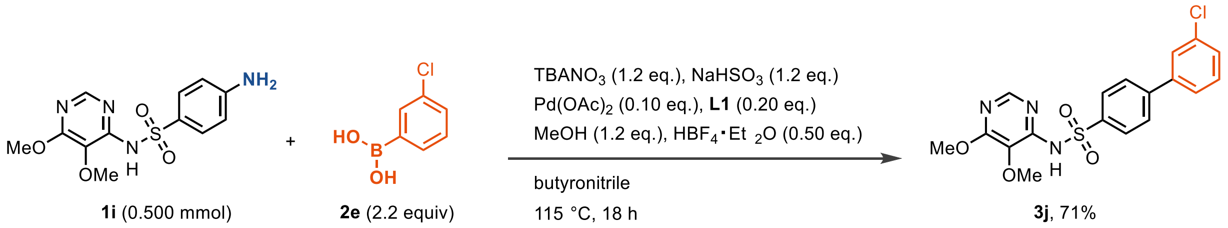

To a 4-mL vial, $TBANO_3$ (182 mg, 0.600 mmol, 1.20 equiv.), $NaHSO_3$ (62 mg, 0.600 mmol, 1.2 equiv.), $Pd(OAc)_2$ (12 mg, 0.050 mmol, 0.10 equiv.), **L1** (47 mg, 0.10 mmol, 0.20 equiv.), (3-chlorophenyl)boronic acid (**2e**) (172 mg, 1.10 mmol, 2.20 equiv.) and sulfadoxin (**1i**) (155 mg, 0.500 mmol, 1.00 equiv were added followed by anhydrous *n*-PrCN (1.25 mL, c = 0.400 M) and MeOH (24.0 µL, 19.0 mg, 0.600 mmol, 1.20 equiv.). The suspension was stirred for 5 minutes at 23 °C and then $HBF_4{\cdot}OEt_2$ (34 µL, 40 mg, 0.25 mmol, 0.50 equiv.) was added. The vial was sealed with a septum cap and the reaction mixture was heated at 115 °C and stirred (400 rpm) for 18 h. The reaction mixture was cooled to 23 °C and a sat. aq. $NaHCO_3$ (1 mL) solution was added, and the resulting mixture was extracted by EtOAc (3 × 2 mL). The combined organic phases were passed through a pad of silica gel (2 g), and the solvent was evaporated *in vacuo*. The crude material was purified by flash column chromatography (SiO_2, hexane:EtOAc = 2:1) affording 144 mg (71 % yield) of **3j** as a colorless solid.

5 mmol scale reaction

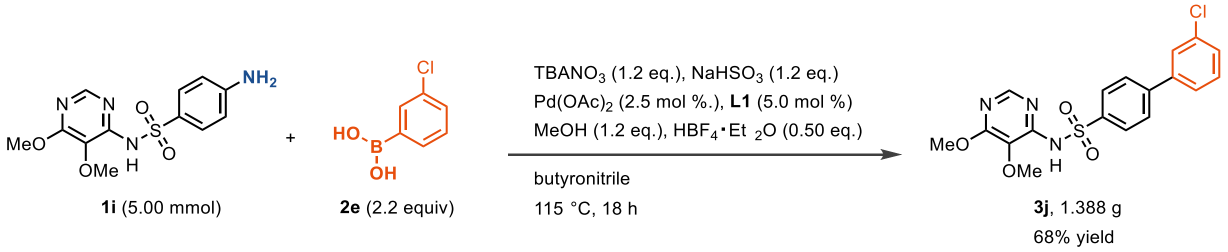

To a 40-mL vial, $TBANO_3$ (1.82 g, 6.00 mmol, 1.20 equiv.), $NaHSO_3$ (624 mg, 6.00 mmol, 1.20 equiv.), $Pd(OAc)_2$ (28.1 mg, 0.125 mmol, 2.50 mol%), **L1** (114 mg, 0.250 mmol, 5.00 mol%), (3-chlorophenyl)boronic

acid (**2e**) (1.72 g, 11.0 mmol, 2.20 equiv.) and sulfadoxin (**1i**) (1.55 g, 5.00 mmol, 1.00 equiv.) were added followed by anhydrous *n*-PrCN (12.5 mL, c = 0.400 M) and MeOH (243 µL, 192 mg, 6.00 mmol, 1.20 equiv.). The suspension was stirred for 5 minutes at 23 °C and then $HBF_4{\cdot}OEt_2$ (340 µL, 404 mg, 3.00 mmol, 0.500 equiv.) was added. The vial was sealed with a septum cap and the reaction mixture was heated at 115 °C and stirred (400 rpm) for 18 h. The reaction mixture was cooled to 23 °C and a sat. aq. $NaHCO_3$ (10 mL) solution was added, and the resulting mixture was extracted by EtOAc (3 x 10 mL). The combined organic phases were dried using $MgSO_4$ and filtered through Celite. The filtrates were evaporated *in vacuo* and the crude material was purified by flash column chromatography (SiO_2, hexane:EtOAc = 2:1) affording 1.388 g (68 % yield) of **3j** as a colorless solid.

$\mathbf{R}_f$ = 0.27 (hexane:EtOAc = 3:1)

NMR Spectroscopy:

^{1}H NMR (500 MHz, $CDCl_3$, 23 °C): δ = 8.24 – 8.21 (m, 2H), 8.18 (s, 1H), 7.71 – 7.64 (m, 2H), 7.57 (ddd, J = 1.8, 1.7, 0.8 Hz, 1H), 7.46 (ddd, J = 6.8, 2.0, 2.0 Hz, 1H), 7.43 – 7.36 (m, 2H), 3.98 (s, 3H), 3.88 (s, 3H) ppm.

^{13}C NMR (125 MHz, $CDCl_3$, 23 °C): δ = 161.0, 151.2, 149.8, 144.9, 141.2, 138.8, 135.1, 130.4, 129.2, 128.7, 127.6, 127.5, 126.7, 125.6, 60.7, 54.3 ppm.

HRMS (ESI): calc'd for $C_{18}H_{17}O_4N_3S_1Cl_1$ $[M+H]^+$: 406.0622, found: 406.0620; deviation: −0.5 ppm.

4-Methylcoumarin-derived biphenyl 3k

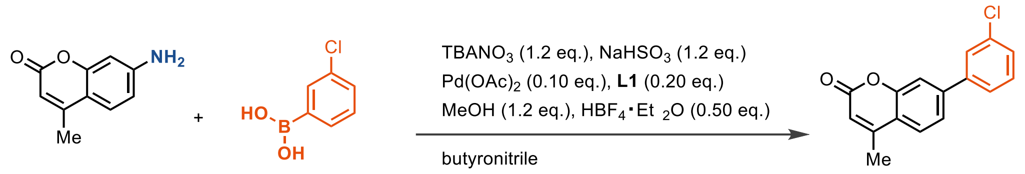

1j (0.500 mmol) **2e** (2.2 equiv) **3k**, 71%

To a 4-mL vial, $TBANO_3$ (182 mg, 0.600 mmol, 1.20 equiv.), $NaHSO_3$ (62 mg, 0.600 mmol, 1.2 equiv.), $Pd(OAc)_2$ (12 mg, 0.050 mmol, 0.10 equiv.), **L1** (47 mg, 0.10 mmol, 0.20 equiv.), (3-chlorophenyl)boronic acid (**2e**) (172 mg, 1.10 mmol, 2.2 equiv.) and **1j** (87.6 mg, 0.500 mmol, 1.00 equiv were added followed by anhydrous *n*-PrCN (1.25 mL, c = 0.400 M) and MeOH (24.0 µL, 19.0 mg, 0.600 mmol, 1.20 equiv.). The suspension was stirred for 5 minutes at 23 °C and then $HBF_4{\cdot}OEt_2$ (34 µL, 40 mg, 0.25 mmol, 0.50 equiv.) was added. The vial was sealed with a septum cap and the reaction mixture was heated at 115 °C and stirred (400 rpm) for 18 h. The reaction mixture was cooled to 23 °C and a sat. aq. $NaHCO_3$ (1 mL) solution was added, and the resulting mixture was extracted by EtOAc (3 × 2 mL). The combined organic phases were passed through a pad of silica gel (2 g), and the solvent was evaporated *in vacuo*. The crude material was purified by flash column chromatography (SiO_2, hexane:EtOAc = 3:1) affording 83 mg (61 % yield) of **3k** as an off-white solid.

$\mathbf{R}_f$ = 0.44 (hexane:EtOAc = 3:1)

NMR Spectroscopy:

^{1}H NMR (500 MHz, $CDCl_3$, 23 °C): δ = 7.68 – 7.64 (m, 1H), 7.61 – 7.57 (m, 1H), 7.51 – 7.47 (m, 3H), 7.44 – 7.36 (m, 2H), 6.32 – 6.29 (m, 1H), 2.47 – 2.46 (m, 3H) ppm.

^{13}C NMR (125 MHz, $CDCl_3$, 23 °C): δ = 160.8, 154.0, 152.1, 143.4, 141.1, 135.2, 130.5, 128.6, 127.4, 125.5, 125.3, 123.1, 119.5, 115.4, 115.3, 18.8 ppm.

HRMS (EI): calc'd for $C_{16}H_{11}O_2Cl_1$ $[M]^+$: 270.0444, found: 270.0442; deviation: −0.7 ppm.

4-Amino-3,5-dimethylbenzonitrile-derived biphenyl 3l

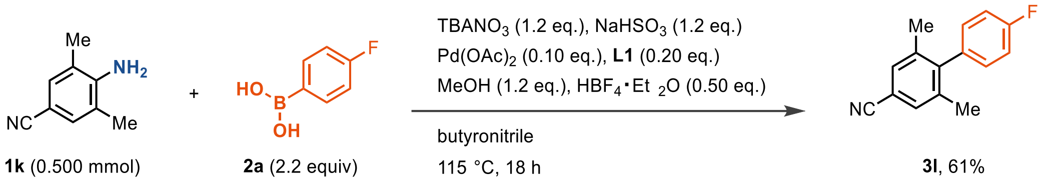

To a 4-mL vial, $TBANO_3$ (182 mg, 0.600 mmol, 1.20 equiv.), $NaHSO_3$ (62 mg, 0.600 mmol, 1.2 equiv.), $Pd(OAc)_2$ (12 mg, 0.050 mmol, 0.10 equiv.), **L1** (47 mg, 0.10 mmol, 0.20 equiv.), **2a** (154 mg, 1.10 mmol, 2.20 equiv.) and **1k** (73 mg, 0.500 mmol, 1.00 equiv.) were added followed by anhydrous *n*-PrCN (1.25 mL, c = 0.400 M) and MeOH (24.0 µL, 19.0 mg, 0.600 mmol, 1.20 equiv.). The suspension was stirred for 5 minutes at 23 °C and then $HBF_4{\cdot}OEt_2$ (34 µL, 40 mg, 0.25 mmol, 0.50 equiv.) was added. The vial was sealed with a septum cap and the reaction mixture was heated at 115 °C and stirred (400 rpm) for 18 h. The reaction mixture was cooled to 23 °C and a sat. aq. $NaHCO_3$ (1 mL) solution was added, and the resulting mixture was extracted by EtOAc (3 × 2 mL). The combined organic phases were passed through a pad of silica gel (2 g), and the solvent was evaporated *in vacuo*. The crude material was purified by flash column chromatography (SiO_2, hexane:EtOAc = 50:1) affording 69 mg (61% yield) of **3l** as a white-off solid.

R*f* = 0.5 (Hexane:EtOAc = 18:1)

NMR Spectroscopy:

^{1}H NMR (500 MHz, $CDCl_3$, 23 °C): δ = 7.39 (t, *J* = 0.7 Hz, 2H), 7.16 (m, 2H), 7.06 (m, 2H), 2.04 (t, *J* = 0.7 Hz, 6H) ppm.

^{13}C NMR (125 MHz, $CDCl_3$, 23 °C): δ = 162.1 (d, *J* = 246.4 Hz), 145.7, 137.8, 135.0 (d, *J* = 3.5 Hz), 130.9, 129.9 (d, *J* = 8.0 Hz), 119.0, 115.8 (d, *J* = 21.4 Hz), 111.0, 20.7 ppm.

HRMS (EI): calc'd for $C_{15}H_{12}FN$ $[M]^+$: 225.0948, found: 225.0951; deviation: +1.3 ppm.

2-Aminophenol-derived biphenyl 3m

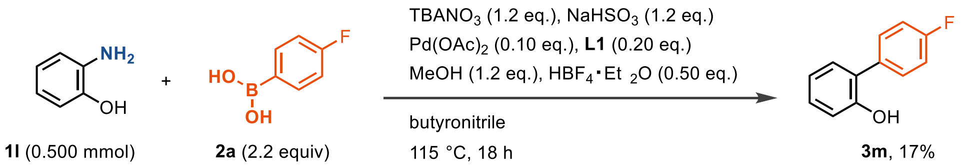

To a 4-mL vial, $TBANO_3$ (182 mg, 0.600 mmol, 1.20 equiv.), $NaHSO_3$ (62 mg, 0.600 mmol, 1.2 equiv.), $Pd(OAc)_2$ (12 mg, 0.050 mmol, 0.10 equiv.), **L1** (47 mg, 0.10 mmol, 0.20 equiv.), **2a** (154 mg, 1.10 mmol, 2.20 equiv.) and 2-aminophenol (**1l**) (55.0 mg, 0.500 mmol, 1.00 equiv.) were added followed by anhydrous *n*-PrCN (1.25 mL, c = 0.400 M) and MeOH (24.0 µL, 19.0 mg, 0.600 mmol, 1.20 equiv.). The suspension was stirred for 5 minutes at 23 °C and then $HBF_4{\cdot}OEt_2$ (34 µL, 40 mg, 0.25 mmol, 0.50 equiv.) was added. The vial was sealed with a septum cap and the reaction mixture was heated at 115 °C and stirred (400 rpm) for 18 h. The reaction mixture was cooled to 23 °C and a sat. aq. $NaHCO_3$ (1 mL) solution was added, and the resulting mixture was extracted by EtOAc (3 × 2 mL). The combined organic phases were passed through a pad of silica gel (2 g), and the solvent was evaporated *in vacuo*. The crude material was purified by flash column chromatography (SiO_2, hexane:EtOAc = 93:7) affording 16 mg (17 % yield) of **3m** as a yellow oil.

R*f* = 0.28 (hexane:EtOAc = 9:1).

NMR Spectroscopy:

^{1}H NMR (500 MHz, $CDCl_3$, 23 °C): *δ* = 7.46 (d, *J* = 1.2 Hz, 2H), 7.27 (ddd, *J* = 8.1, 7.4, 1.7 Hz, 1H), 7.22 (ddd, *J* = 7.6, 1.7, 0.4 Hz, 1H), 7.21 – 7.15 (m, 2H), 7.00 (td, *J* = 7.5, 1.3 Hz, 1H), 6.97 (ddd, *J* = 8.1, 1.2, 0.4 Hz, 1H), 5.07 (s, 1H) ppm.

^{13}C NMR (125 MHz, $CDCl_3$, 23 °C): *δ* = 162.6 (d, *J* = 247.8 Hz), 152.5, 133.2 (d, *J* = 3.0 Hz), 131.0 (d, *J* = 7.8 Hz), 130.5, 129.4, 127.4, 121.1, 116.2 (d, *J* = 21.4 Hz), 116.1 ppm.

^{19}F NMR (471 MHz, $CDCl_3$, 23 °C): *δ* = −114.3 (tt, *J* = 8.7, 5.4 Hz) ppm.

HRMS (EI): calc'd for $C_{12}H_9O_1F_1$ $[M]^+$: 188.0631, found: 188.0633; deviation: +1.1 ppm.

3-(4-Fluorophenyl)quinoline 3n

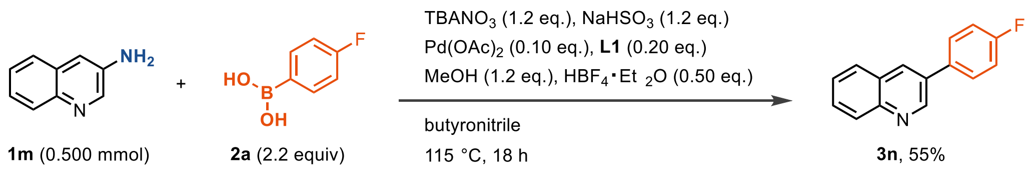

To a 4-mL vial, $TBANO_3$ (182 mg, 0.600 mmol, 1.20 equiv.), $NaHSO_3$ (62 mg, 0.600 mmol, 1.2 equiv.), $Pd(OAc)_2$ (12 mg, 0.050 mmol, 0.10 equiv.), **L1** (47 mg, 0.10 mmol, 0.20 equiv.), **2a** (154 mg, 1.10 mmol, 2.20 equiv.) and quinolin-3-amine (**1m**) (72.0 mg, 0.500 mmol, 1.00 equiv.) were added followed by anhydrous *n*-PrCN (1.25 mL, c = 0.400 M) and MeOH (24.0 µL, 19.0 mg, 0.600 mmol, 1.20 equiv.). The suspension was stirred for 5 minutes at 23 °C and then $HBF_4{\cdot}OEt_2$ (34 µL, 40 mg, 0.25 mmol, 0.50 equiv.) was added. The vial was sealed with a septum cap and the reaction mixture was heated at 115 °C and stirred (400 rpm) for 18 h. The reaction mixture was cooled to 23 °C and a sat. aq. $NaHCO_3$ (1 mL) solution was added, and the resulting mixture was extracted by EtOAc (3 × 2 mL). The combined organic phases were passed through a pad of silica gel (2 g), and the solvent was evaporated *in vacuo*. The crude material was purified by flash column chromatography (SiO_2, hexane:EtOAc = 82:18) affording 62 mg (55 % yield) of **3n** as an off-white solid.

R*f* = 0.40 (hexane:EtOAc = 3:1).

NMR Spectroscopy:

^{1}H NMR (500 MHz, $CDCl_3$, 23 °C): *δ* = 9.12 (d, *J* = 2.4 Hz, 1H), 8.21 (dd, *J* = 2.4, 1.1 Hz, 1H), 8.13 (dq, *J* = 8.5, 1.0 Hz, 1H), 7.84 (ddt, *J* = 8.1, 1.5, 0.6 Hz, 1H), 7.70 (ddd, *J* = 8.4, 6.9, 1.5 Hz, 1H), 7.67 – 7.60 (m, 2H), 7.56 (ddd, *J* = 8.1, 6.9, 1.2 Hz, 1H), 7.22 – 7.16 (m, 2H) ppm.

^{13}C NMR (125 MHz, $CDCl_3$, 23 °C): *δ* = 162.9 (d, *J* = 248.0 Hz), 149.7, 147.4, 134.0 (d, *J* = 3.5 Hz), 133.1, 132.9, 129.5, 129.2 (d, *J* = 18.2 Hz), 129.1, 128.0, 128.0, 127.2, 116.2 (d, *J* = 21.8 Hz) ppm.

^{19}F NMR (471 MHz, $CDCl_3$, 23 °C): *δ* = −114.1 (tt, *J* = 8.7, 5.3 Hz) ppm.

HRMS (EI): calc'd for $C_{15}H_{10}N_1F_1$ $[M]^+$: 223.0792, found: 223.0792; deviation: 0.0 ppm.

Aminoglutethimide-derived biphenyl 3o

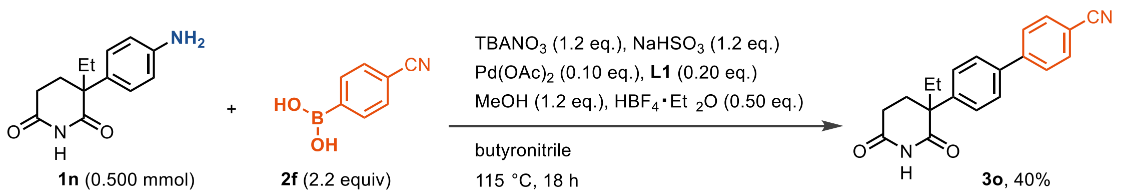

To a 4-mL vial, $TBANO_3$ (182 mg, 0.600 mmol, 1.20 equiv.), $NaHSO_3$ (62 mg, 0.600 mmol, 1.2 equiv.), $Pd(OAc)_2$ (12 mg, 0.050 mmol, 0.10 equiv.), **L1** (47 mg, 0.10 mmol, 0.20 equiv.), (4-cyanophenyl)boronic acid (**2f**) (162 mg, 1.10 mmol, 2.20 equiv.) and **1n** (116 mg, 0.500 mmol, 1.00 equiv.) were added followed by anhydrous *n*-PrCN (1.25 mL, c = 0.400 M) and MeOH (24.0 µL, 19.0 mg, 0.600 mmol, 1.20 equiv.). The suspension was stirred for 5 minutes at 23 °C and then $HBF_4·OEt_2$ (34 µL, 40 mg, 0.25 mmol, 0.50 equiv.) was added. The vial was sealed with a septum cap and the reaction mixture was heated at 115 °C and stirred (400 rpm) for 18 h. The reaction mixture was cooled to 23 °C and a sat. aq. $NaHCO_3$ (1 mL) solution was added, and the resulting mixture was extracted by EtOAc (3 × 2 mL). The combined organic phases were passed through a pad of silica gel (2 g), and the solvent was evaporated *in vacuo*. The crude material was purified by flash column chromatography (SiO_2, Hexane:EtOAc = 4:1) affording 63 mg (40% yield) of **3o** as an off-white solid.

R*f* = 0.55 (Hexane:EtOAc = 1:1).

NMR Spectroscopy:

^{1}H NMR (500 MHz, $CDCl_3$, 23 °C): 7.76 – 7.70 (m, 2H), 7.69 – 7.62 (m, 2H), 7.62 – 7.56 (m, 2H), 7.43 – 7.37 (m, 2H), 2.70 – 2.55 (m, 1H), 2.49 – 2.38 (m, 2H), 2.28 (m, 1H), 2.10 (dq, *J* = 14.7, 7.4 Hz, 1H), 1.96 (dq, *J* = 14.7, 7.4 Hz, 1H), 0.91 (t, *J* = 7.4 Hz, 3H) ppm.

^{13}C NMR (125 MHz, $CDCl_3$, 23 °C): *δ* = 174.9, 172.2, 144.6, 139.4, 138.4, 132.6, 127.8, 127.6, 127.0, 118.8, 111.2, 51.0, 32.8, 29.2, 27.0, 9.0 ppm.

HRMS (EI): calc'd for $C_{20}H_{18}N_2O_2$ $[M]^+$: 318.1362, found: 318.1365; deviation: +0.9 ppm.

Darunavir-derived biphenyl 3p

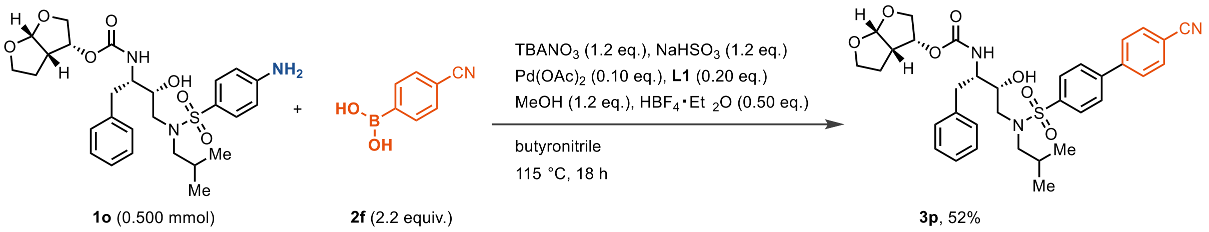

To a 4-mL vial, $TBANO_3$ (182 mg, 0.600 mmol, 1.20 equiv.), $NaHSO_3$ (62 mg, 0.600 mmol, 1.2 equiv.), $Pd(OAc)_2$ (12 mg, 0.050 mmol, 0.10 equiv.), **L1** (47 mg, 0.10 mmol, 0.20 equiv.), (4-cyanophenyl)boronic acid (**2f**) (162 mg, 1.10 mmol, 2.20 equiv.) and Darunavir (**1n**) (274 mg, 0.500 mmol, 1.00 equiv.) were added followed by anhydrous *n*-PrCN (1.25 mL, c = 0.400 M) and MeOH (24.0 µL, 19.0 mg, 0.600 mmol, 1.20 equiv.). The suspension was stirred for 5 minutes at 23 °C and then $HBF_4·OEt_2$ (34 µL, 40 mg, 0.25 mmol, 0.50 equiv.) was added. The vial was sealed with a septum cap and the reaction mixture was heated at 115 °C and stirred (400 rpm) for 18 h. The reaction mixture was cooled to 23 °C and a sat. aq. $NaHCO_3$ (1 mL) solution was added, and the resulting mixture was extracted by EtOAc (3 × 2 mL). The combined organic phases were passed through a pad of silica gel (2 g), and the solvent was evaporated *in vacuo*. The crude material was purified by flash column chromatography (SiO_2, Hexane:EtOAc = 1:1) affording 167 mg (52 % yield) of **3p** as a colorless solid.

R*f* = 0.39 (Hexane:EtOAc = 1:1).

NMR Spectroscopy:

^{1}H NMR (500 MHz, $CDCl_3$, 23 °C): δ = 7.94 – 7.88 (m, 2H), 7.84 – 7.79 (m, 2H), 7.77 – 7.71 (m, 4H), 7.35 – 7.27 (m, 2H), 7.28 – 7.21 (m, 3H), 5.67 (d, J = 5.2 Hz, 1H), 5.04 (q, J = 6.7 Hz, 1H), 4.94 (d, J = 8.6 Hz, 1H), 4.01 – 3.84 (m, 4H), 3.78 – 3.63 (m, 3H), 3.25 (dd, J = 15.2, 8.3 Hz, 1H), 3.19 – 3.03 (m, 3H), 3.02 – 2.80 (m, 3H), 1.96 – 1.81 (m, 1H), 1.78 – 1.61 (m, 1H), 1.55 – 1.47 (m, 1H), 0.98 (d, J = 6.6 Hz, 3H), 0.93 (d, J = 6.6 Hz, 3H) ppm.

^{13}C NMR (125 MHz, $CDCl_3$, 23 °C): δ = 155.6, 143.6, 143.5, 138.4, 137.7, 132.9, 129.4, 128.6, 128.2, 128.1, 128.0, 126.6, 118.5, 112.3, 109.4, 73.5, 72.9, 71.0, 69.6, 58.6, 55.3, 53.6, 45.5, 35.7, 27.2, 25.9, 20.2, 19.9 ppm.

HRMS (ESI): calc'd for $C_{34}H_{39}O_7N_3Na_1S_1$ $[M+Na]^+$: 656.2400, found: 656.2401; deviation: +0.2 ppm.

Unsuccessful examples

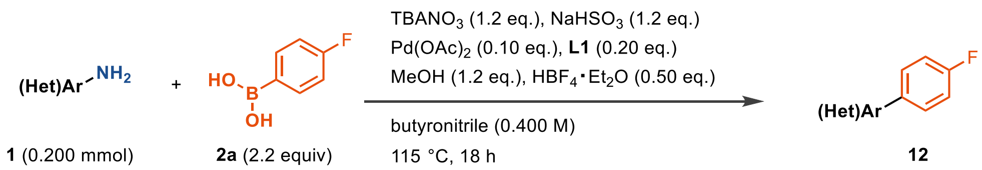

Procedure

To a 4-mL vial, $TBANO_3$ (73 mg, 0.24 mmol, 11.2 equiv.), $NaHSO_3$ (25 mg, 0.24 mmol, 1.2 equiv.), $Pd(OAc)_2$ (4.6 mg, 0.020 mmol, 0.10 equiv.), **L1** (18.6 mg, 0.040 mmol, 0.20 equiv.), **2a** (62 mg, 0.44 mmol, 2.2 equiv.) and aniline (0.200 mmol) if solid were added followed by anhydrous *n*-PrCN (0.50 mL, c = 0.40 M), MeOH (24 µL, 19 mg, 0.24 mmol, 1.2 equiv.) and aniline (0.200 mmol) if liquid. The suspension was stirred for 5 minutes at 23 °C and then $HBF_4{\cdot}OEt_2$ (14 µL, 16 mg, 0.10 mmol, 0.5 equiv.) was added. The vial was sealed with a septum cap and the reaction mixture was heated at 115 °C and stirred (400 rpm) for 18 h. The reaction mixture was cooled to 23 °C, a sat. aq. $NaHCO_3$ (1 mL) solution was added and the resulting mixture was extracted by EtOAc (3 × 2 mL). The combined organic phases were passed through a pad of silica gel (2 g) and the solvent was evaporated *in vacuo*. The crude material was dissolved in $CDCl_3$ (0.5 mL) and α,α,α-trifluorotoluene (8.2 µL, 9.8 mg, 0.067 mmol) was added as internal standard. A reaction was deemed unsuccessful if in ^{19}F NMR spectroscopy (298 K, 471 MHz) no peak in the −100 ppm to −200 ppm range had an integration ratio to the peak −62.9 ppm (3F, s) corresponding to the internal standard of 0.2. This meant that the product (**12a-o**) had a yield lower than 20 %

List of unsuccessful examples:

The following reactions were deemed unsuccessful as the yield of the resulting biaryl measured by NMR was under 20 %. Due to the low yields of these reactions, isolation and characterization of the biaryls were not attempted. However, we would like to show these representative unsuccessful examples to show the limitations of this reaction.

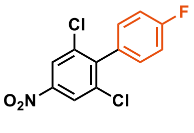

12a

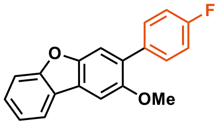

12b

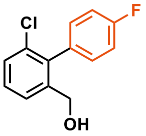

12c

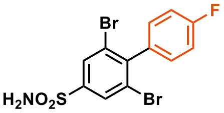

12d

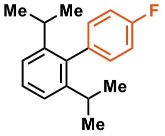

12e

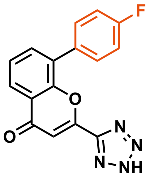

12f

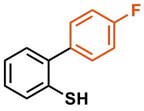

12g

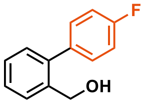

12h

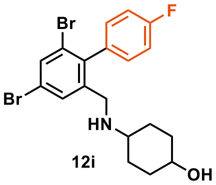

12i

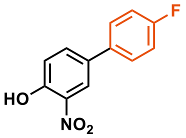

12j

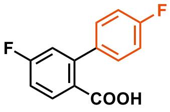

12k

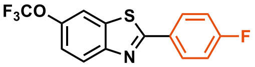

12l

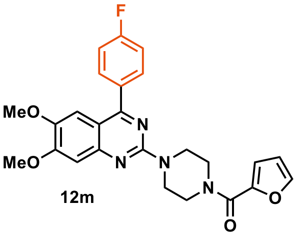

12m

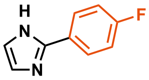

12n

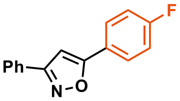

12o

Fig S22. Unsuccessful substrates

SPECTROSCOPIC DATA

^{1}H NMR spectrum of 3b

CDCl$_3$, 500 MHz, 23 ºC

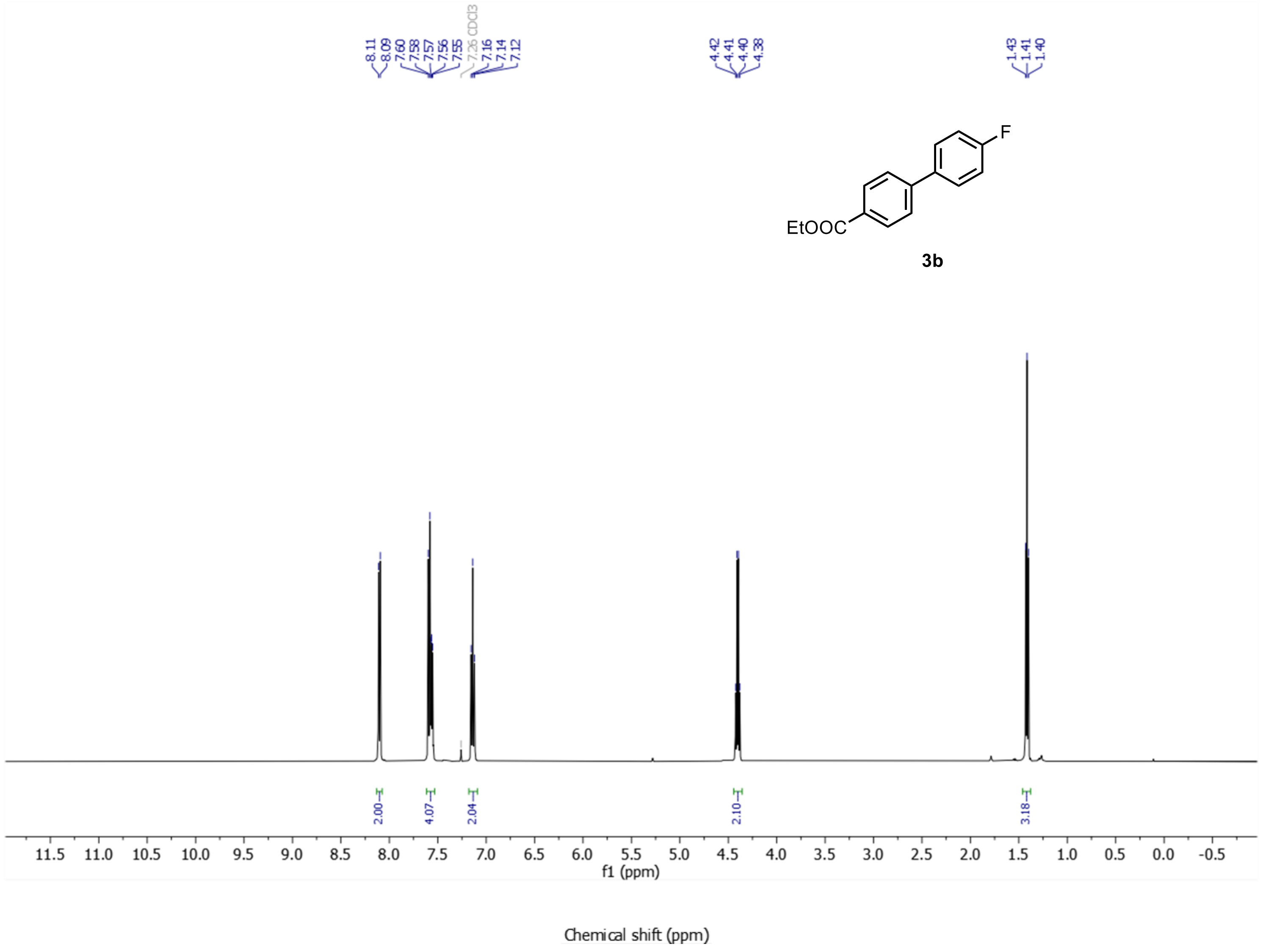

^{13}C NMR spectrum of 3b

$CDCl_3$, 125 MHz, 23 ºC

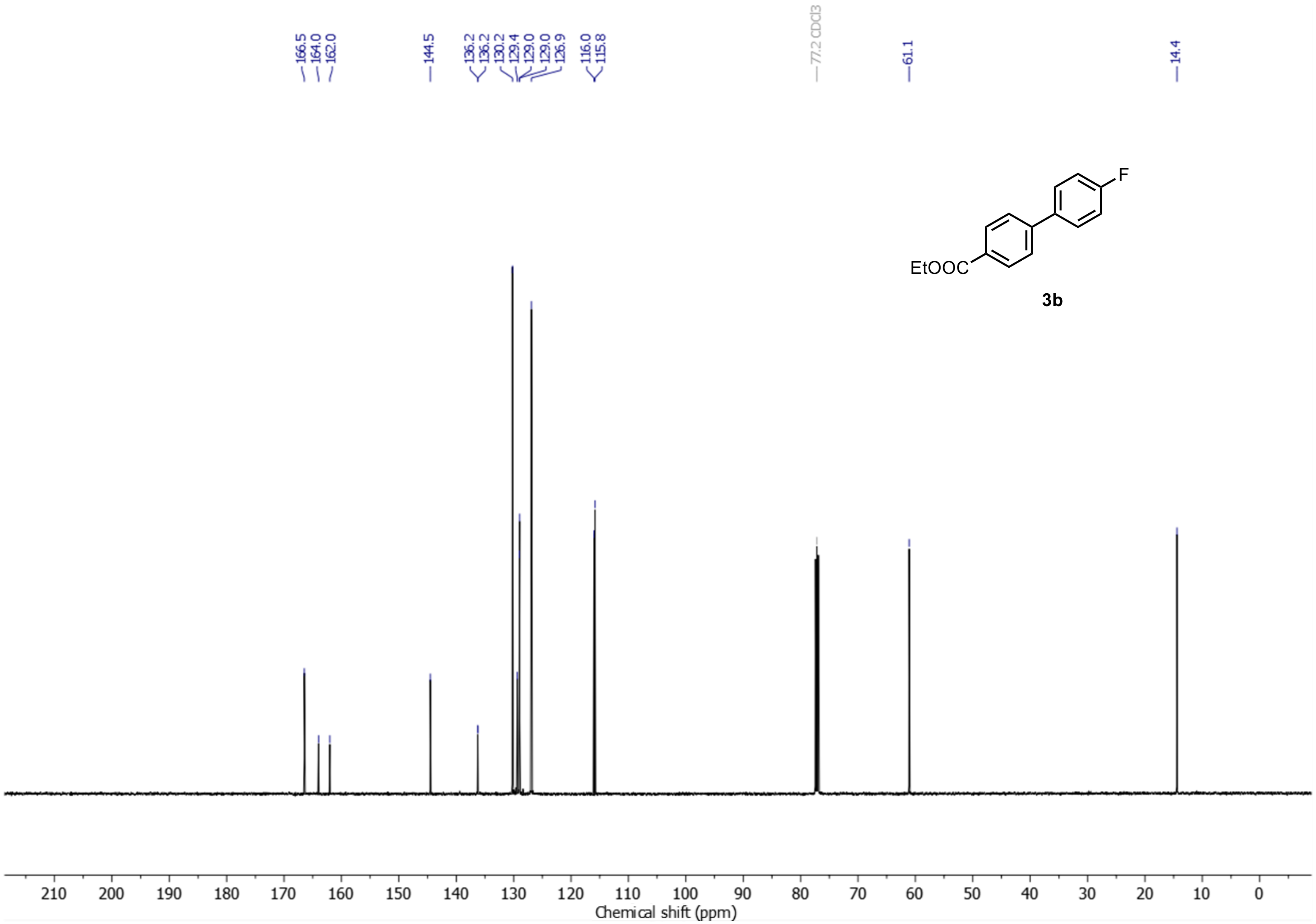

^{19}F NMR spectrum of 3b

$CDCl_3$, 471 MHz, 23 ºC

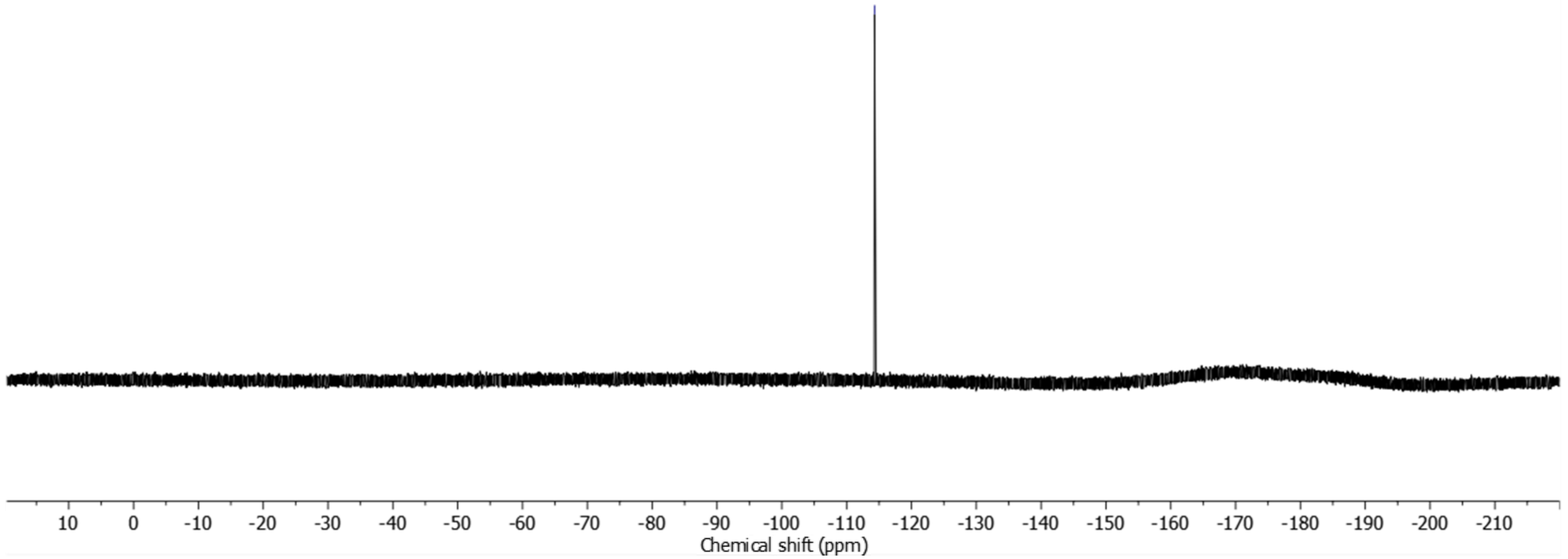

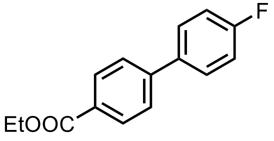

3b

^{1}H NMR spectrum of 3c

$CDCl_3$, 500 MHz, 23 ºC

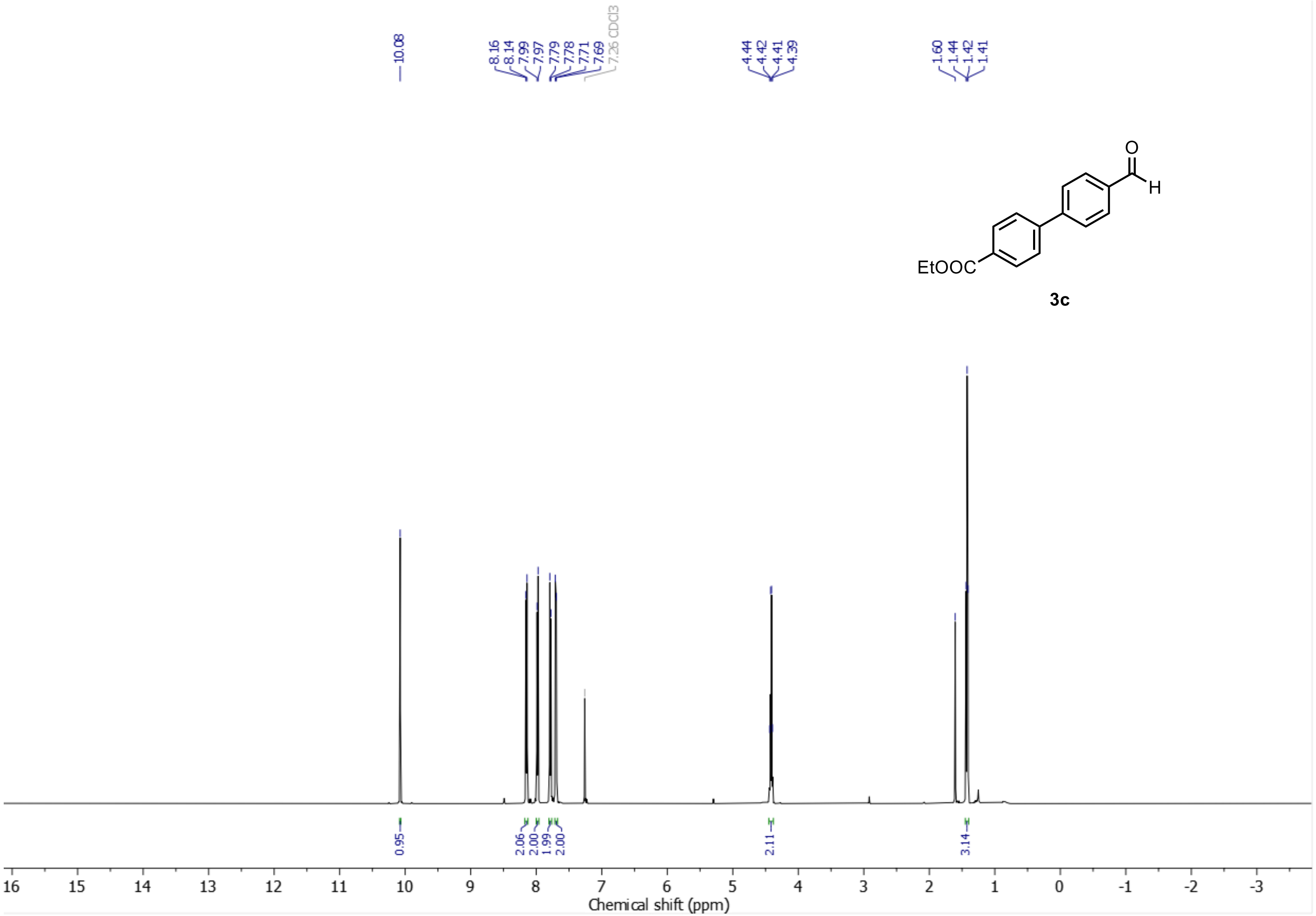

^{13}C NMR spectrum of 3c

$CDCl_3$, 125 MHz, 23 ºC

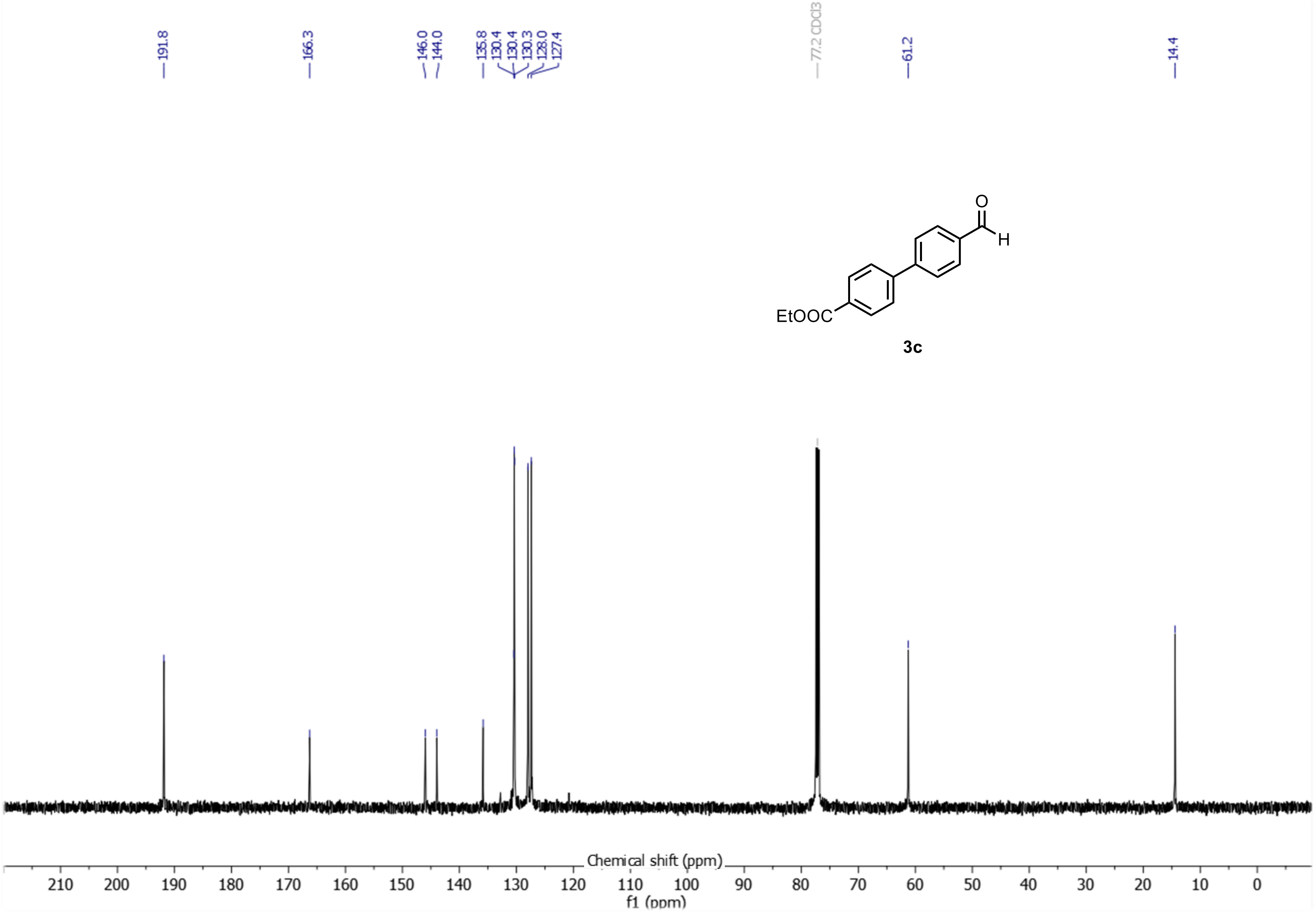

^{1}H NMR spectrum of 3d

$CDCl_3$, 500 MHz, 23 ºC

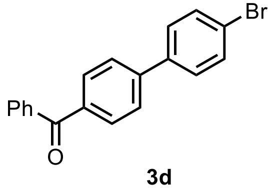

3d

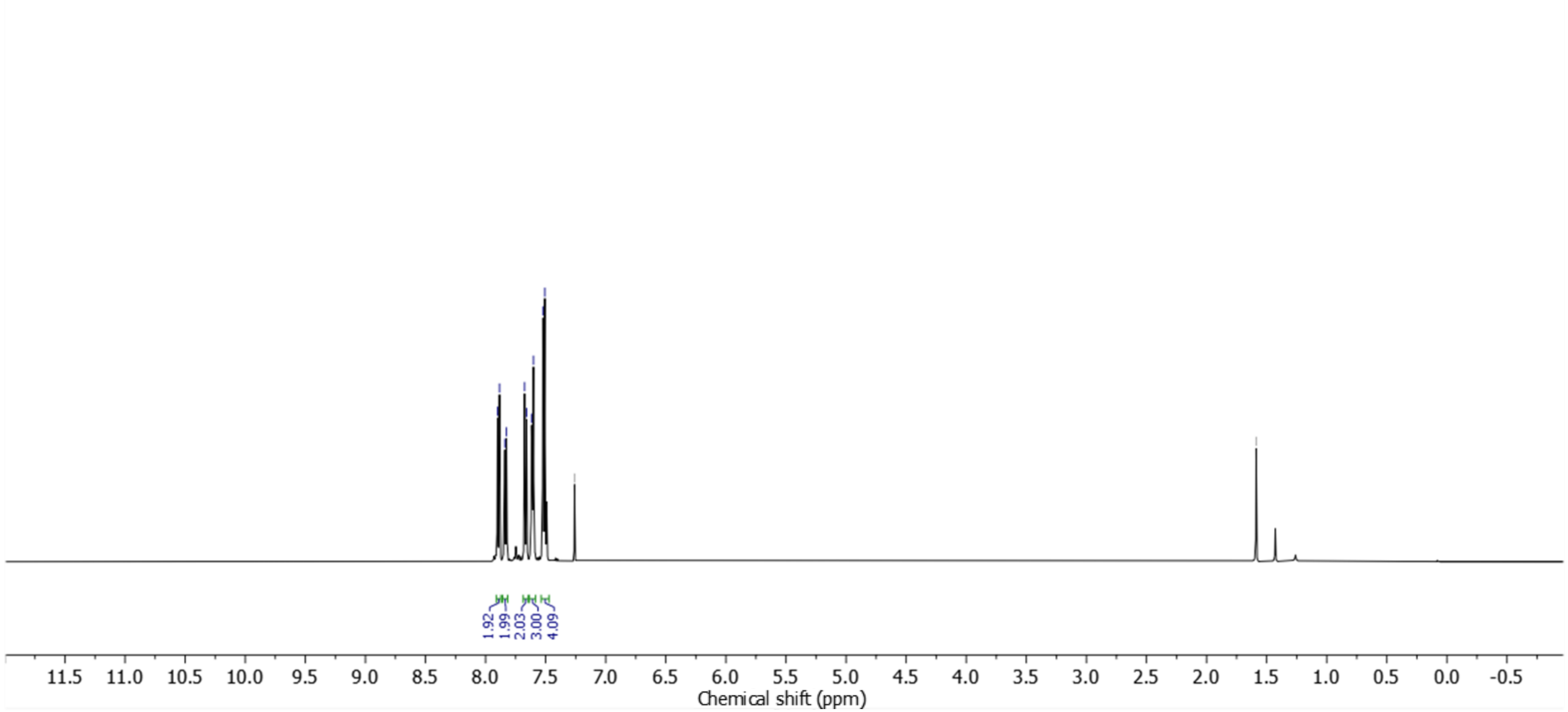

^{13}C NMR spectrum 3d

$CDCl_3$, 125 MHz, 23 ºC

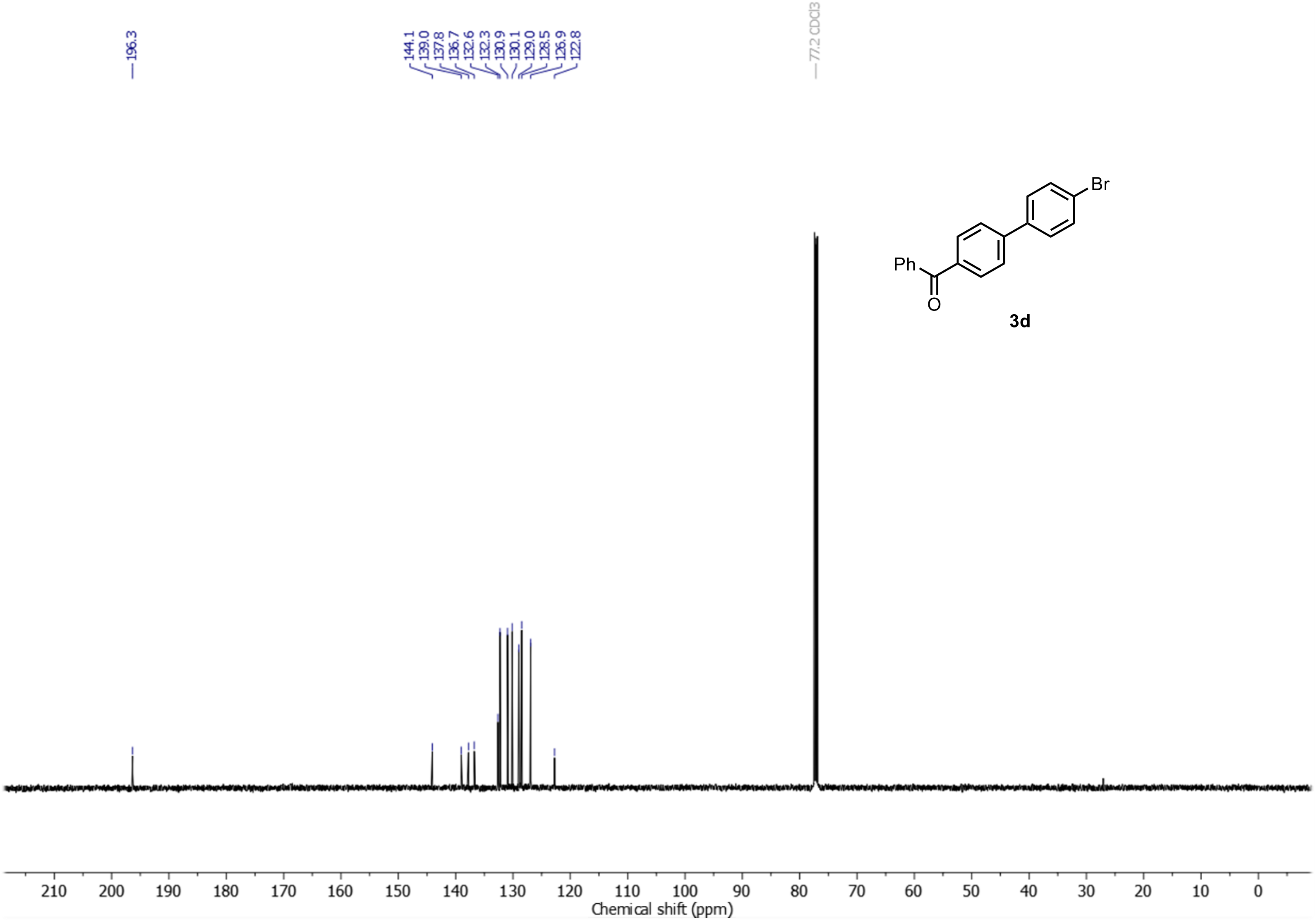

3d

^{1}H NMR spectrum of 3e

$CDCl_3$, 500 MHz, 23 ºC

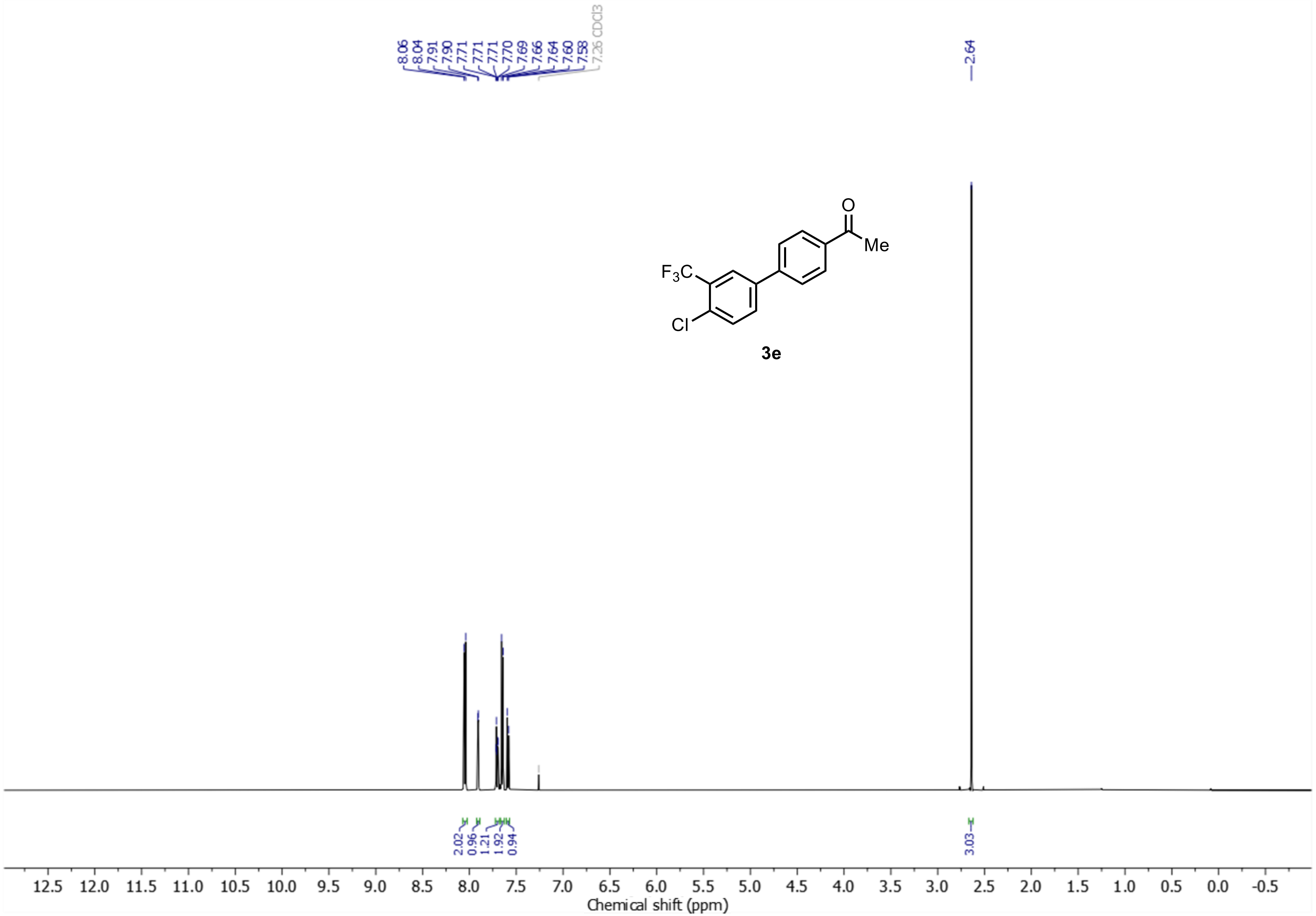

^{13}C NMR spectrum of 3e

$CDCl_3$, 125 MHz, 23 ºC

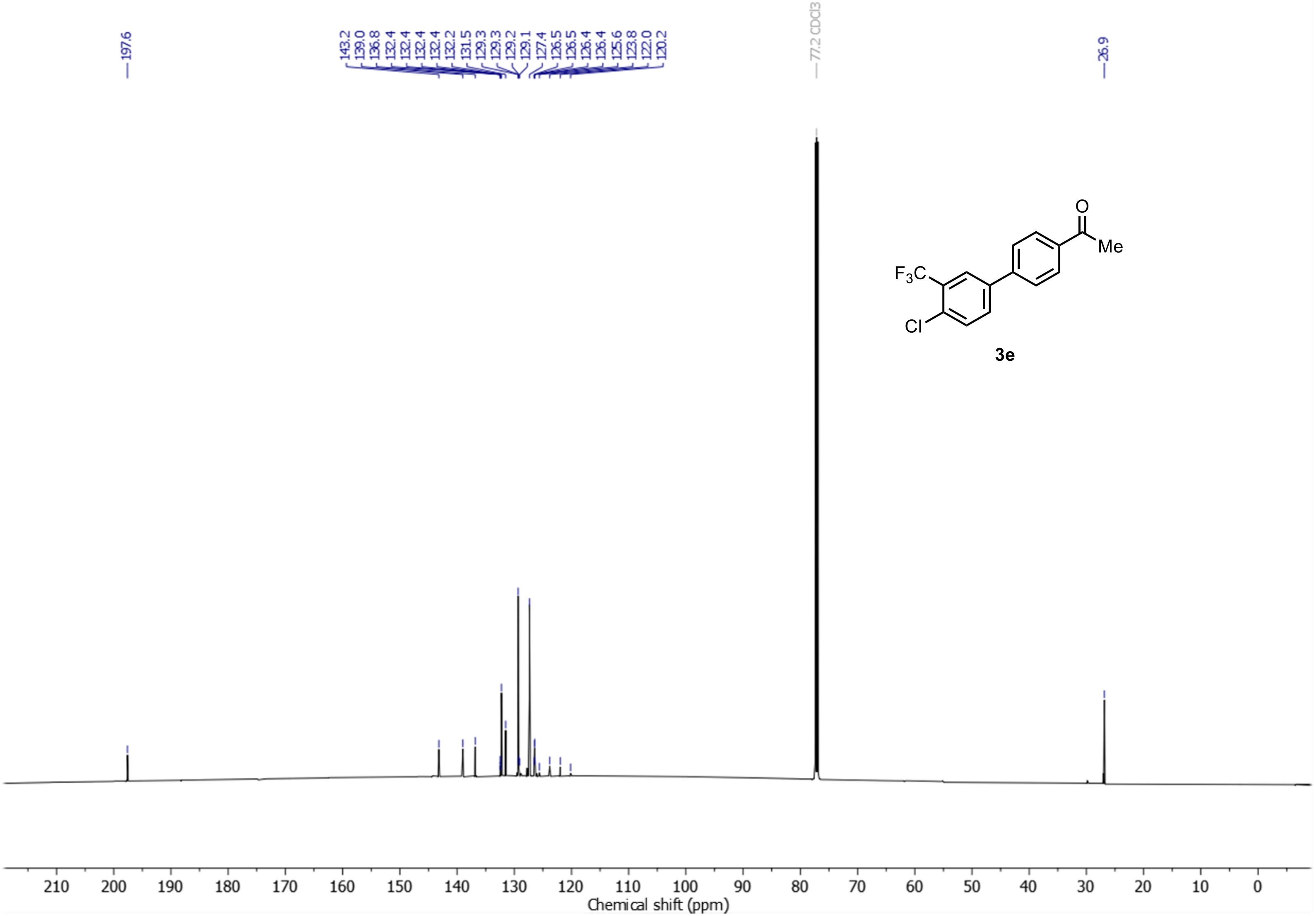

3e

^{19}F NMR spectrum of 3e

$CDCl_3$, 471 MHz, 23 ºC

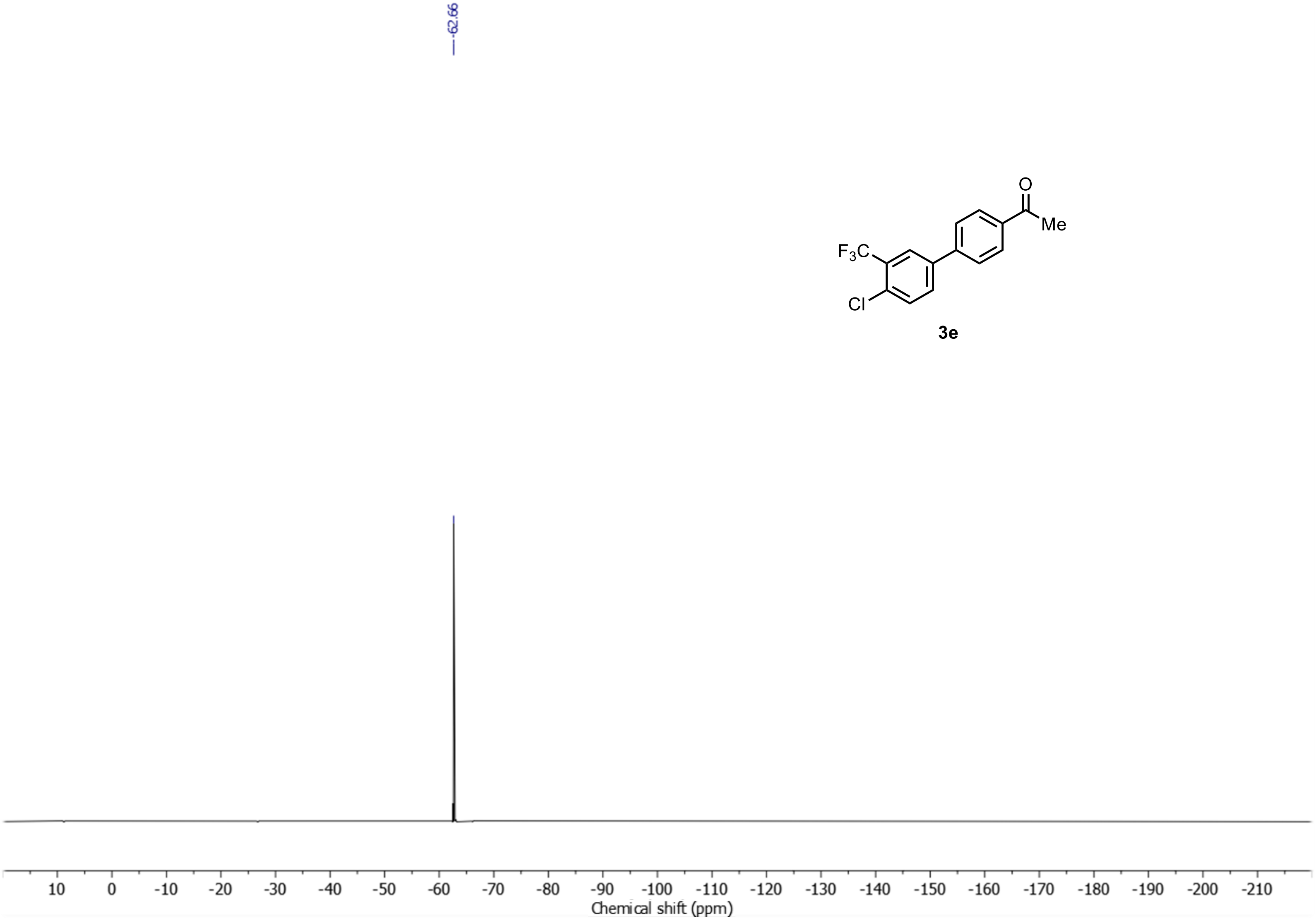

3e

^{1}H NMR spectrum of 3f

$CDCl_3$, 500 MHz, 23 ºC

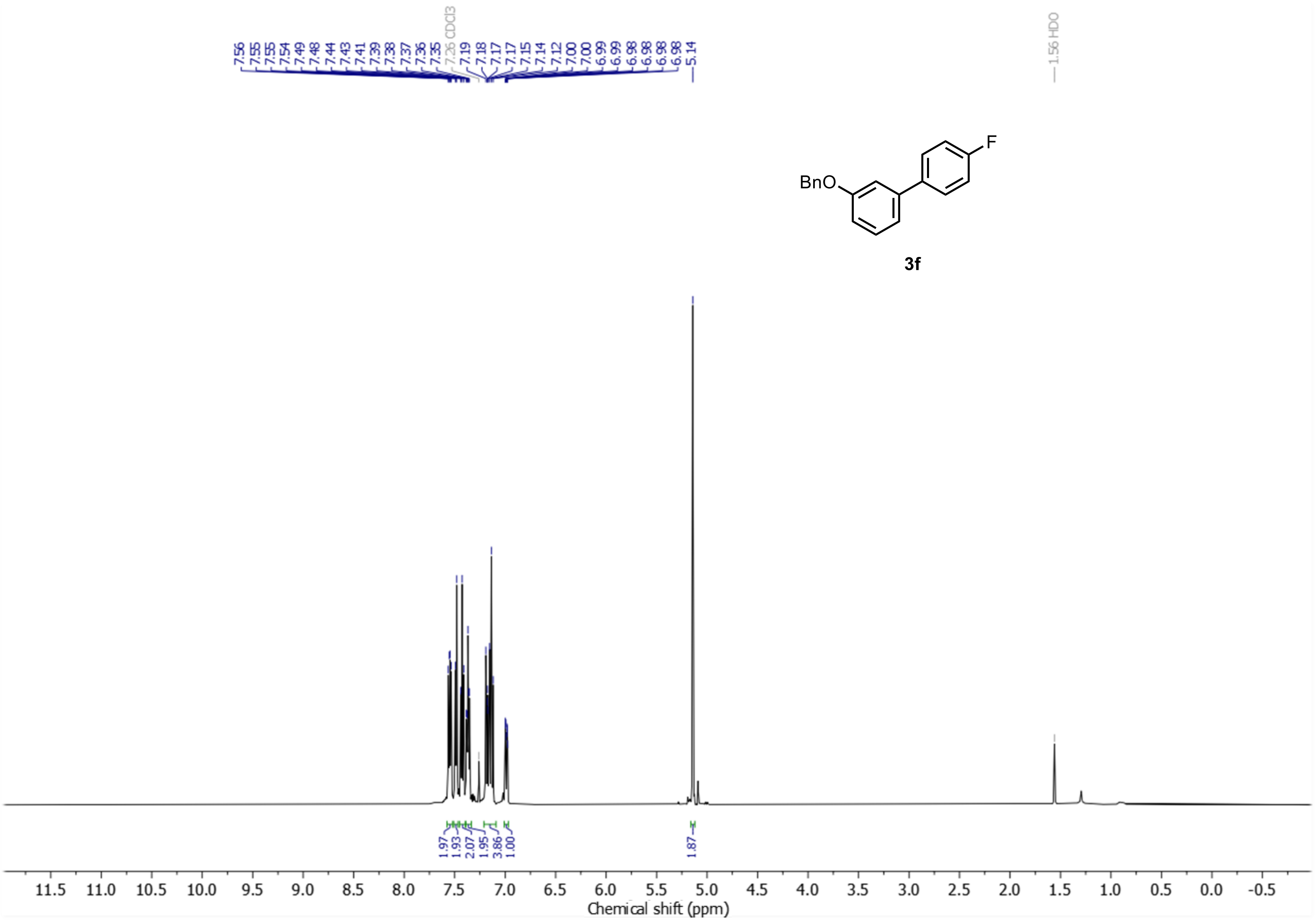

^{13}C NMR spectrum of 3f

$CDCl_3$, 125 MHz, 23 ºC

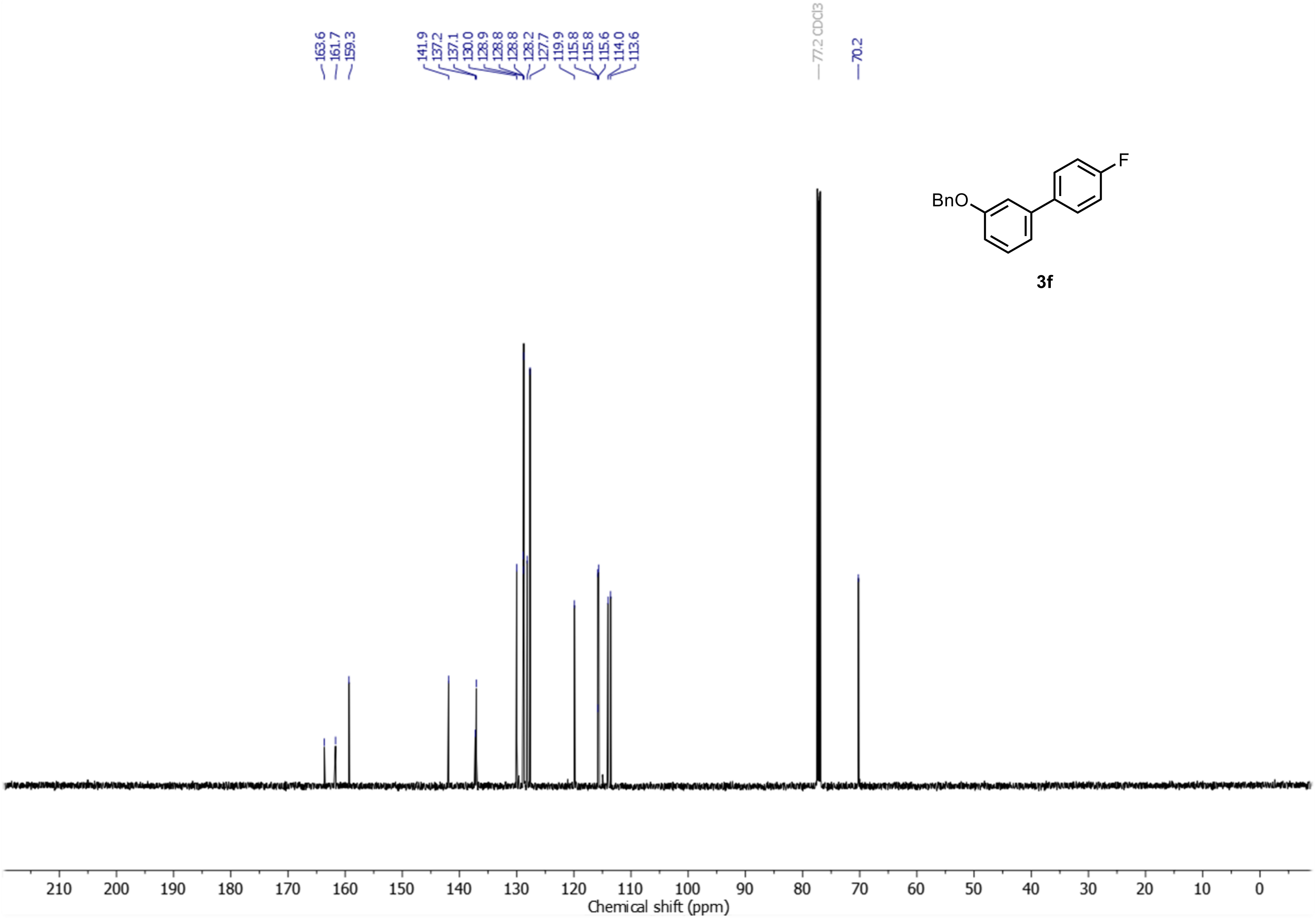

^{19}F NMR spectrum of 3f

$CDCl_3$, 471 MHz, 23 ºC

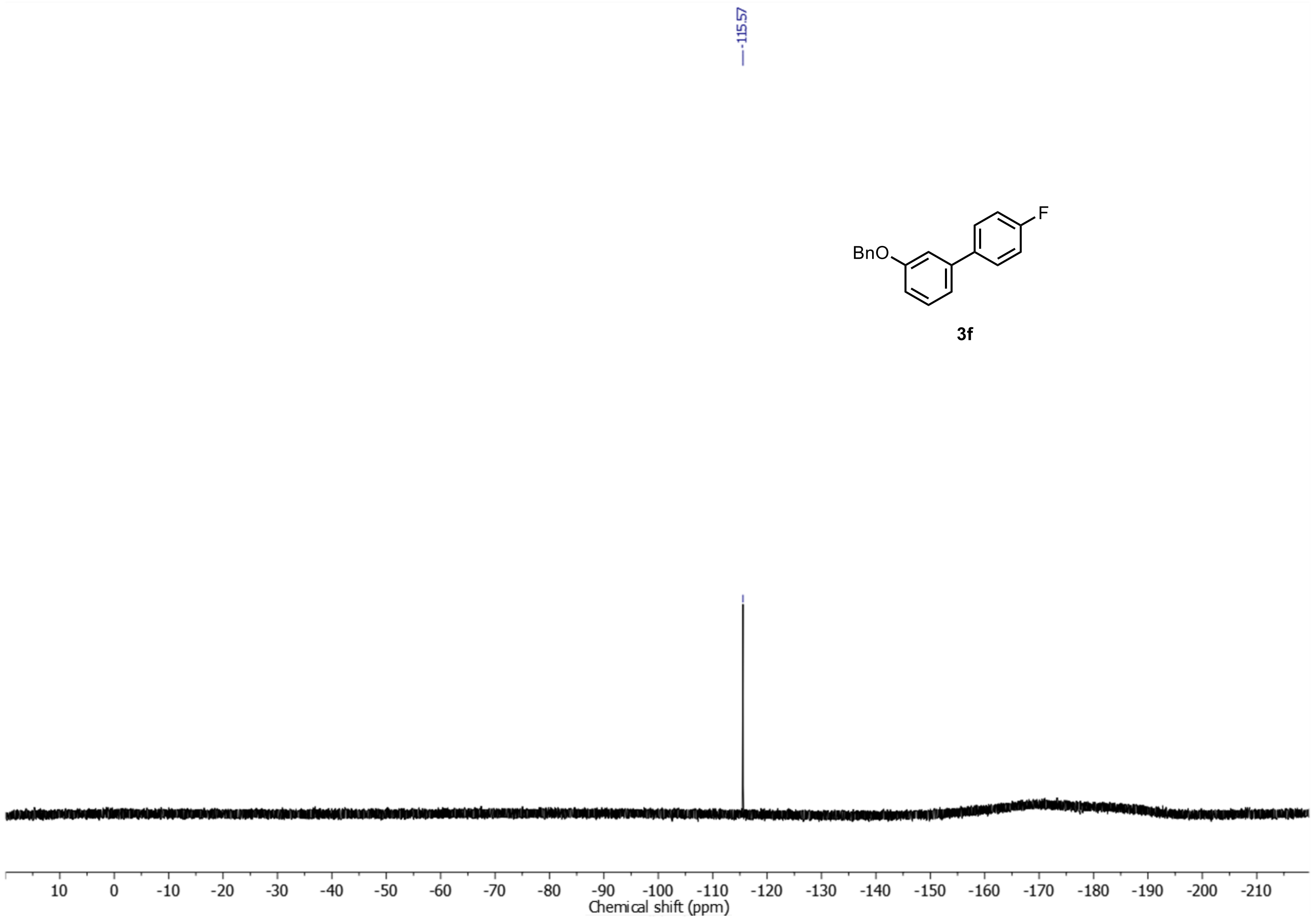

^{1}H NMR spectrum of 3g

$CDCl_3$, 500 MHz, 23 ºC

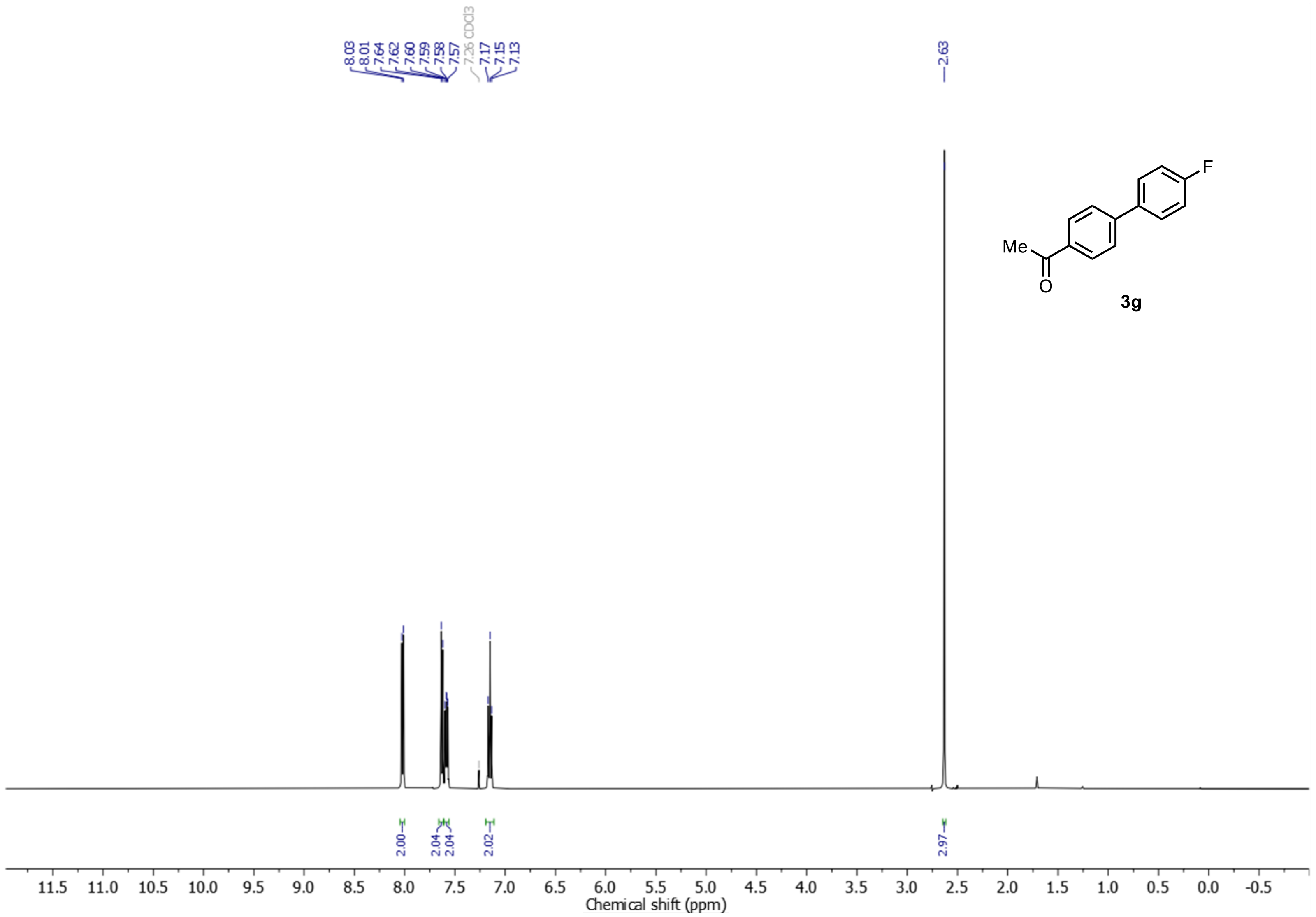

^{13}C NMR spectrum of 3g

$CDCl_3$, 500 MHz, 23 ºC

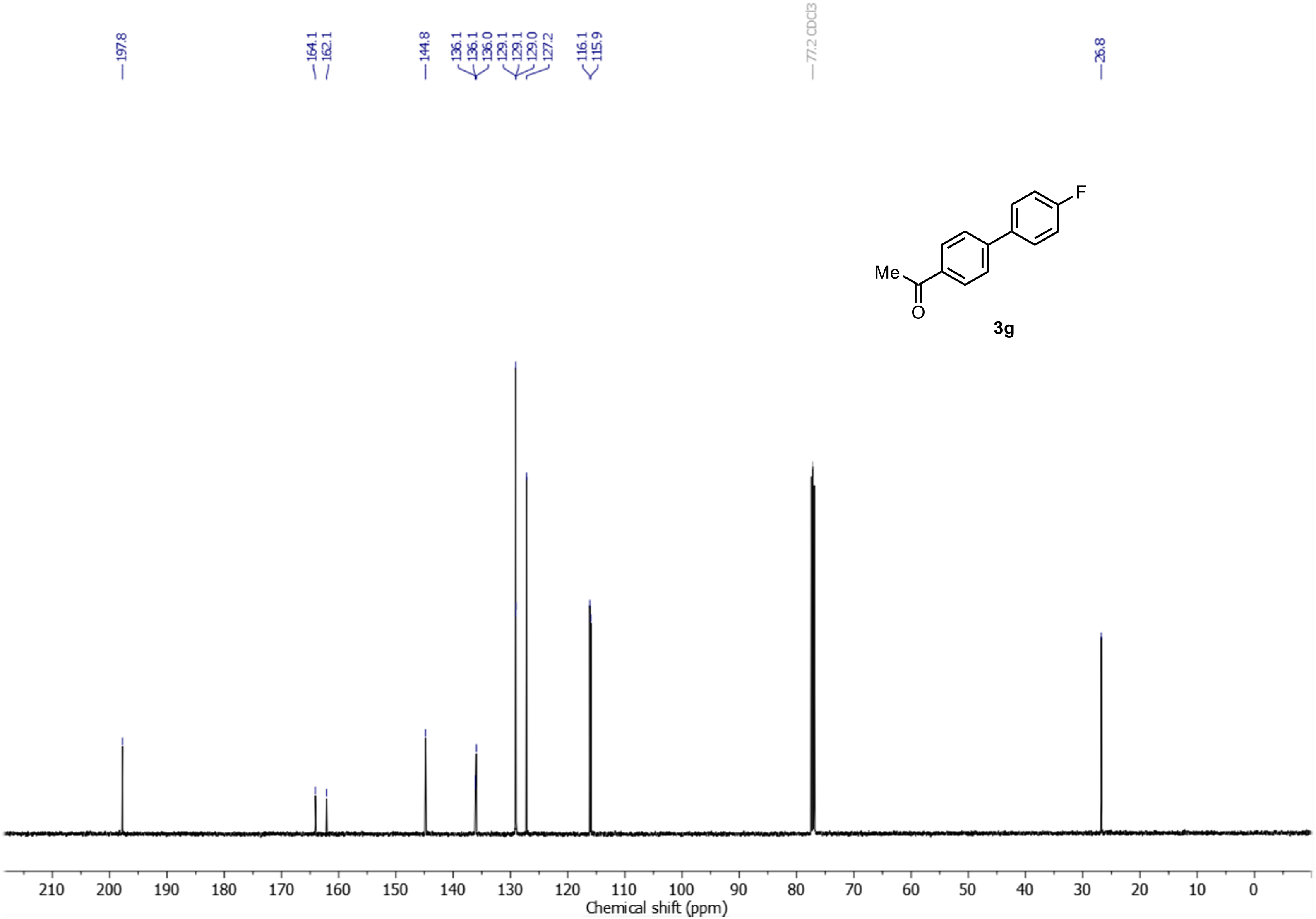

^{19}F NMR spectrum of 3g

$CDCl_3$, 471 MHz, 23 ºC

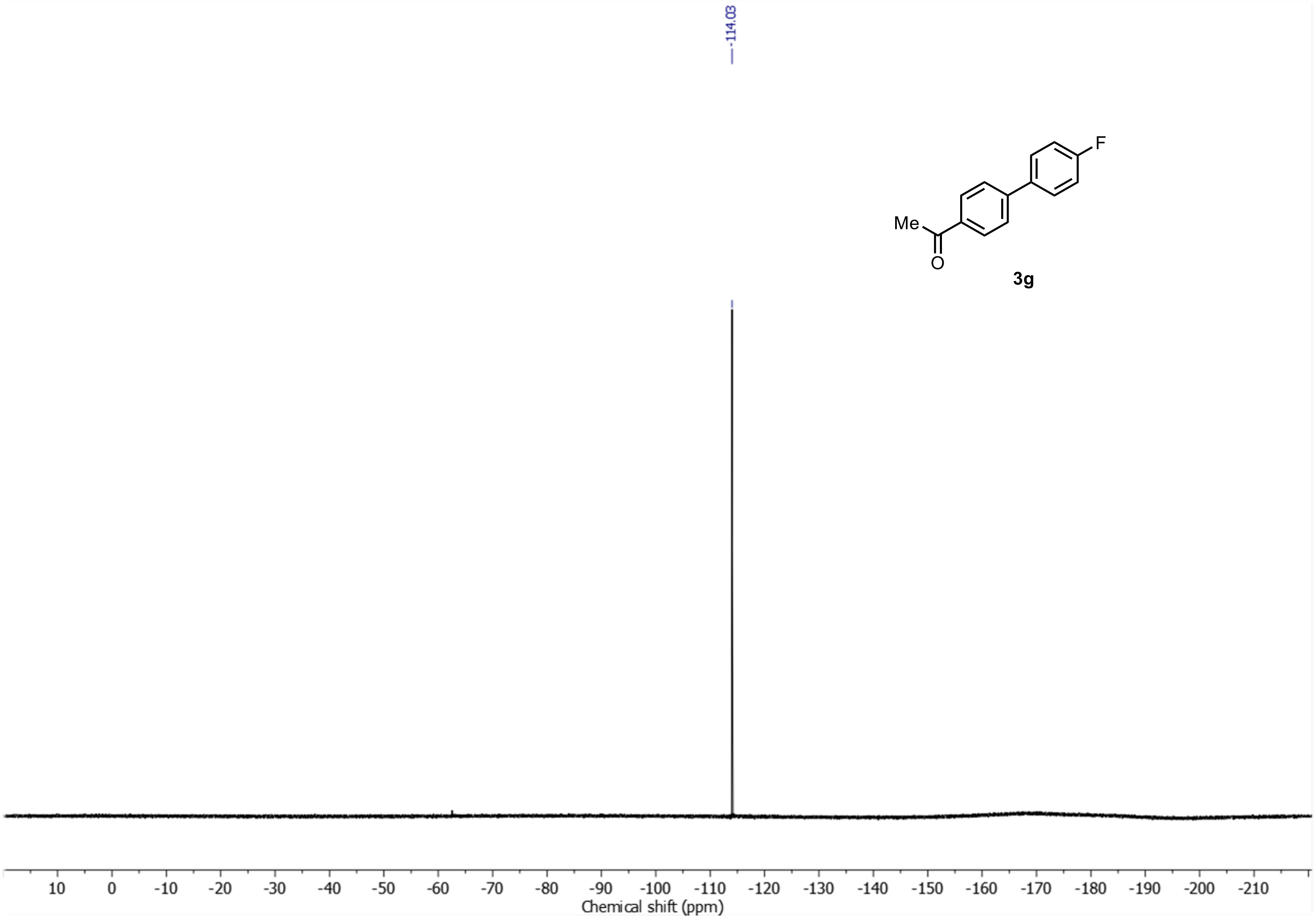

^{1}H NMR spectrum of 3h

$CDCl_3$, 500 MHz, 23 ºC

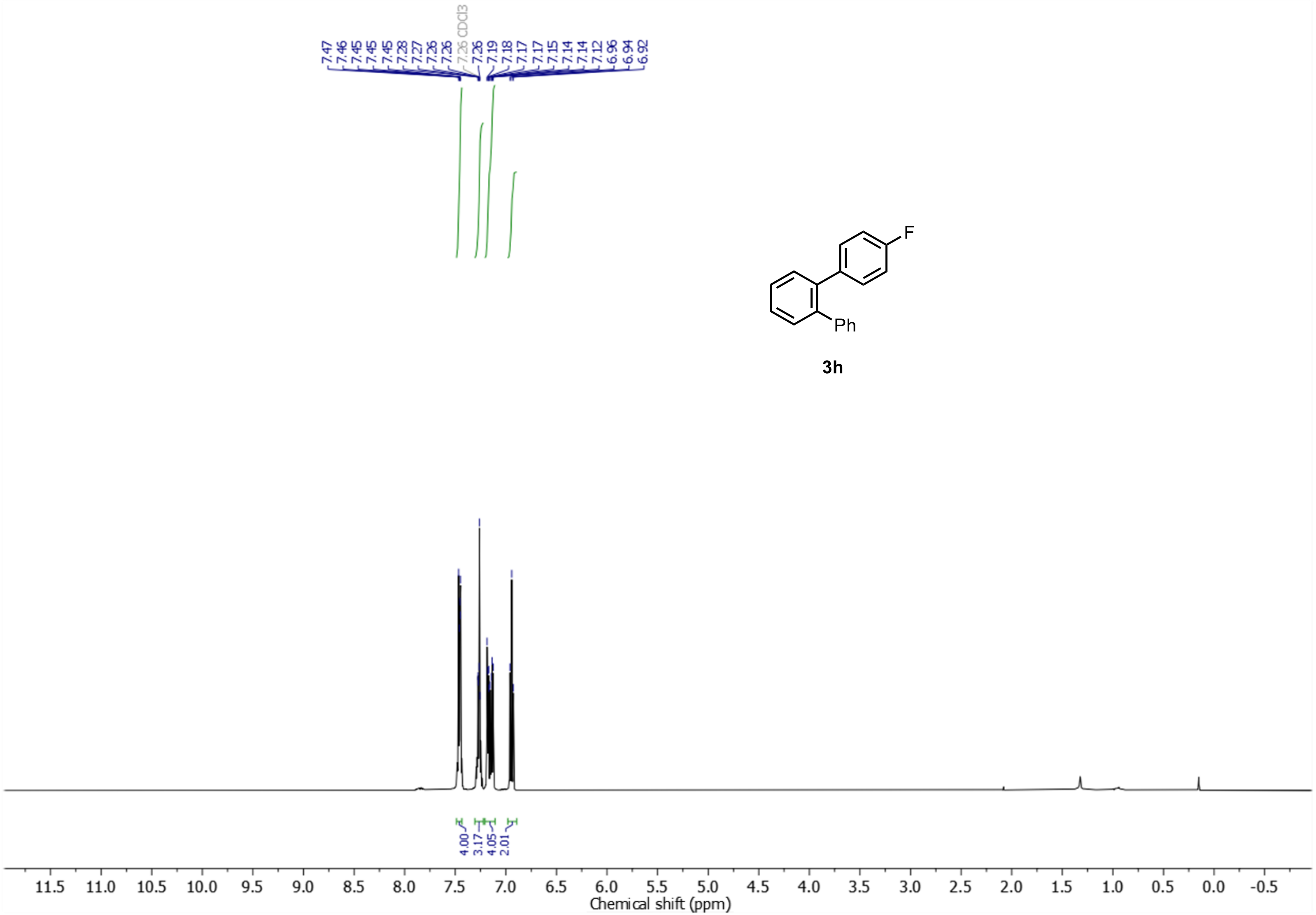

^{13}C NMR spectrum of 3h

CDCl$_3$, 125 MHz, 23 ºC

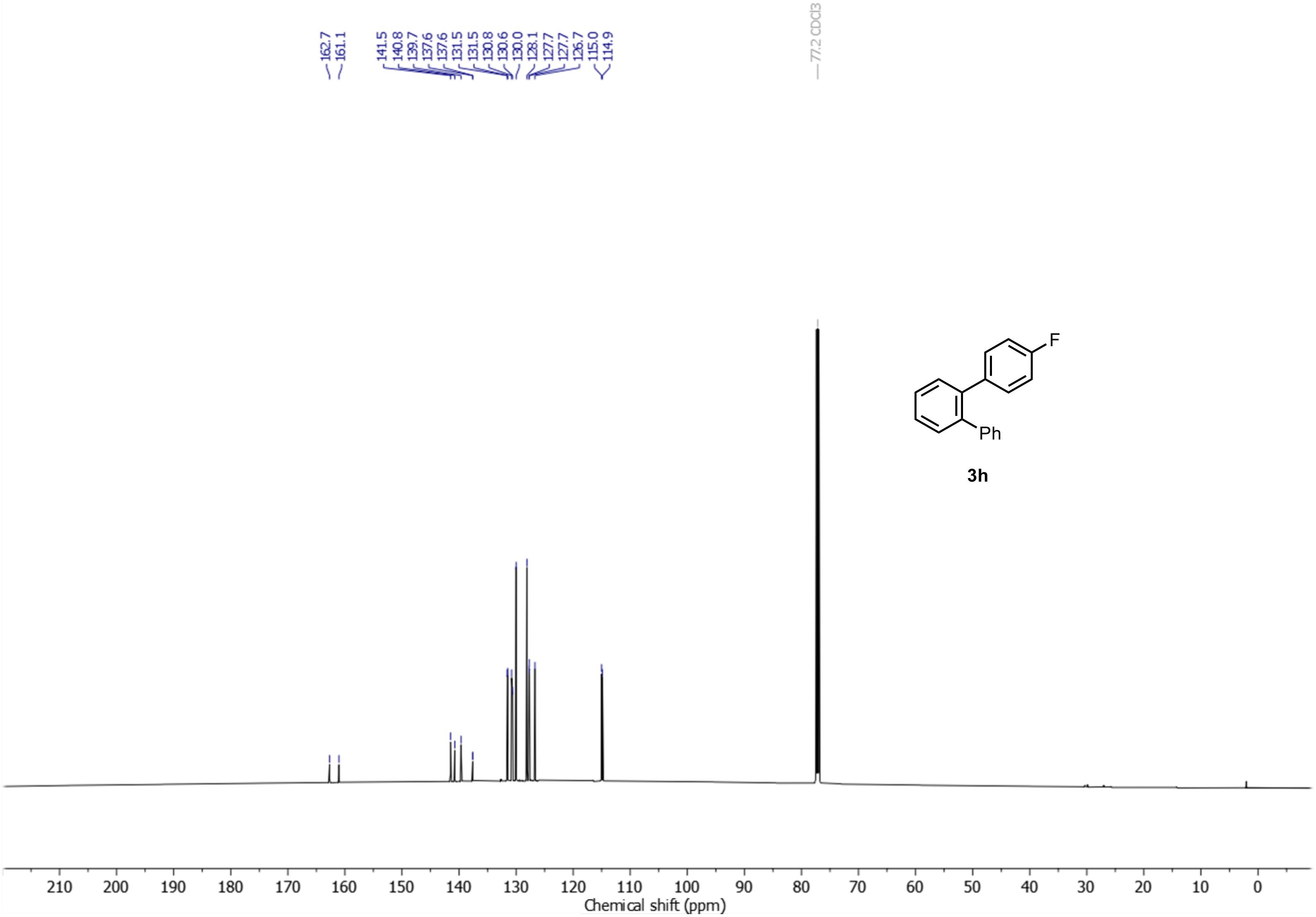

^{1}H NMR spectrum of 3i

$CDCl_3$, 500 MHz, 23 ºC

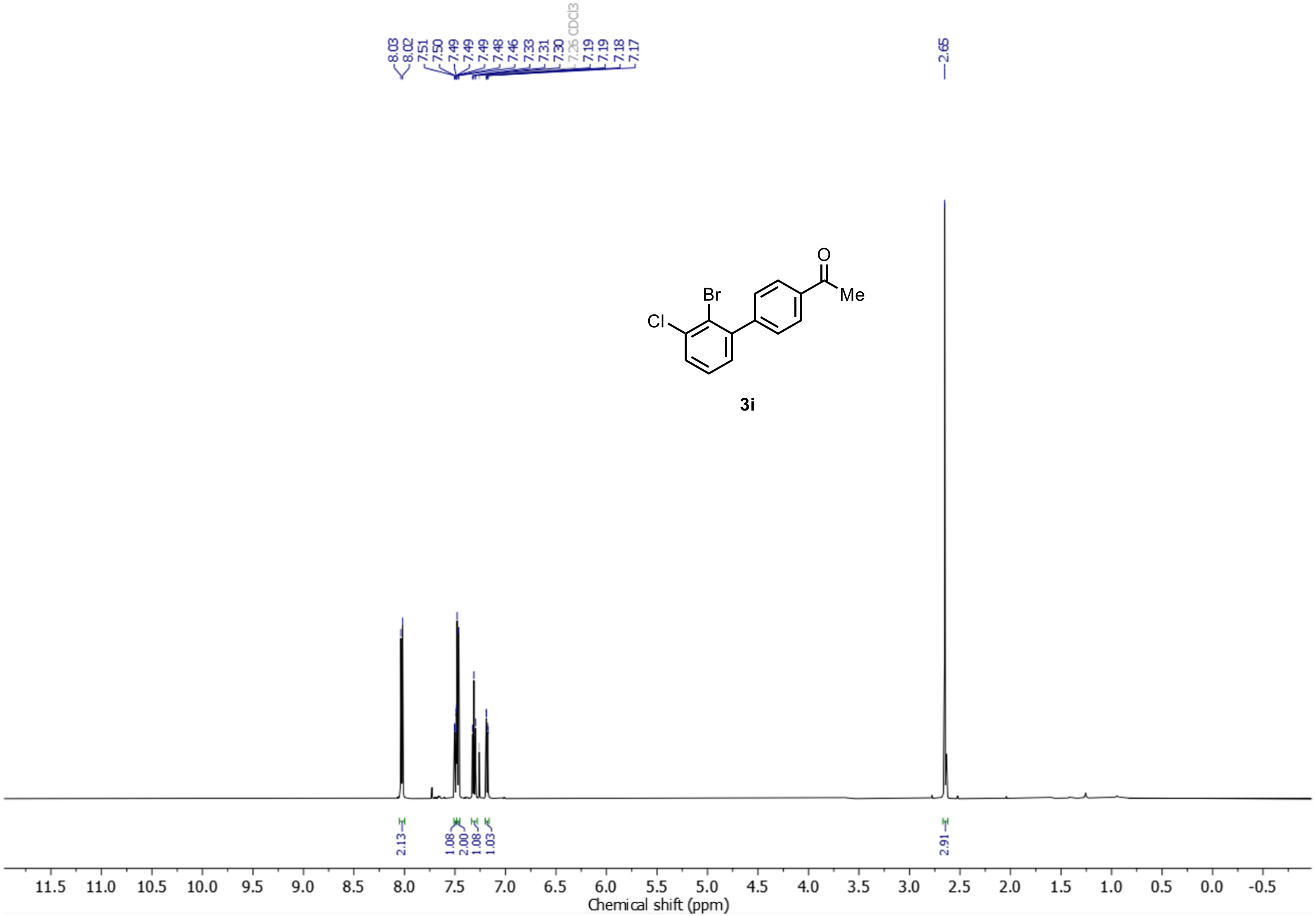

^{13}C NMR spectrum of 3i

$CDCl_3$, 125 MHz, 23 ºC

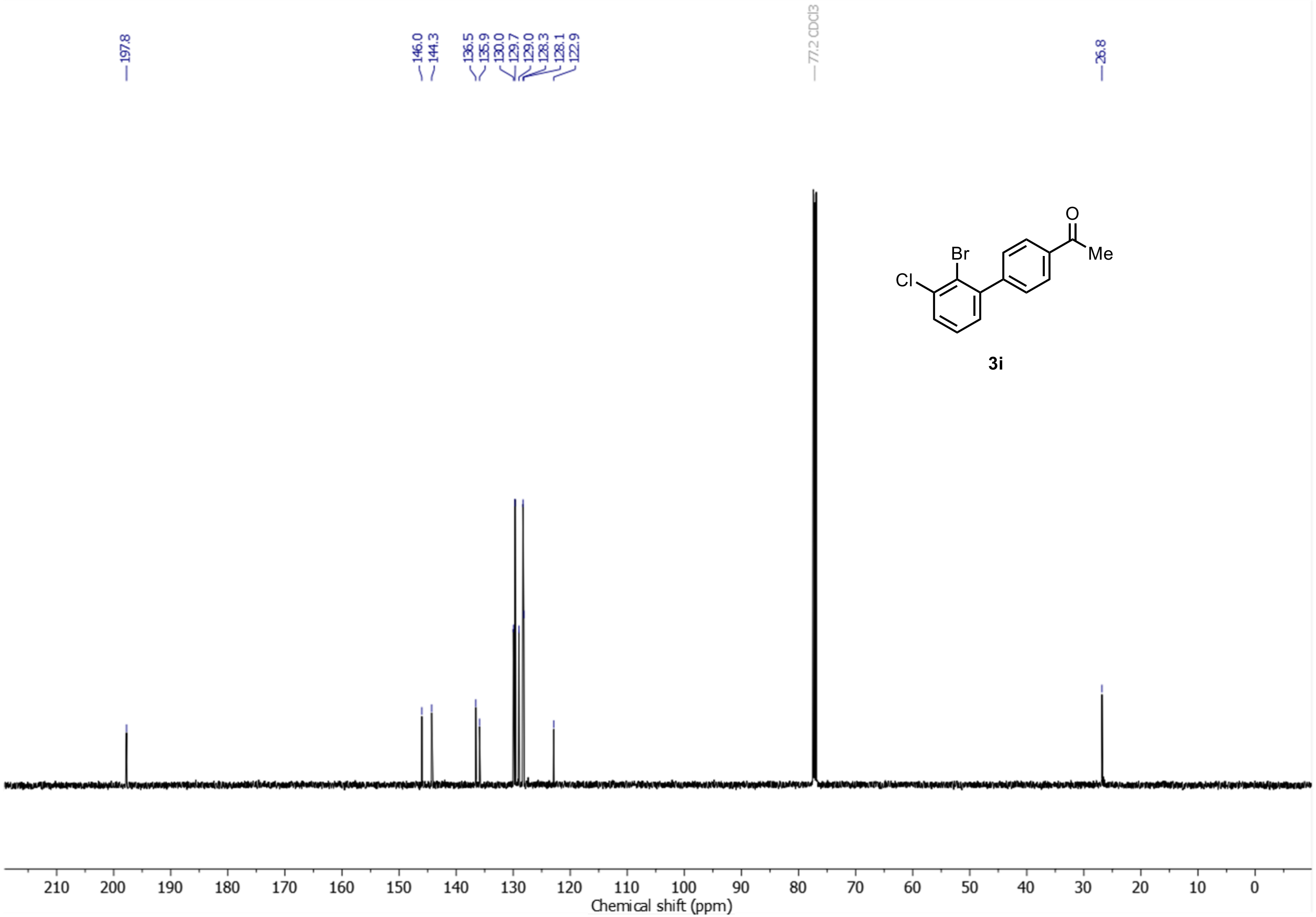

^{1}H NMR spectrum of 3j

$CDCl_3$, 500 MHz, 23 ºC

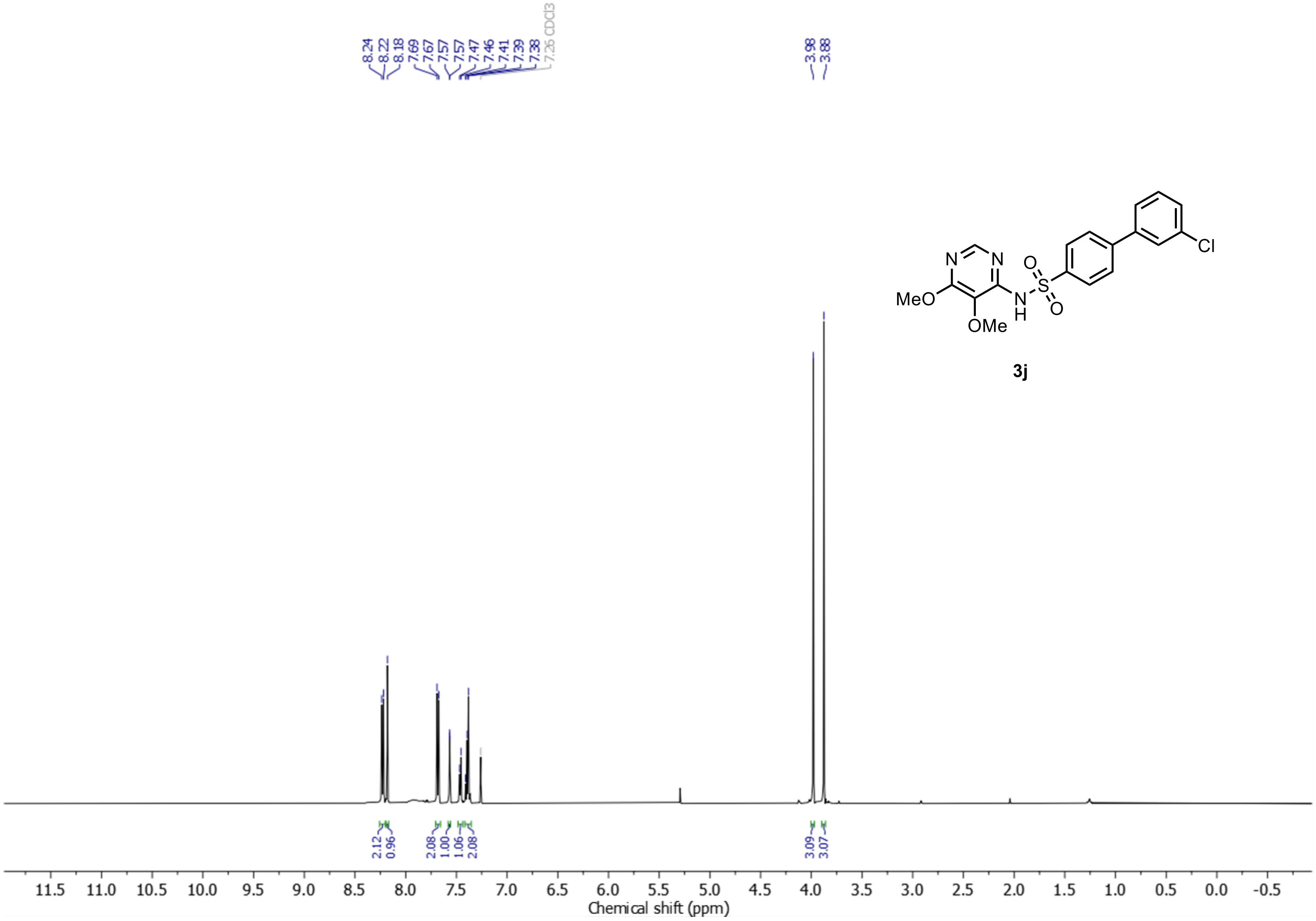

^{13}C NMR spectrum of 3j

$CDCl_3$, 125 MHz, 23 ºC

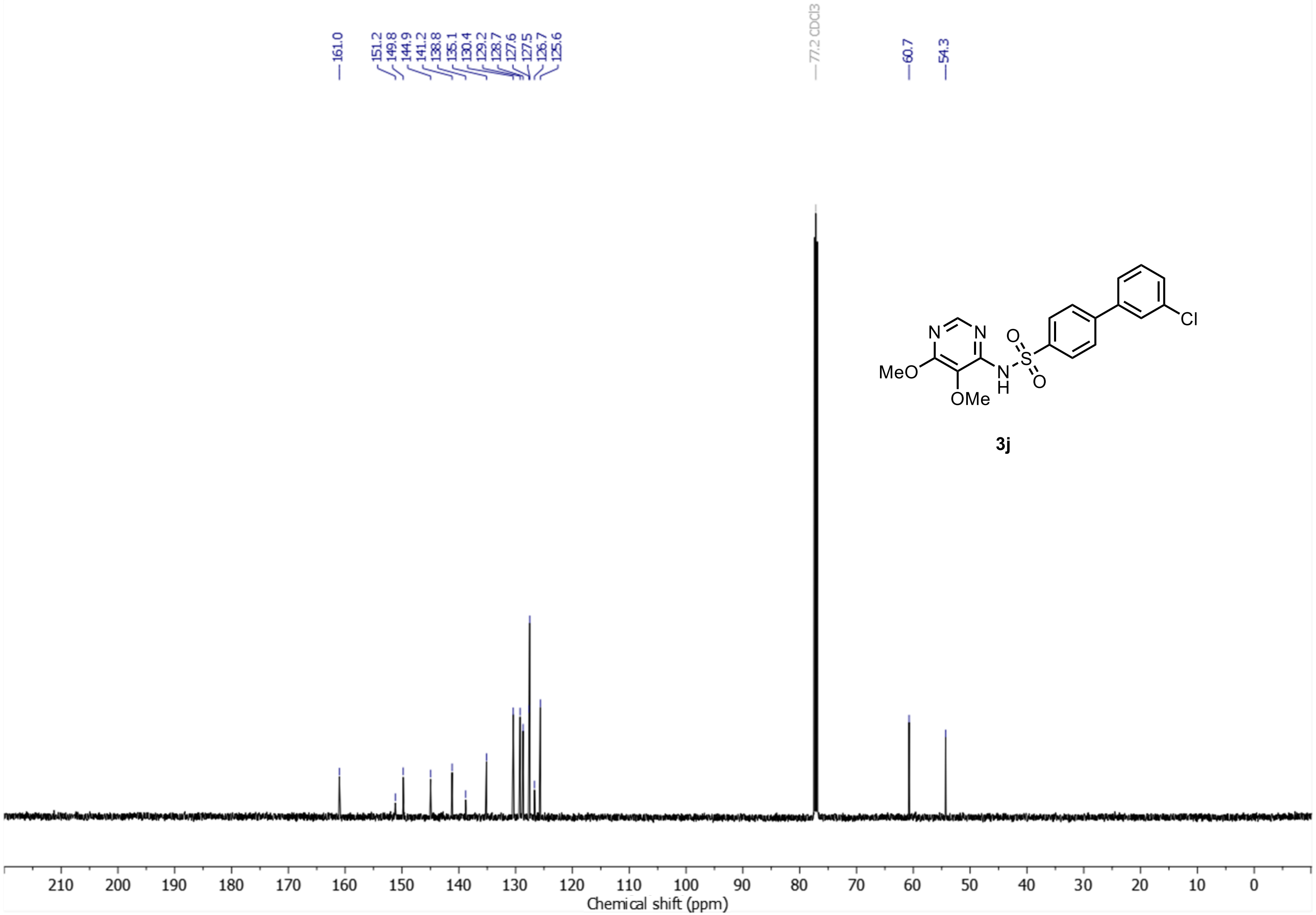

^{1}H NMR spectrum of 3k

CDCl$_3$, 500 MHz, 23 ºC

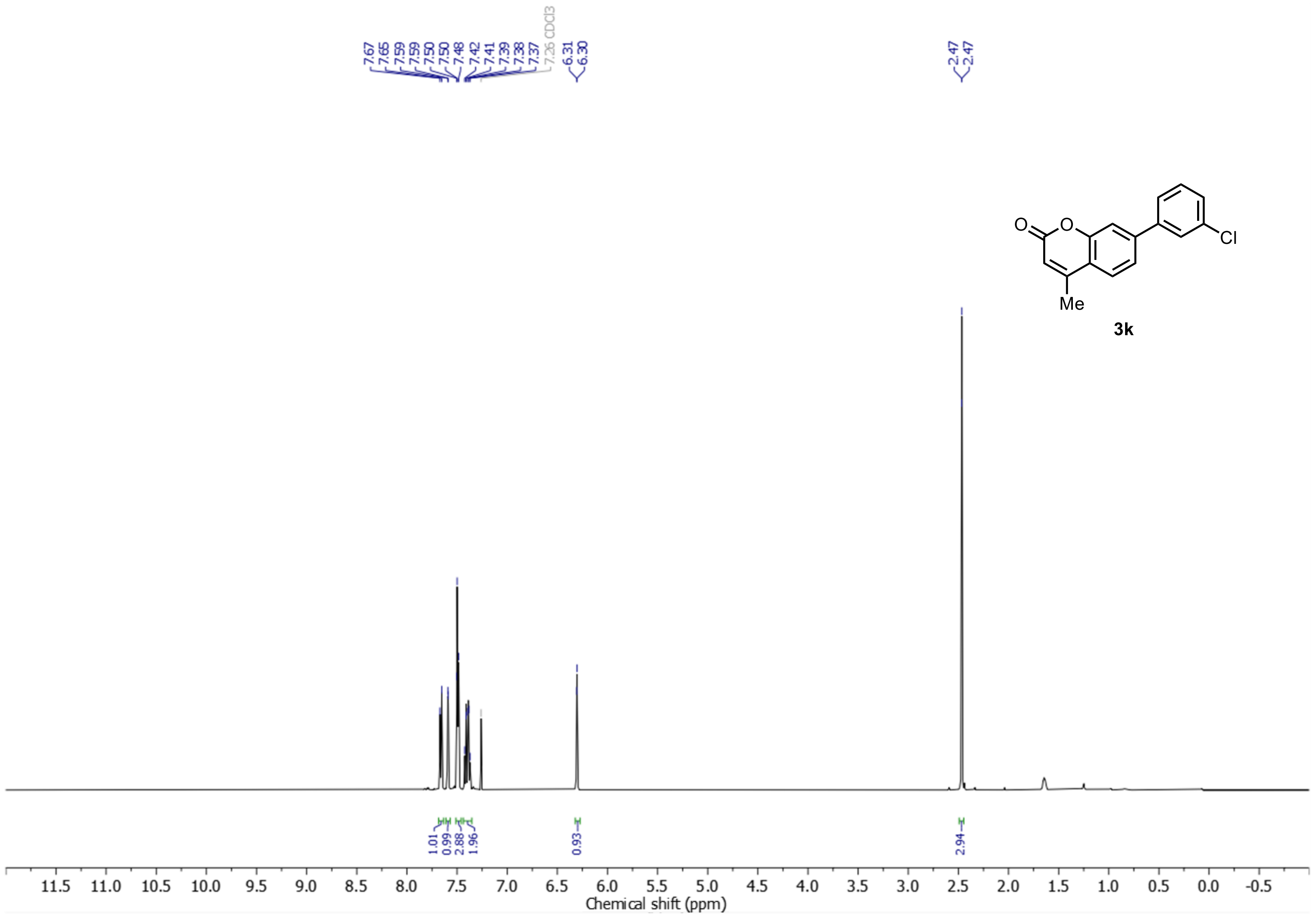

^{13}C NMR spectrum of 3k

$CDCl_3$, 125 MHz, 23 ºC

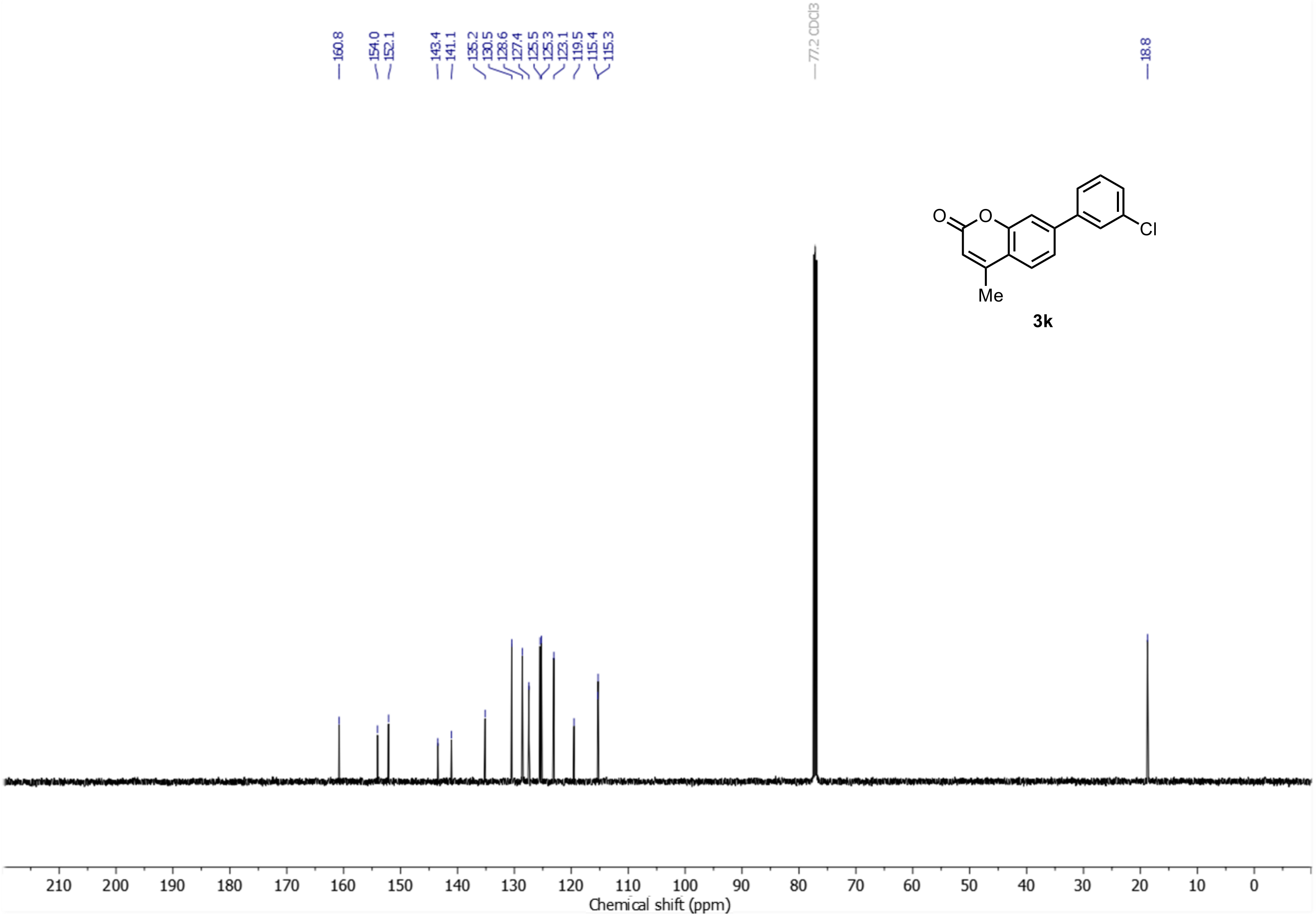

^{1}H NMR spectrum of 3l

$CDCl_3$, 500 MHz, 23 ºC

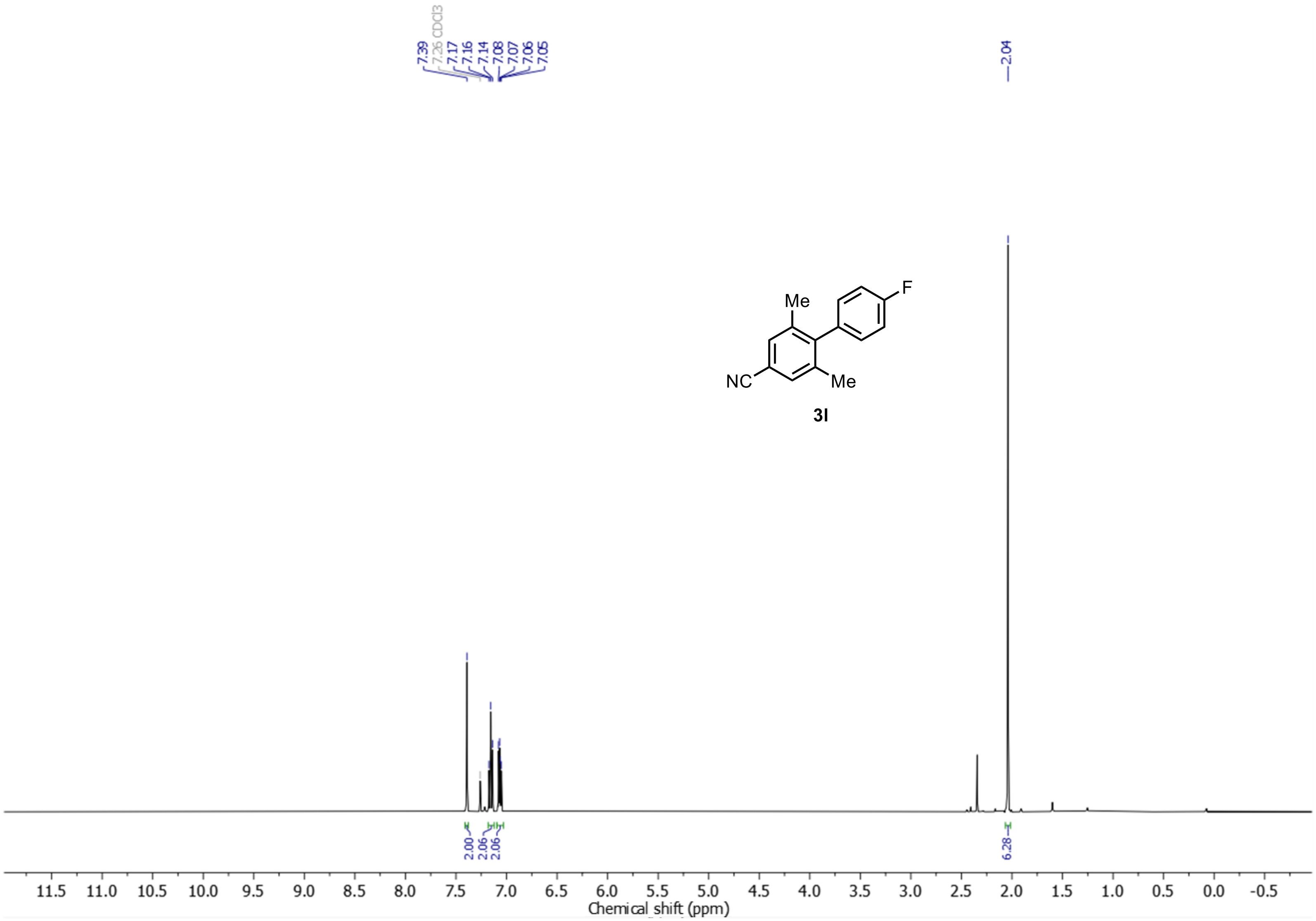

^{13}C NMR spectrum of 3l

$CDCl_3$, 125 MHz, 23 ºC

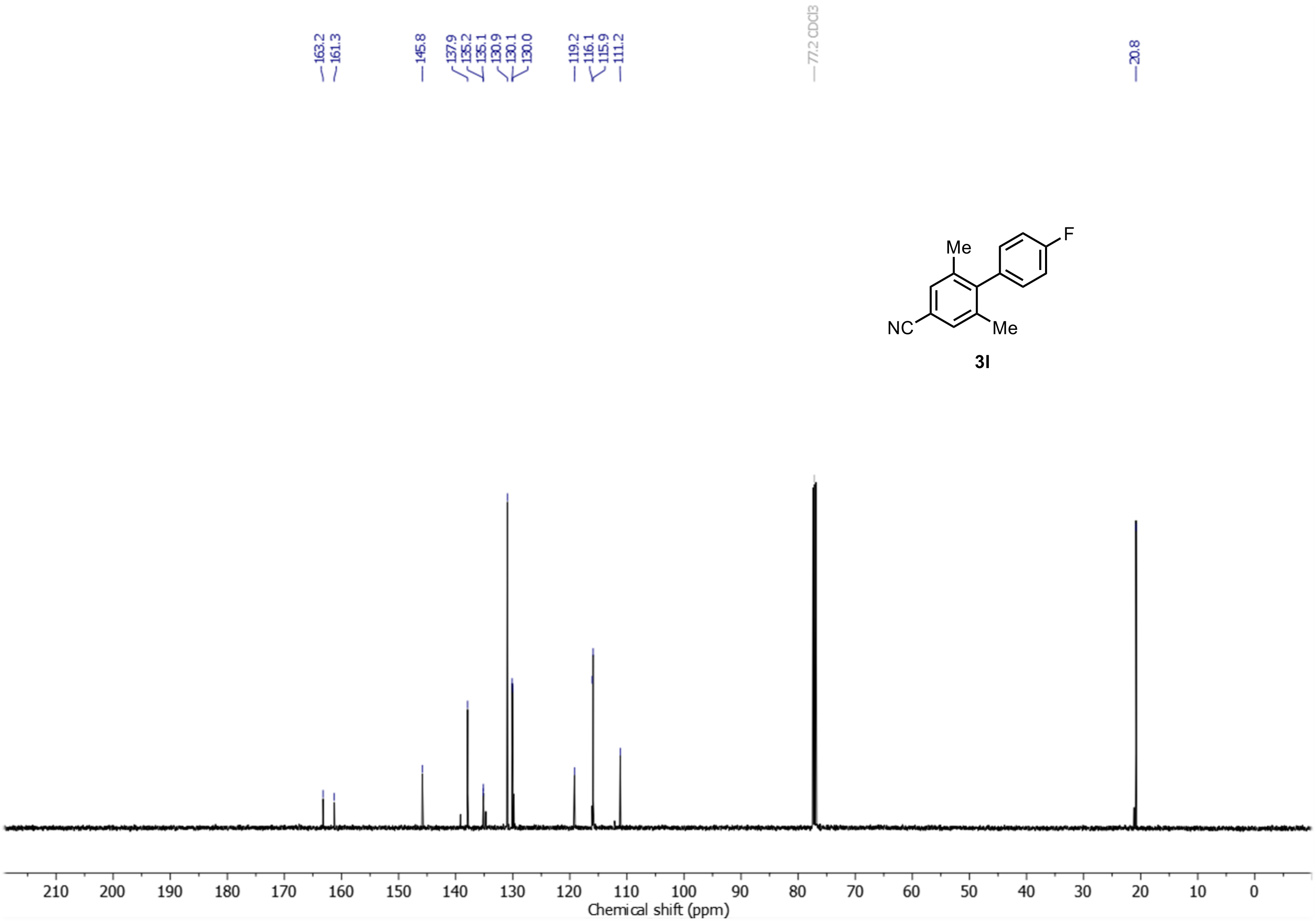

3l

^{1}H NMR spectrum of 3m

$CDCl_3$, 500 MHz, 23 ºC

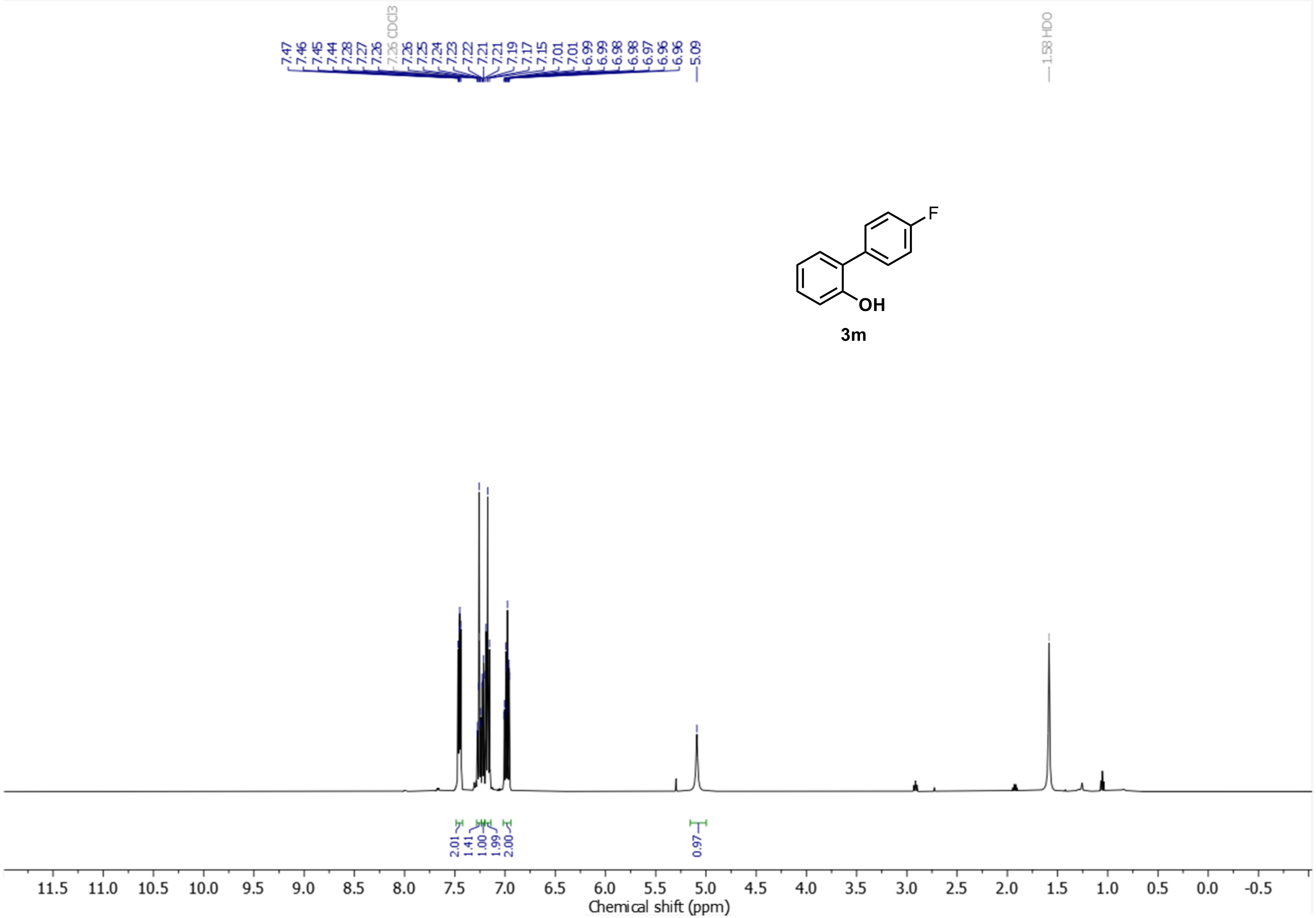

^{13}C NMR spectrum of 3m

$CDCl_3$, 125 MHz, 23 ºC

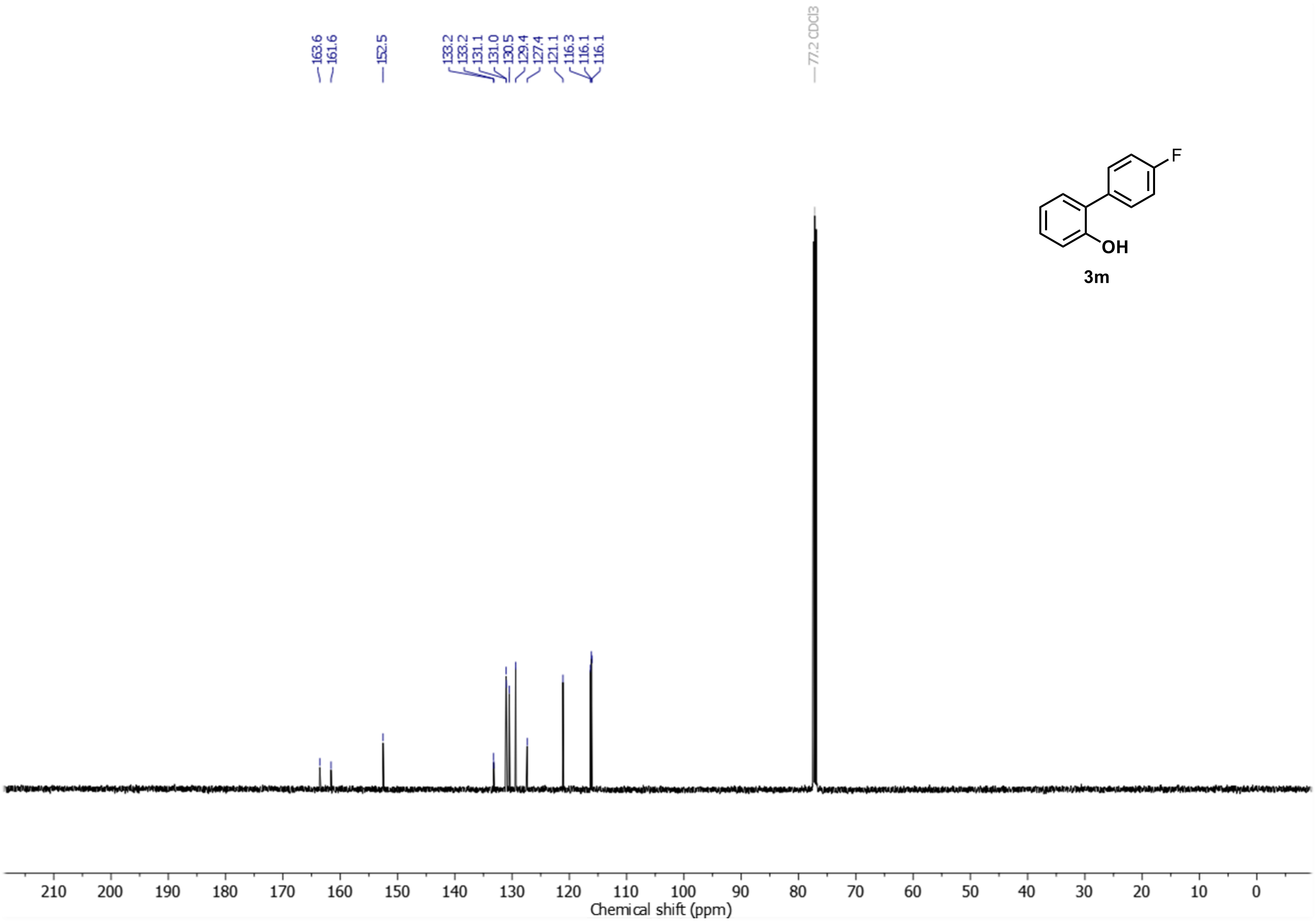

^{19}F NMR spectrum of 3m

$CDCl_3$, 471 MHz, 23 ºC

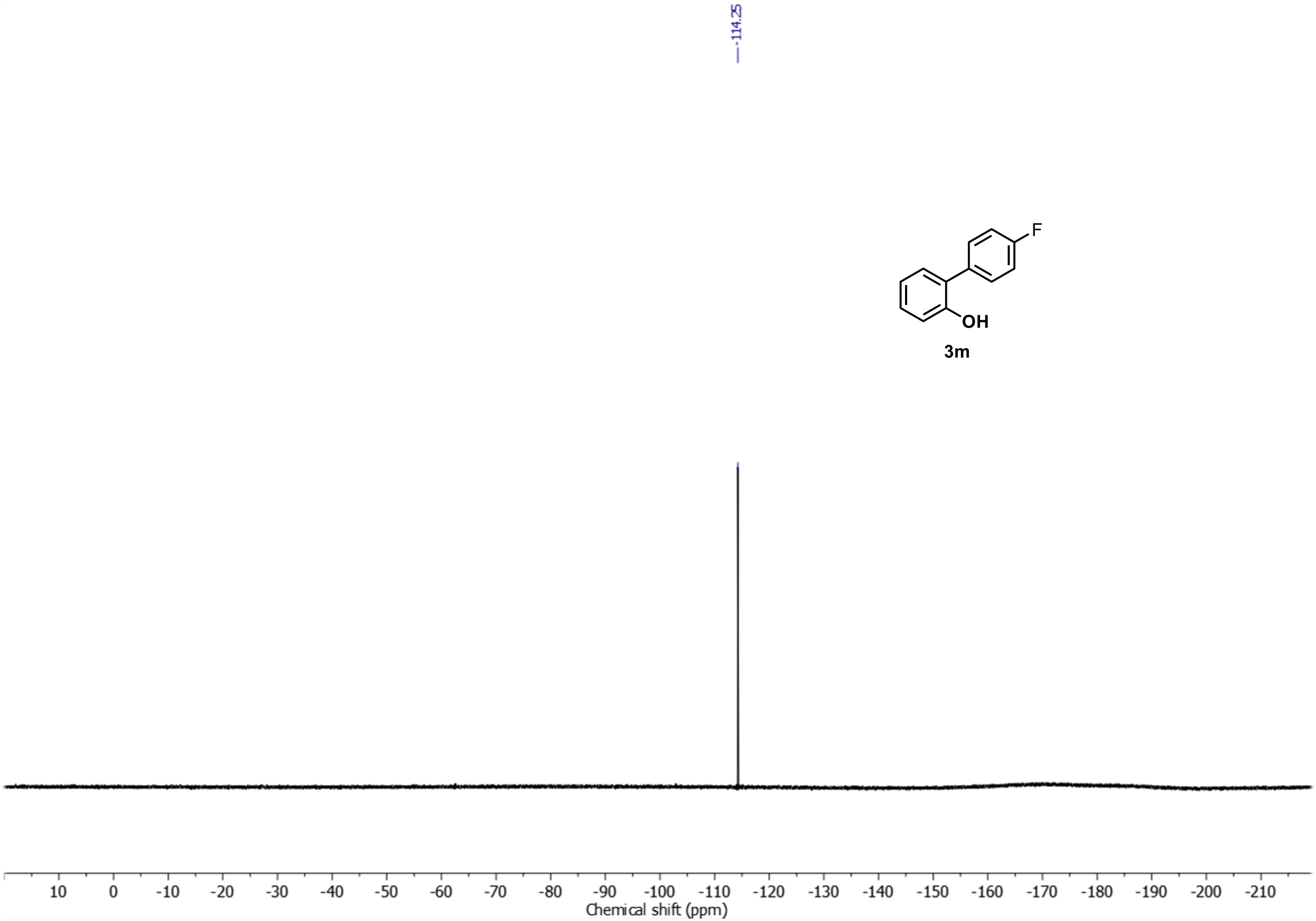

^{1}H NMR spectrum of 3n

$CDCl_3$, 500 MHz, 23 ºC

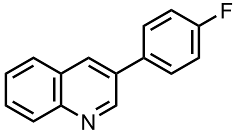

3n

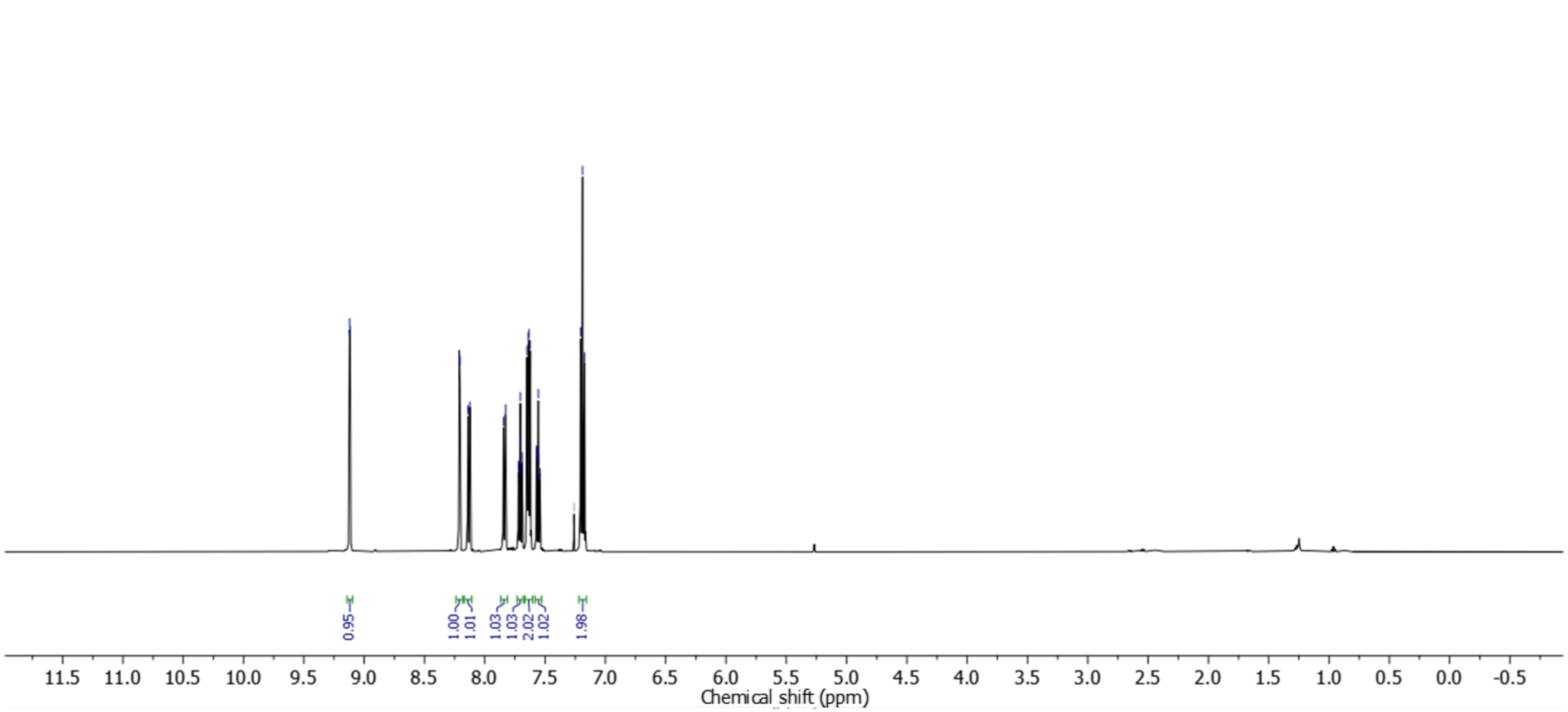

^{13}C NMR spectrum of 3n

$CDCl_3$, 125 MHz, 23 ºC

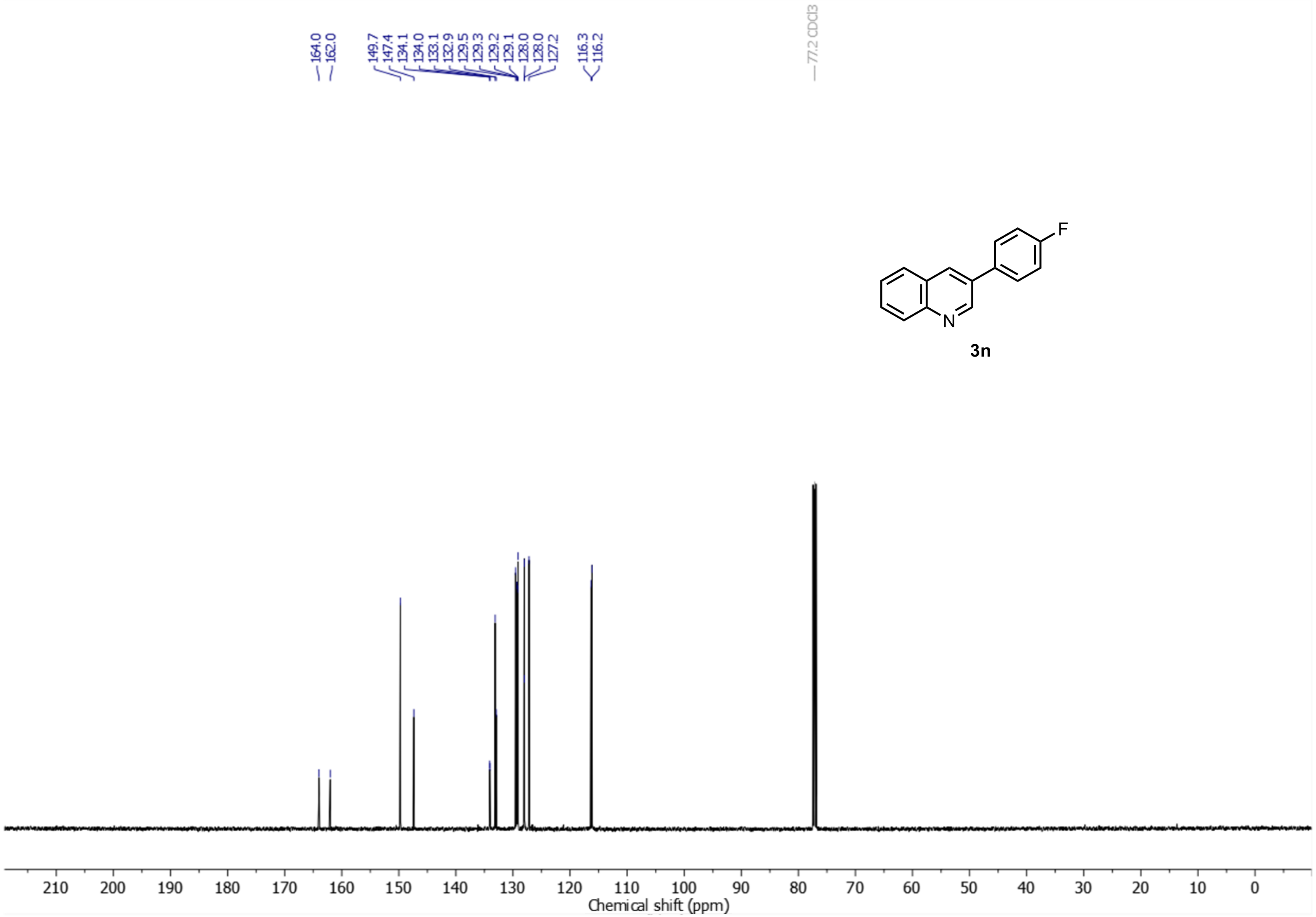

^{19}F NMR spectrum of 3n

$CDCl_3$, 471 MHz, 23 ºC

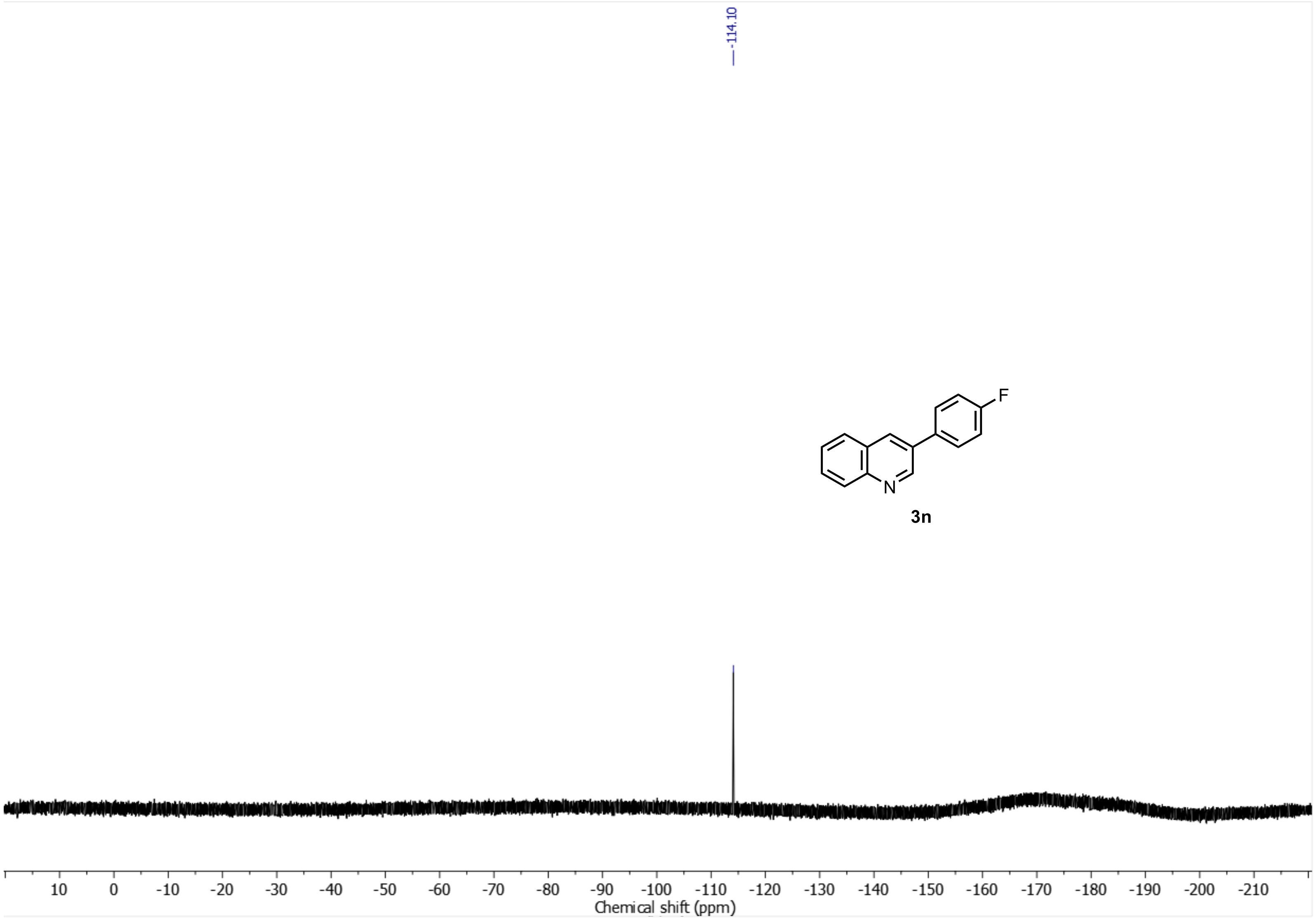

^{1}H NMR spectrum of 3o

$CDCl_3$, 500 MHz, 23 ºC

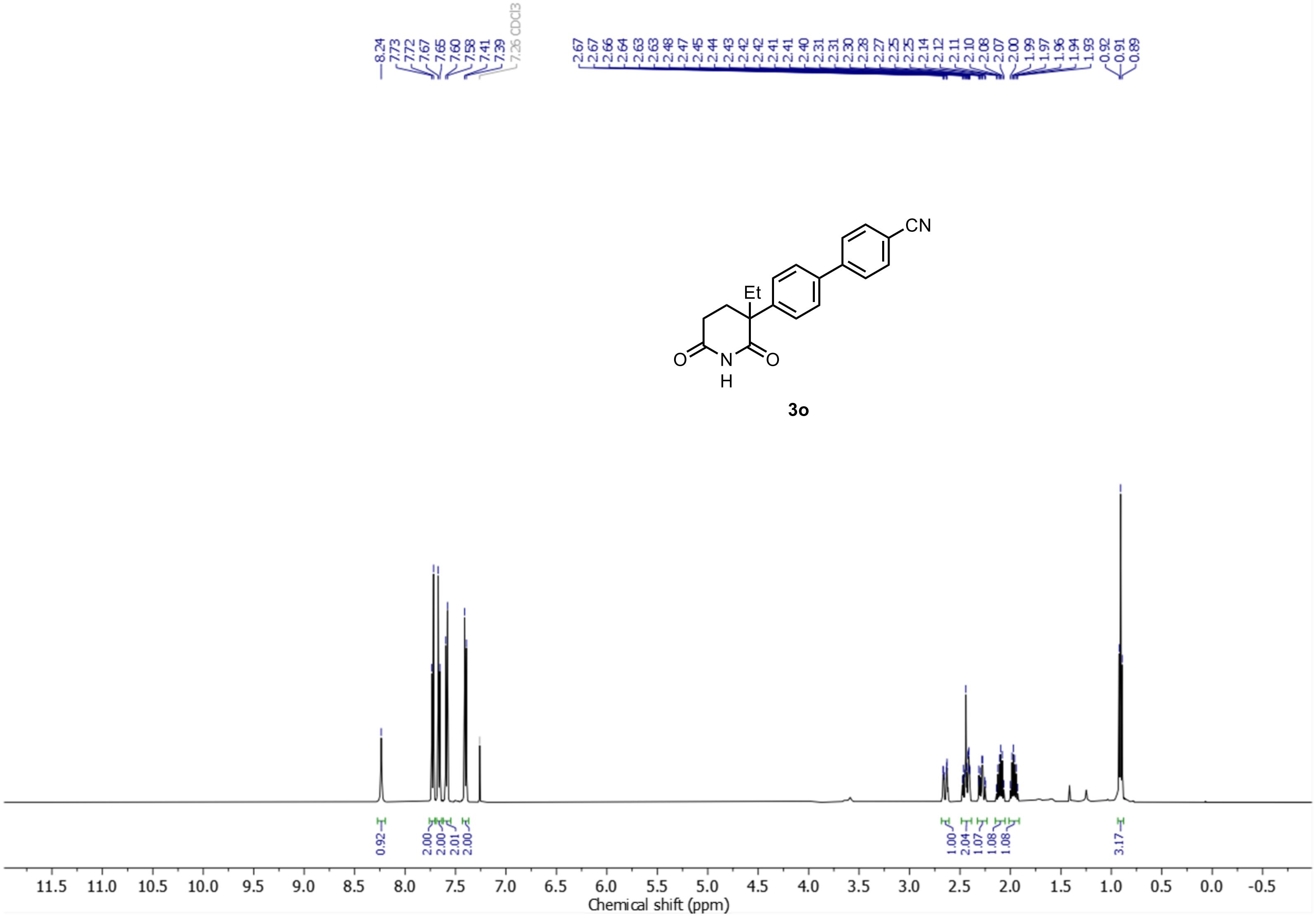

^{13}C NMR spectrum of 3o

$CDCl_3$, 125 MHz, 23 ºC

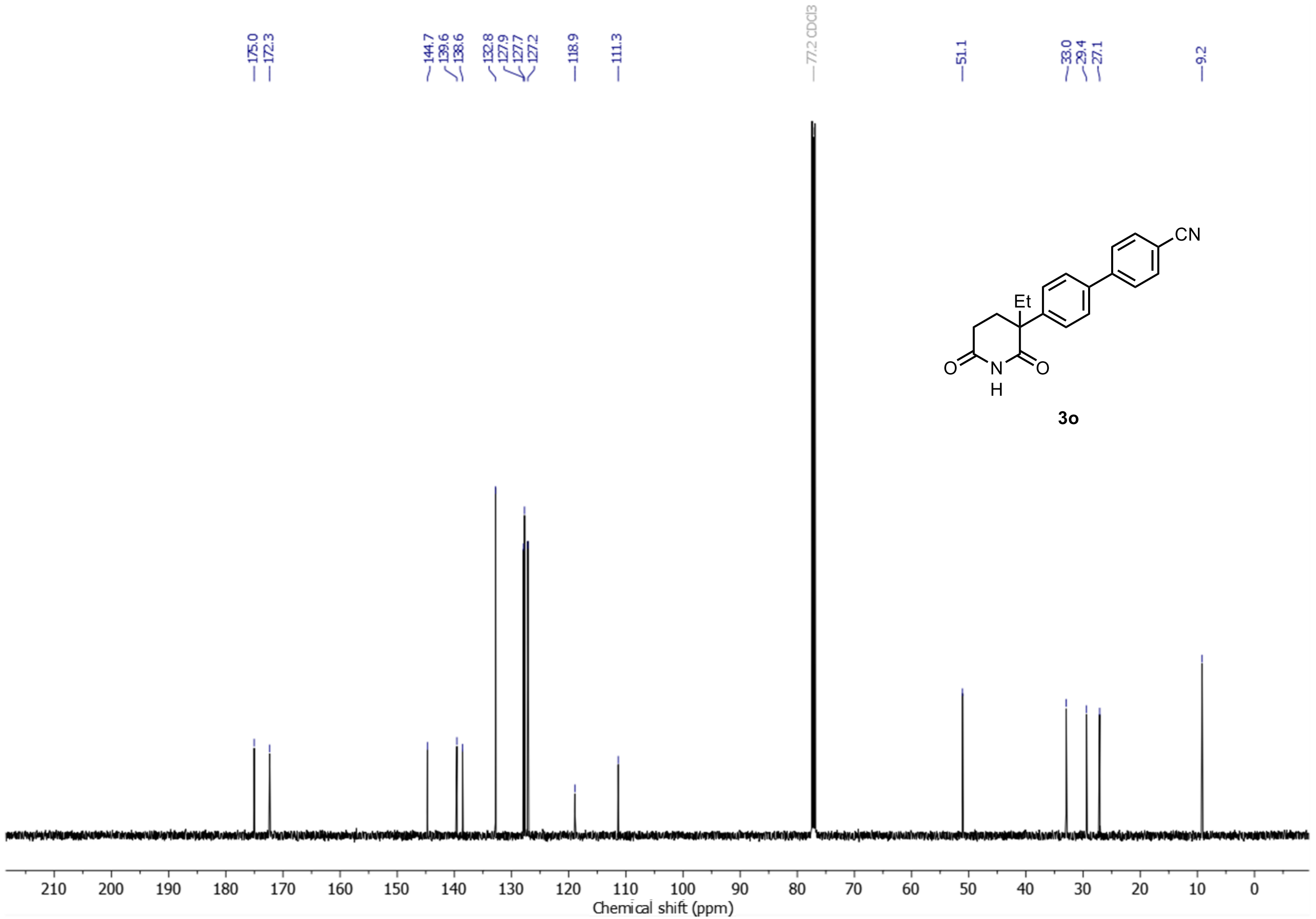

^{1}H NMR spectrum of 3p

$CDCl_3$, 500 MHz, 23 ºC

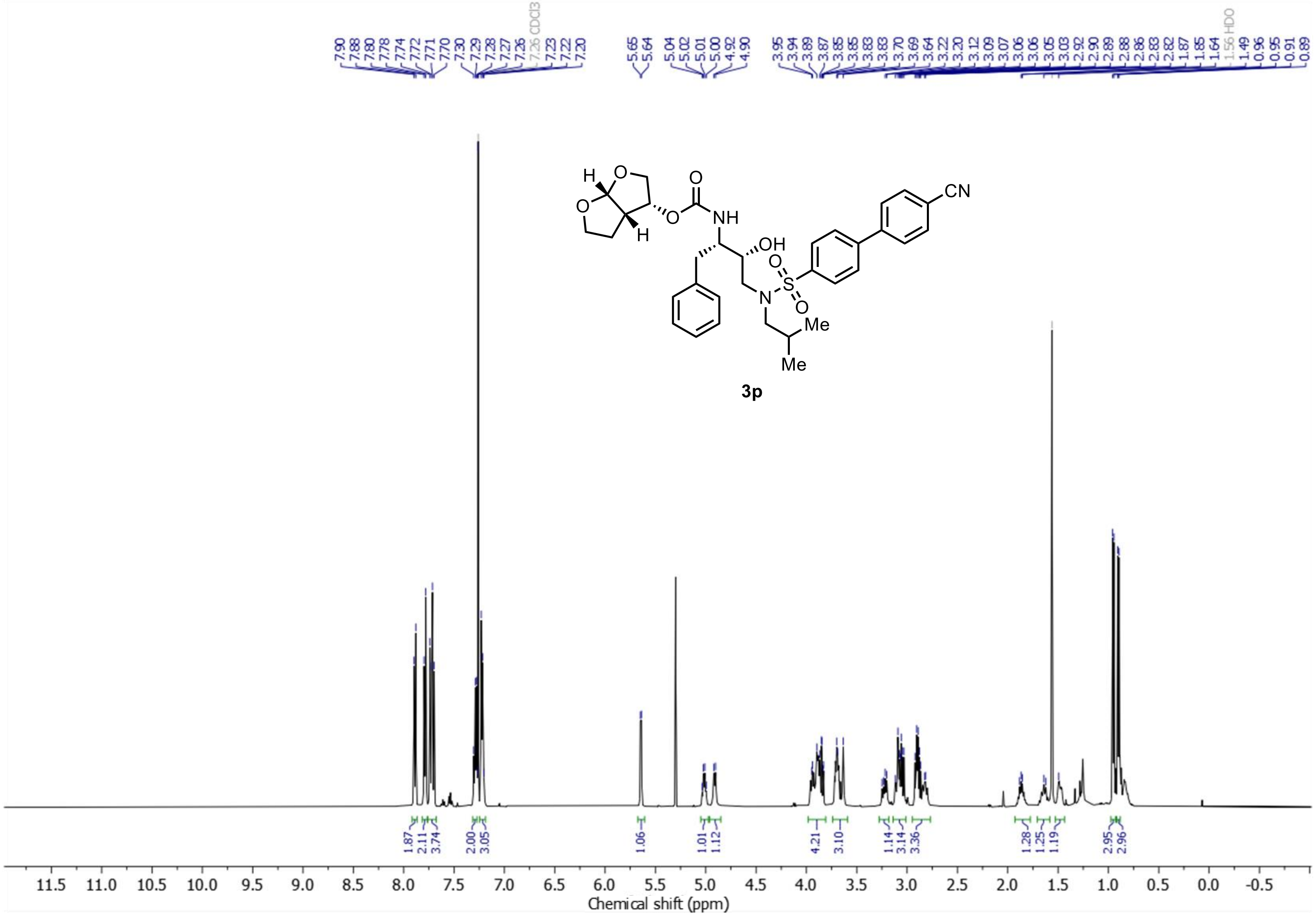

^{13}C NMR spectrum of 3p

$CDCl_3$, 125 MHz, 23 ºC

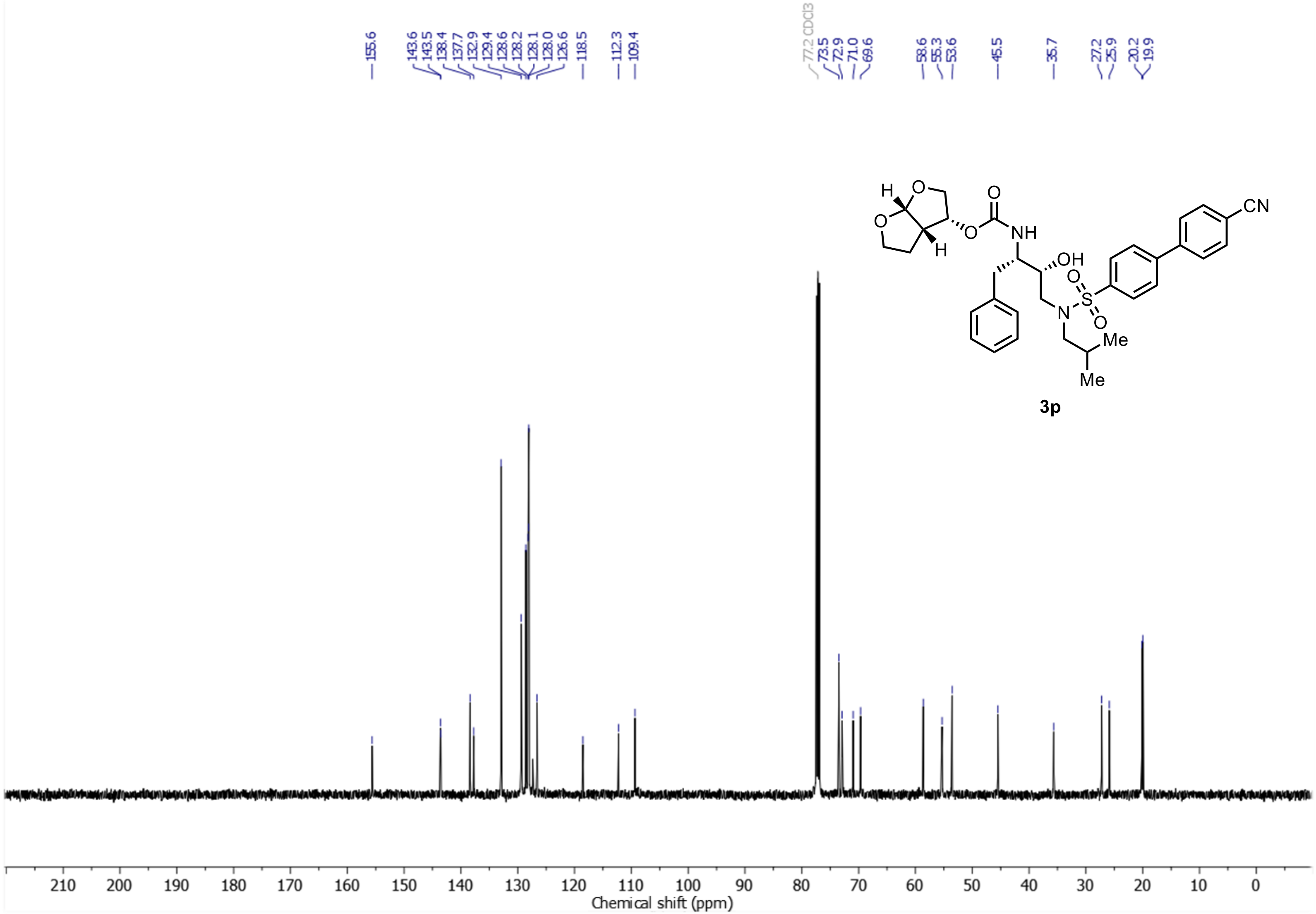

^{1}H NMR spectrum of 9a

$CDCl_3$, 500 MHz, 23 ºC

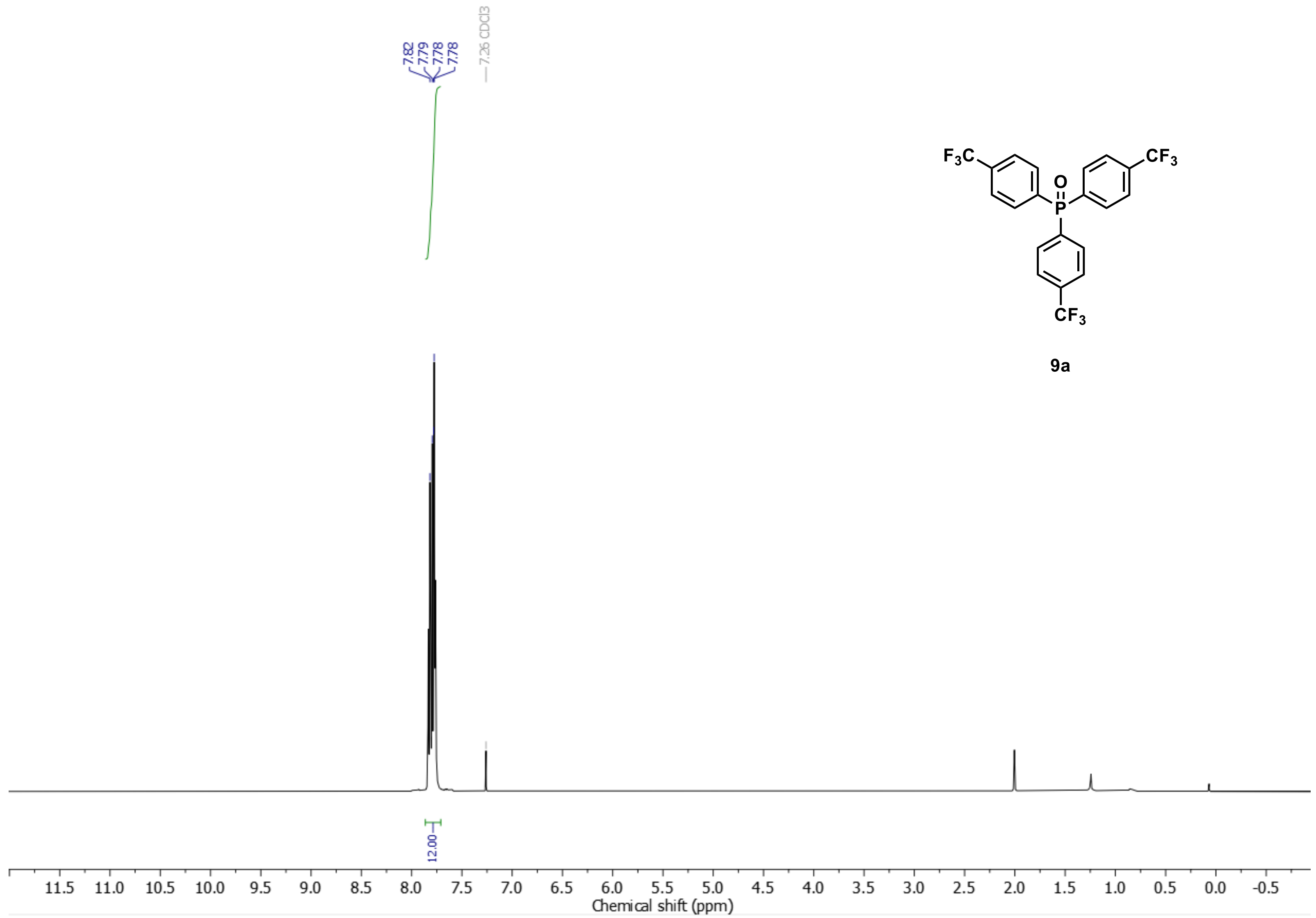

^{13}C NMR spectrum of 9a

$CDCl_3$, 125 MHz, 23 ºC

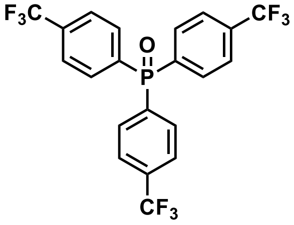

9a

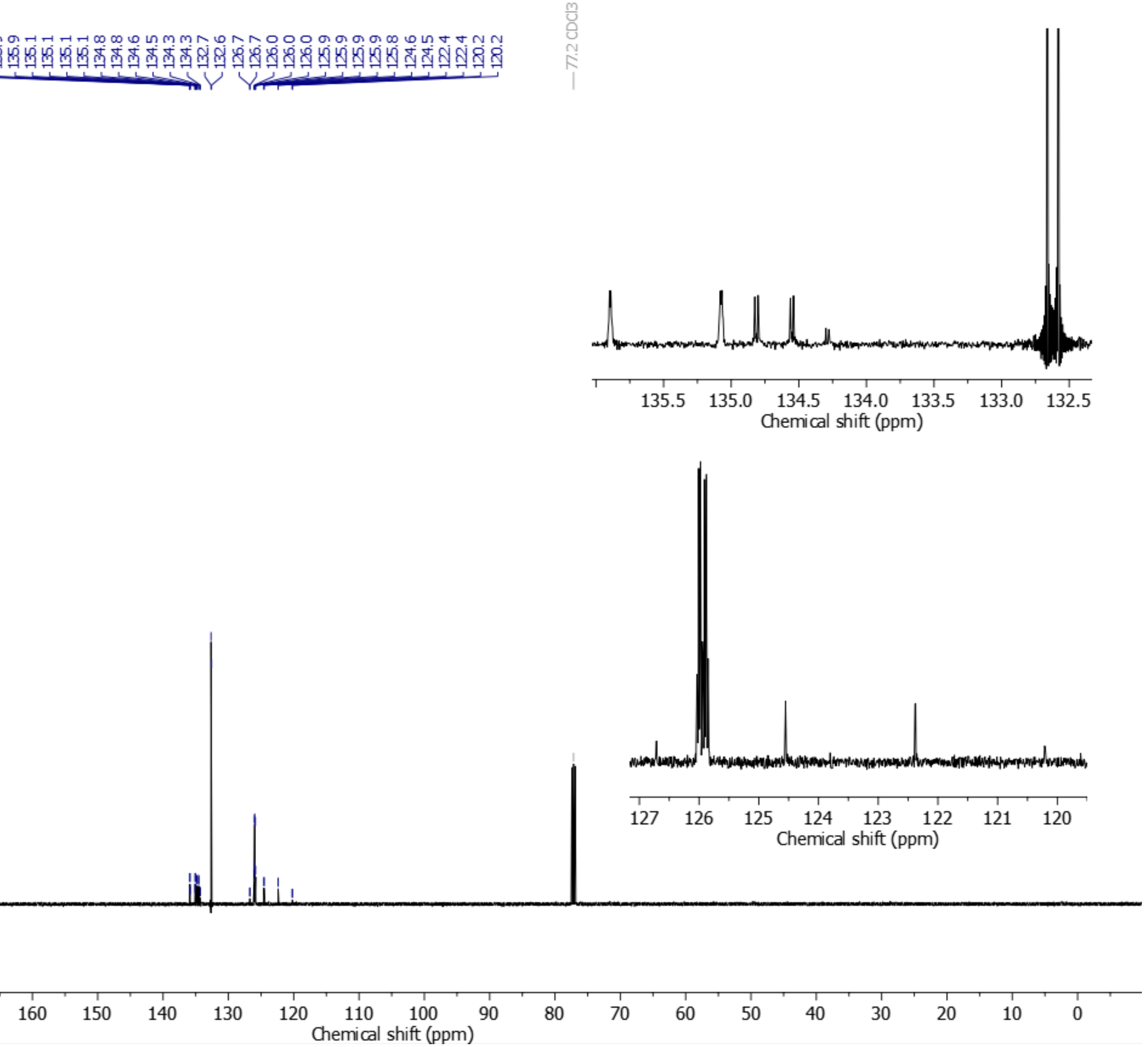

^{19}F NMR spectrum of 9a

$CDCl_3$, 471 MHz, 23 ºC

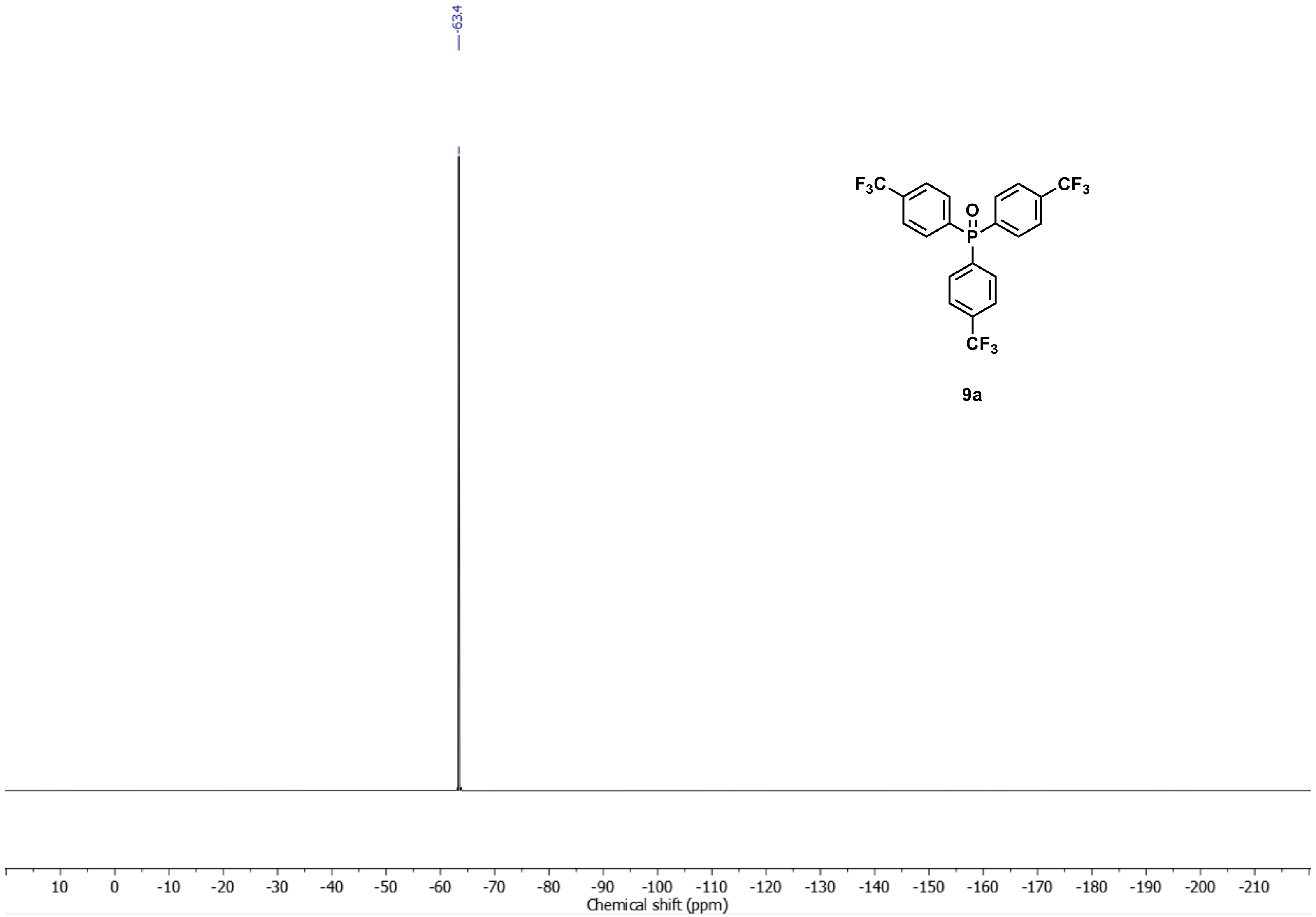

9a

^{31}P NMR spectrum of 9a

$CDCl_3$, 203 MHz, 23 ºC

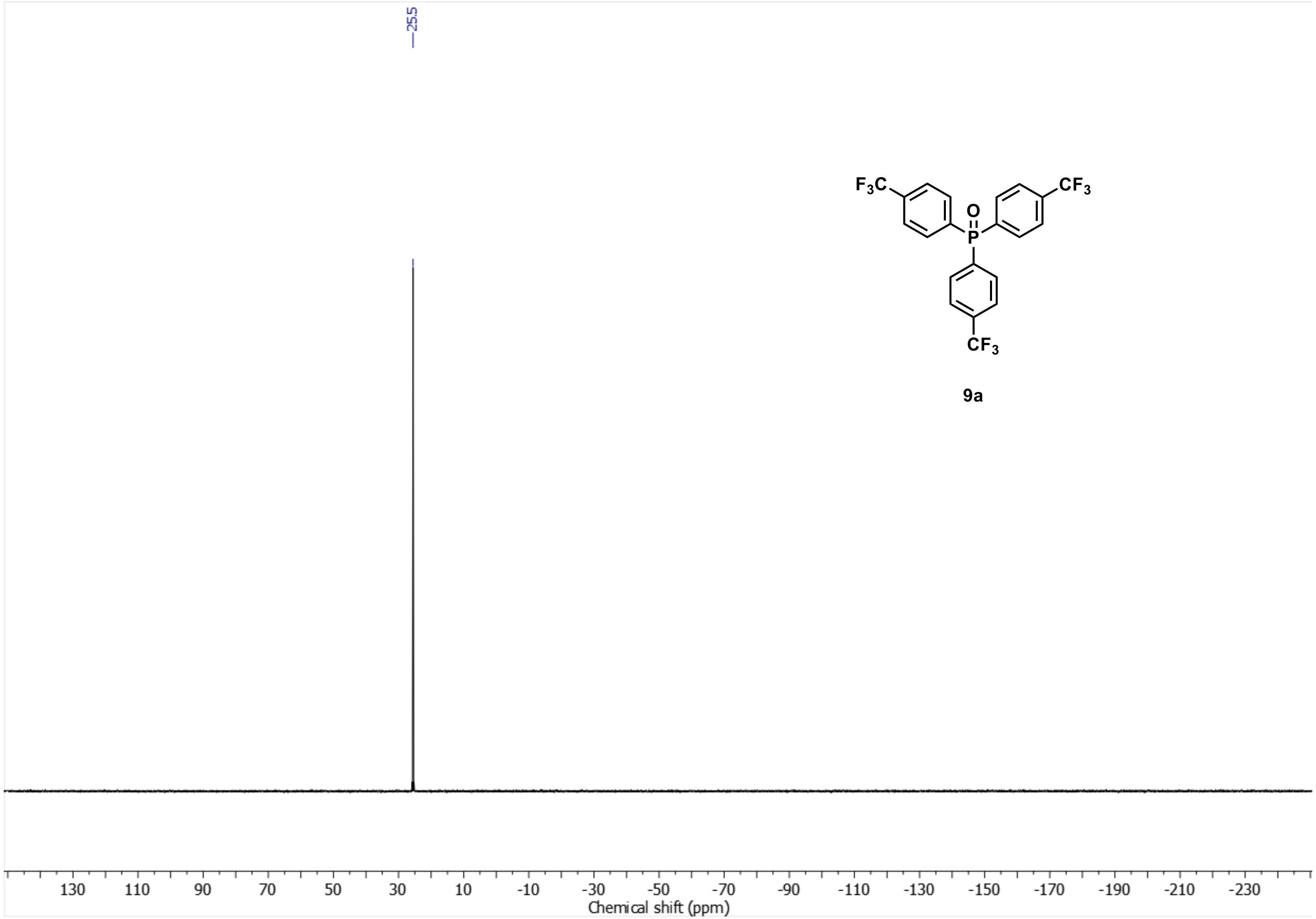

^{1}H NMR spectrum of 10a

MeCN-d_3, 500 MHz, 23 ºC

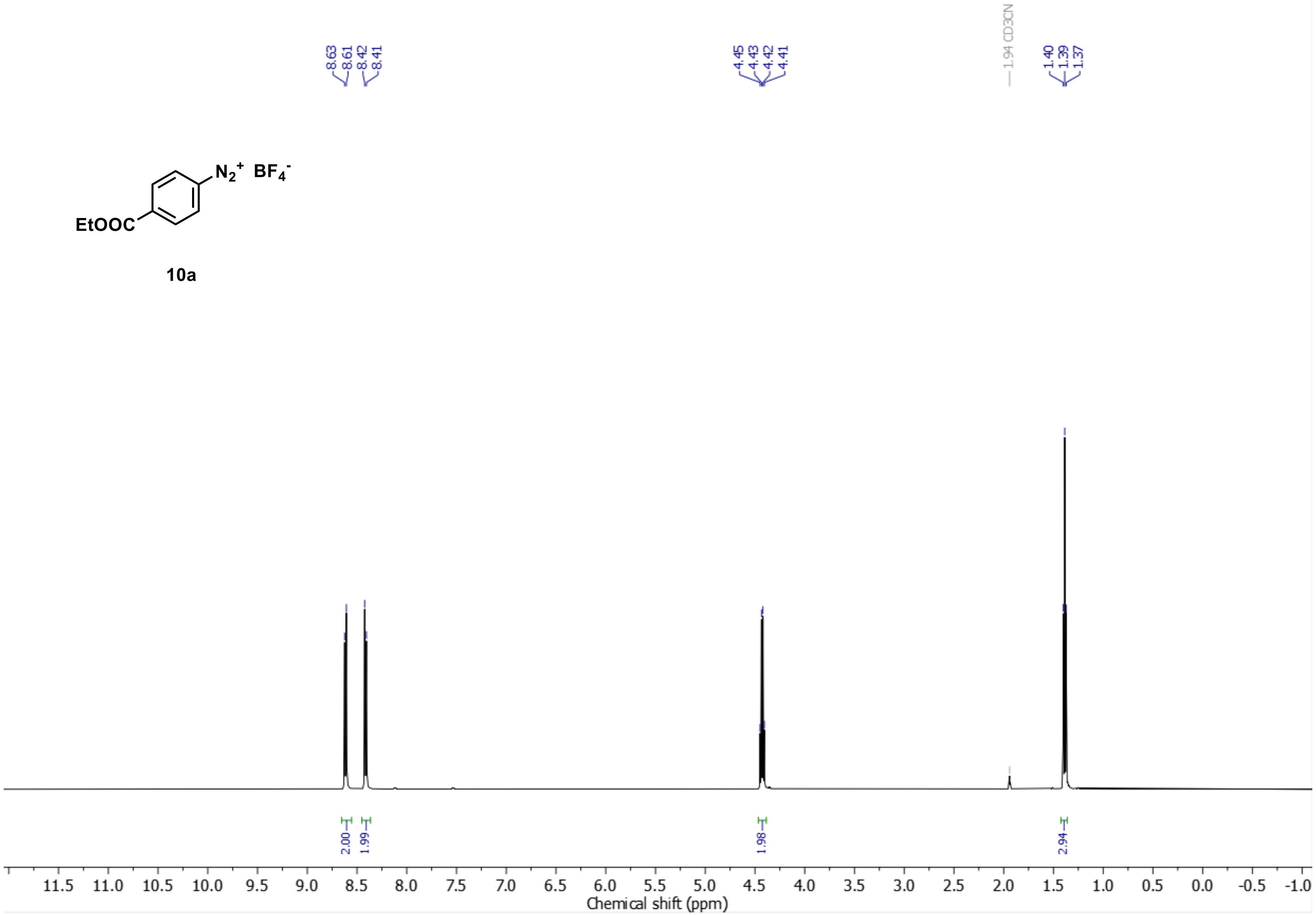

^{13}C NMR spectrum of 10a

MeCN-d_3, 125 MHz, 23 ºC

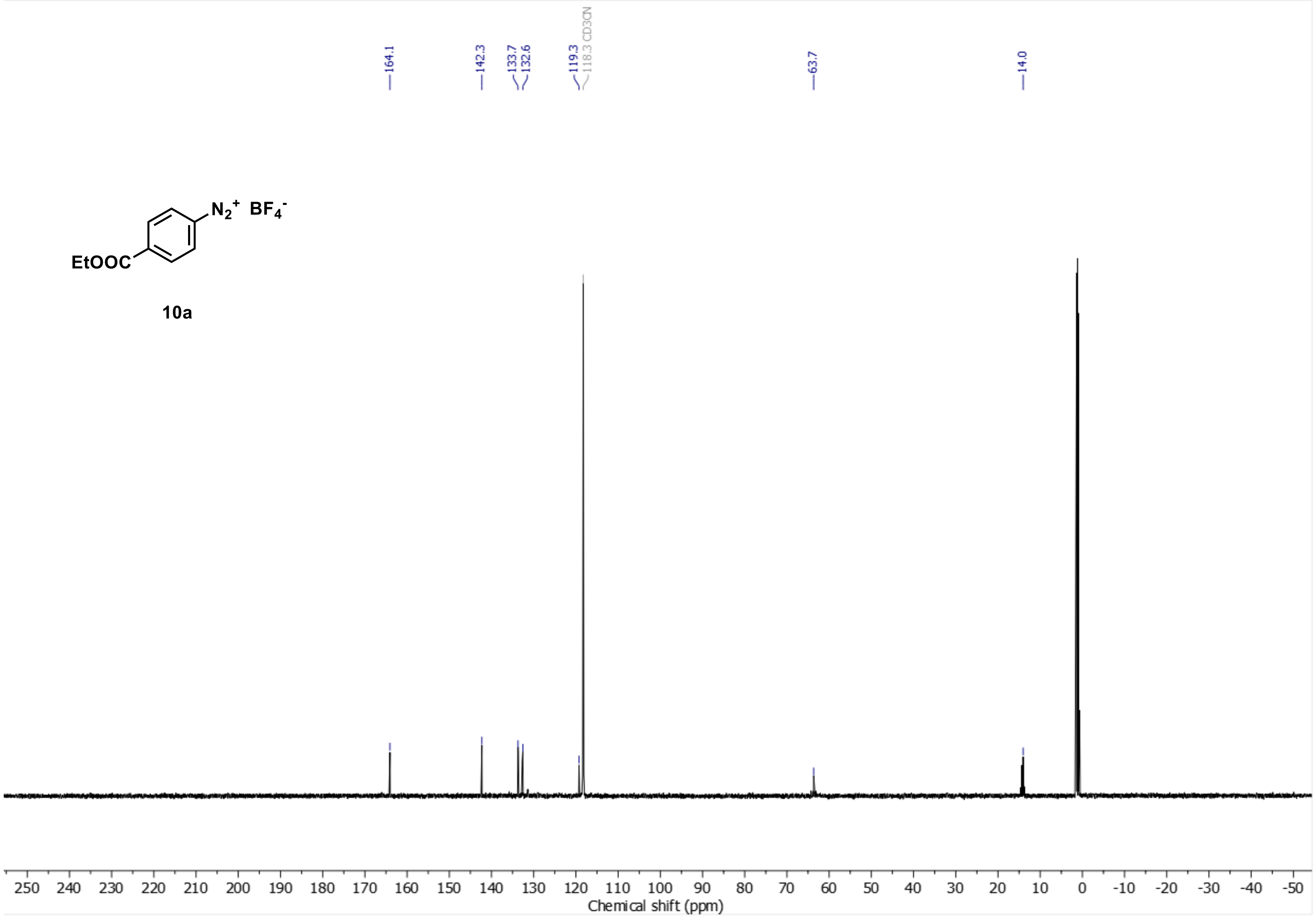

^{19}F NMR spectrum of 10a

MeCN-d_3, 471 MHz, 23 ºC

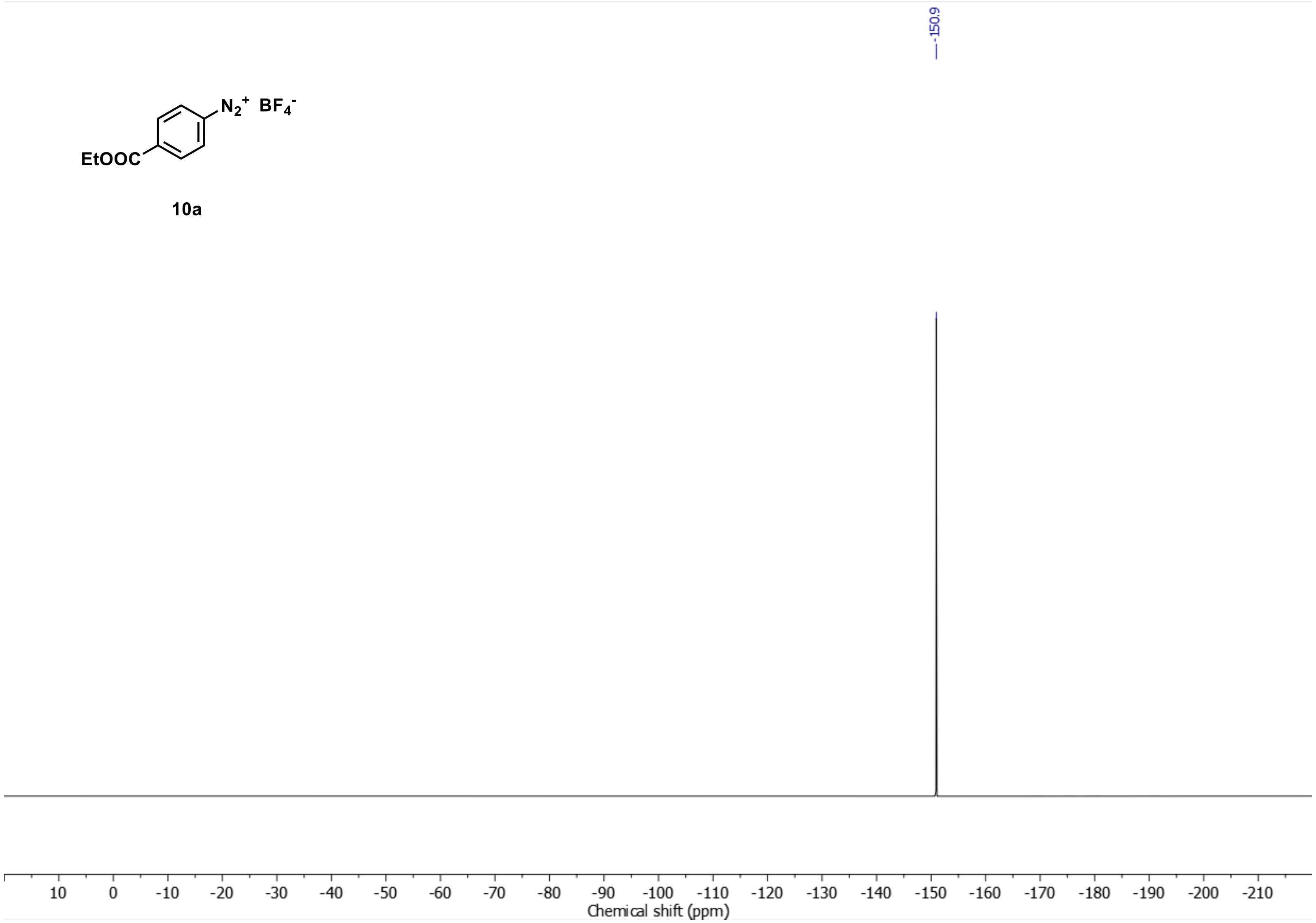

^{1}H NMR spectrum of 11a

$CDCl_3$, 500 MHz, 23 ºC

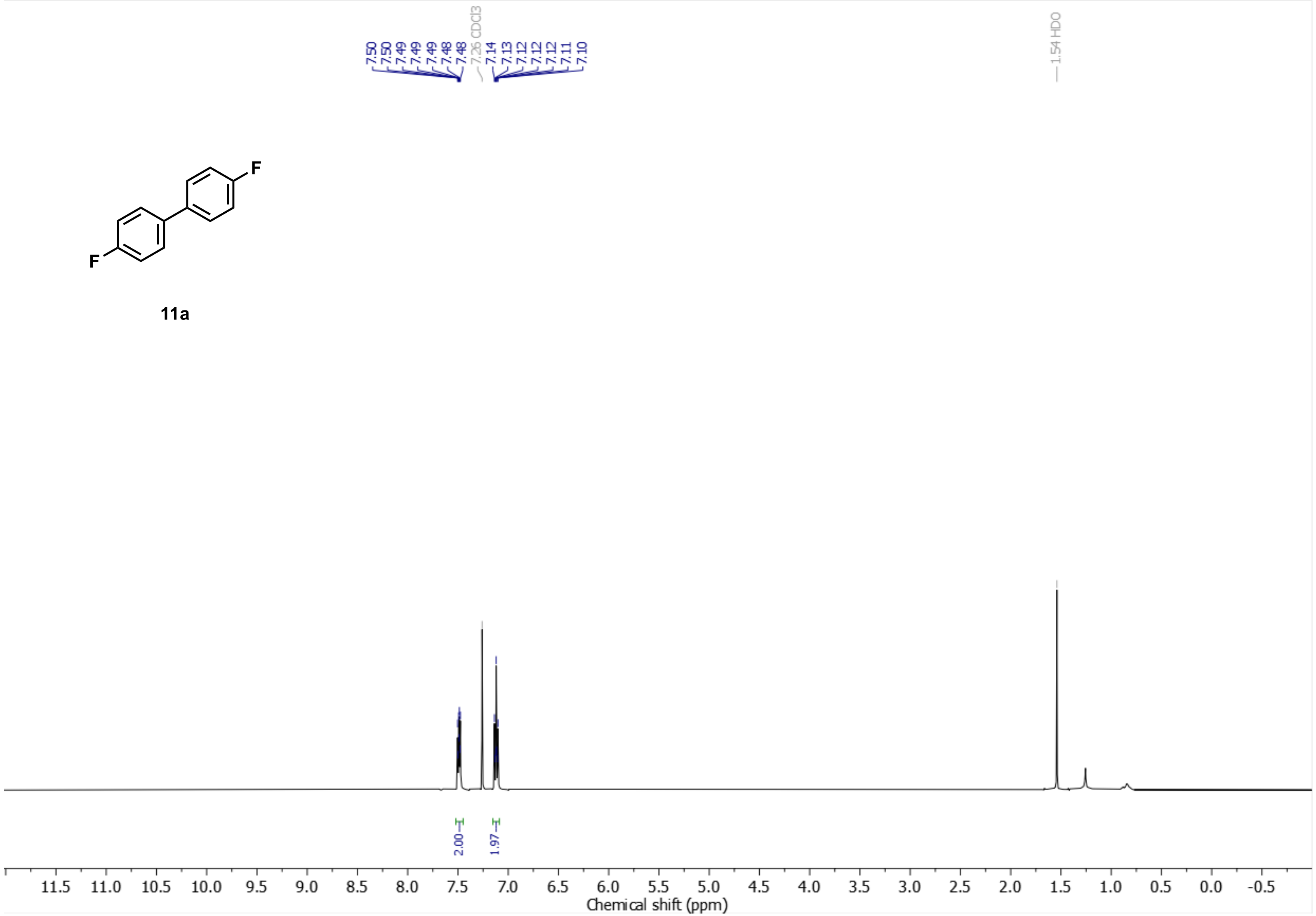

11a

^{13}C NMR spectrum of 11a

$CDCl_3$, 125 MHz, 23 ºC

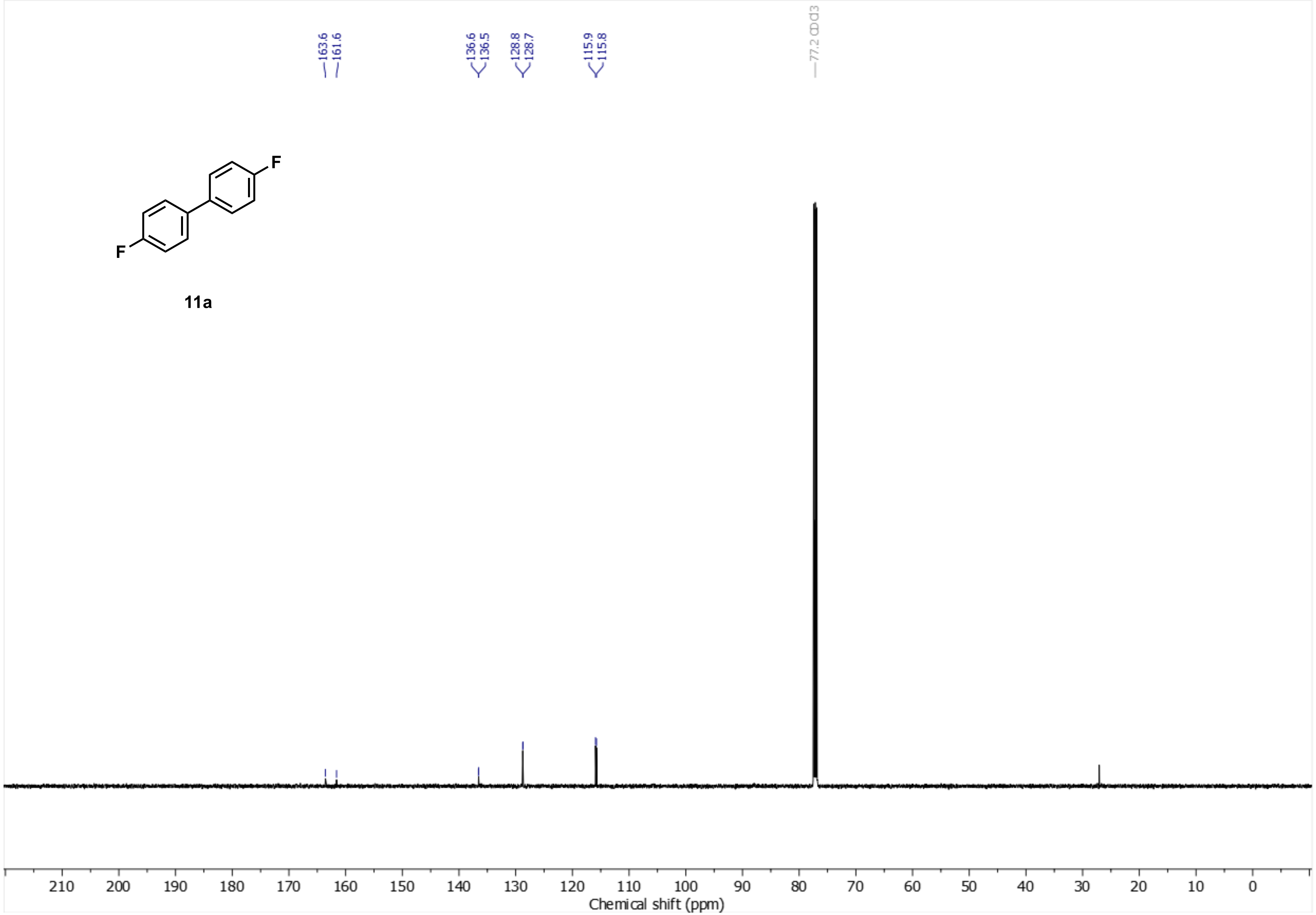

^{19}F NMR spectrum of 11a

$CDCl_3$, 471 MHz, 23 ºC

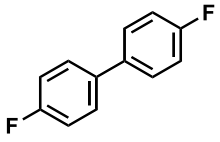

11a

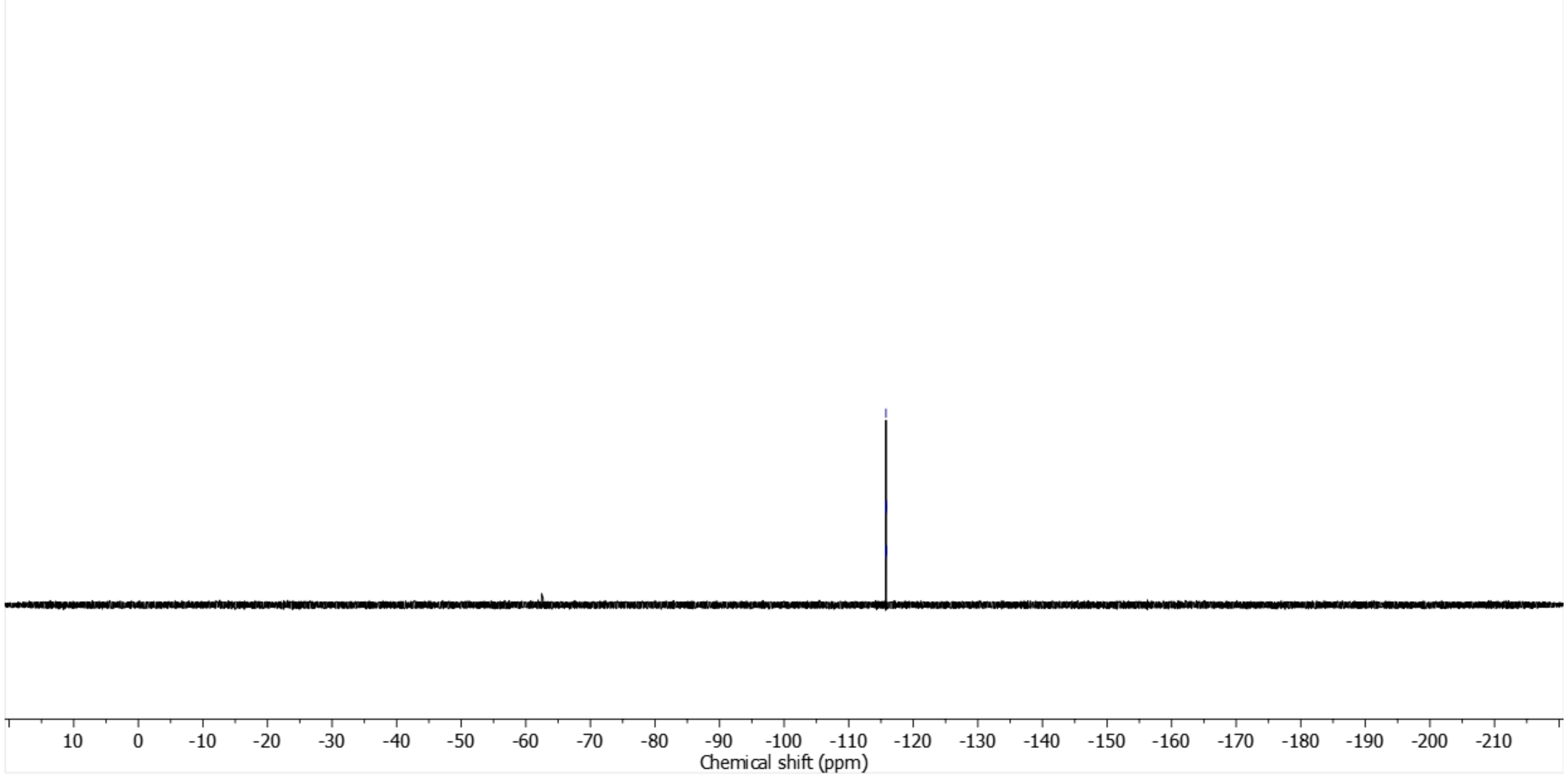

REFERENCES

[1] R. Cai, M. Lu, E. Y. Aguilera, Y. Xi, N. G. Akhmedov, J. L. Petersen, H. Chen, X. Shi, *Angew. Chem. Int. Ed.* **2015**, *54*, 8772.
[2] S. Yang, H. Li, X. Yu, J. An, M. Szostak, *J. Org. Chem.* **2022**, *87*, 15250.
[3] X. Yang, D. Lu, W. Guan, S.-F. Yin, N. Kambe, R. Qiu, *J. Org. Chem.* **2022**, *87*, 7720-7733.
[4] J. Mateos, T. Schulte, D. Behera, M. Leutzsch, A. Altun, T. Sato, F. Waldbach, A. Schnegg, F. Neese, T. Ritter, *Science* **2024**, *384*, 446-452.
[5] I. Katsounaros, D. Ipsakis, C. Polatides, G. Kyriacou, *Electrochimica Acta* **2006**, *52*, 1329-1338.
